# IgYs: on her majesty’s secret service

**DOI:** 10.3389/fimmu.2023.1199427

**Published:** 2023-06-12

**Authors:** Renata Grzywa, Agnieszka Łupicka-Słowik, Marcin Sieńczyk

**Affiliations:** Division of Organic and Medicinal Chemistry, Faculty of Chemistry, Wrocław University of Science and Technology, Wroclaw, Poland

**Keywords:** IgY, antibaceterial, avian antibodies (IgY), infection, antigens, hyperimmune

## Abstract

There has been an increasing interest in using Immunoglobulin Y (IgY) antibodies as an alternative to “classical” antimicrobials. Unlike traditional antibiotics, they can be utilized on a continual basis without leading to the development of resistance. The veterinary IgY antibody market is growing because of the demand for minimal antibiotic use in animal production. IgY antibodies are not as strong as antibiotics for treating infections, but they work well as preventative agents and are natural, nontoxic, and easy to produce. They can be administered orally and are well tolerated, even by young animals. Unlike antibiotics, oral IgY supplements support the microbiome that plays a vital role in maintaining overall health, including immune system function. IgY formulations can be delivered as egg yolk powder and do not require extensive purification. Lipids in IgY supplements improve antibody stability in the digestive tract. Given this, using IgY antibodies as an alternative to antimicrobials has garnered interest. In this review, we will examine their antibacterial potential.

## Introduction

Antimicrobial resistance (AMR) has become a worldwide threat to human and animal health, leading to increased mortality, longer hospitalizations, increased costs of medical treatment, and food and agriculture security manifested by elevated abundance and transfer of antibiotic resistance genes (ARGs) between various species [WHO, 2020 (1)]. Although AMR mostly relates to antivirals, antifungals, and antiparasitics, antibiotics also constitute an important part of the problem. As reported by Cassini et al., antibiotic resistance accounts for approximately 33,000 deaths annually in the European Union alone (2). The problem of the increasing antibiotic resistance, the prevention of its spread, and the need for constant monitoring of antibiotic consumption became the concern of the WHO initiative known as the Global Antimicrobial Resistance and US Surveillance System launched in 2015, in which 109 countries participated in the 2021 (3).

The emergence of AMR has been triggered by a soaring antibiotic uptake that began to be unnecessarily prescribed at the initial treatment of nonbacterial infections. This practice resulted in various respiratory tract infections (4). AMR communities fall easy prey to infections such as tuberculosis (TB) or typhoid fever (5). A great part of antibiotics is administered to animals to treat and control diseases (6). Antibiotics are used not only for treatment but also for prevention, which leads to the accumulation of antibiotic metabolites in animal products consumed by humans. These, in turn, might be either not tolerated by humans or might increase the transfer of ARGs (7, 8).

Among the proposed ways to address the problem of AMR resulting from the irrational use or overuse of antibiotics in both human medicine and agriculture is to strengthen the regulation of the distribution, dosage, and production of antibiotics (9). Another objective is the diversification and introduction of new types of antimicrobial compounds. The Drugs for Neglected Diseases Initiative called the Global Antibiotic Research and Development Partnership aims to develop and deliver new treatments against drug-resistant bacteria defined by the WHO as the biggest threat: *Klebsiella pneumoniae*, *Escherichia coli*, *Staphlylococcus aureus*, *Neisseria gonorrhoeae*, and *Mycobacterium tuberculosis*. In recent years, many substances have been examined as substitutes for antibiotics such as antimicrobial peptides (10), liposomes [Combioxin SA, NCT02583373 (11)], natural extracts (12, 13), and mammalian antibodies (MedImmune LLC, NCT02696902; Aridis Pharmaceuticals, Inc., NCT03027609). In addition, the use of IgY antibodies as an alternative to antimicrobials is of general interest. In this review, we will focus on the IgY antibodies displaying the antibacterial potential.

*IgY technology* as a method of producing specific antibodies for therapy, prophylaxis, and diagnostics is well documented. The first report concerning the protective effect of egg yolk extracts from immunized hens against tetanus toxin (*Clostridium tetani*) in mice dates back to 1893 (14). Over 100 years later, specific IgY antibodies are being investigated under several clinical trials against bacterial infections including *Helicobacter pylori* (Immunology Research Institute in Gifu, NCT02721355), *K. pneumoniae* and *E. coli* (Regents of the University of Michigan, 2017-002110-32), *Clostridium difficile* (ImmuniMed Inc., NCT04121169), or *Pseudomonas aeruginosa* (Mukoviszidose Institut gGmbH, NCT01455675).

## What enables IgYs to function as antibacterial compounds?

IgY antibodies are considered the evolutionary ancestors of IgGs and are found in reptiles, amphibians, and birds (15). Ubiquity and ease of breeding and egg laying efficiency have made chickens the main source of obtaining IgYs. Because maternal serum antibodies accumulate in egg yolk, yolk is an efficient source of IgY (16). Antibodies derived from chicken egg yolks do not possess the hinge region in their structure; instead, they have an additional domain within the heavy chain (15). This characteristic organization of IgY domains along with a high content of proline and glycine residues makes heavy chains less flexible as compared to mammalian IgG. It can influence the resistance of the antibodies to proteolytic degradation but may, nevertheless, be fragmented by proteases (17). IgYs, unlike mammalian IgG, do not activate the antibody-mediated immune response in mammals (complement, rheumatoid factor, or Fc receptors) (18). An important limitation of IgY as therapeutic agents is its relatively low stability at low pH: They remain stable at a pH range between 3.5 and 11, whereas IgG at the range between 2 and 11 (19). However, the pH stability of IgY can be raised by sorbitol solutions (20) or encapsulation by liposomes (21). Another important characteristic of IgY antibodies is their temperature stability. The specific binding of IgYs decreases with increasing temperature: IgYs are stable at up to 70°C, whereas mammalian IgG can remain active even at slightly higher temperatures of up to 75°C–80°C (19, 22). High levels of sucrose, maltose, glycerol, or glycine as additives could improve IgYs temperature stability (22).

The primary organs responsible for the production of antibodies in birds are the bursa of Fabricius (diverticular fragment of the cloaca that plays a major role in the production of B lymphocytes and the differentiation of antibodies), the thymus (as in mammals, T lymphocytes, maturation site), and bone marrow. Peripheral organs of the immune system include the spleen, lymph nodes, and lymphoid tissue associated with mucous membranes, including Harder’s glands. B-cell precursor cells proliferate for about the first 2 months of an animal’s life and then migrate from the bursa of Fabricius to the thymus and spleen where antibody production begins. The bursa of Fabricius also facilitates gene conversion and somatic mutations, leading to increased antibody diversity (17). IgY antibodies are transferred from the maternal serum to the yolk during egg formation through a specific receptor on the surface of the yolk sac membrane (FcRY). However, FcRY is classified as an ortholog of the mammalian phospholipase A2 rather than mammalian FcRn (neonatal Fc receptor) or major histocompatibilitycomplex (MHC). IgY binding and endocytosis occur under acidic conditions, mimicking uptake of IgG by FcRn. This selective transfer of IgY provides passive immunity to the developing embryo (23, 24).

Oral administration of active compounds for the treatment and/or prevention, which is a well-accepted and safe mode, requires the delivery of intact particles. The stability of IgYs in the gastrointestinal tract was the subject of current research (25–27). Carlander et al. reported that, although a large part of IgYs is proteolytically digested by pepsin and trypsin to the Fab, Fab′_2_, and Fc fragments, their specificity and antigen binding ability are not lost (25). On the contrary, Wang et al. reported that a significant fraction of the specific activity of IgYs was lost shortly after exposure to the gizzard content in ex vivo assays performed with the model of chicken gastrointestinal tract. Furthermore, they also reported partial degradation in the small intestine (27). Lee et al. revealed that the encapsulation of hen antibodies effectively improves their stability and activity in the mice’s gastrointestinal tract (26). Controlled release of IgY from the protective layers such as methacrylic acid copolymer (28), chitosan-alginate (29), polypeptide microencapsulation (30), or the use of microgels formulated with carrageenan and alginate (31) has also been the subject of studies recently.

## Efficient production of antibodies in accordance with ethical requirements

Specific IgY antibodies can be non-invasively isolated from egg yolks of immunized hens without resorting to bleeding animals, which meets at least one requirement of the 3R principle: reduction, replacement, and refinement (32). The replacement of rodents with hens could reduce the number of animals necessary to obtain a certain amount of antibodies, which is an undoubted economic and ethical advantage. A hen lays around 300 eggs during her lifetime, which translates into the production of 18.25 grams of IgY antibodies (19, 33). The immunization strategy and housing conditions of hens were established in 1996 during the course of workshops organized by the European Centre for the Validation of Alternative Methods (ECVAM). **Table 1** presents details concerning the optimal conditions for obtaining chicken antibodies (34). Although the ECVAM recommendations for the production of IgYs have been in place for some time, there are no updates or new general guidelines issued. The information on the optimal conditions for breeding laying hens can be found in individual studies, such as those on the impact of cage size on animal welfare (35, 36) or alternatives to the Freund’s complete adjuvant (FCA) (37). Cage systems are preferred for breeding hens over cage-free systems due to lower risks of infectious disease transmission and severe feather pecking. It is also easier to divide the animals into study groups and keep them until the end of immunization (38). For IgYs production, laying hens are the organism of choice. Less frequently, ducks, geese, or ostriches are used (32). Noteworthy, the care of the animal welfare as in the case of IgYs application instead of mammalian immunoglobulins received the approval of the Swiss government in 1999 (39).

**Table 1:** Recommendations concerning optimal conditions for IgYs production based on (34).

ECVAM Recommendations	
Adjuvant	Freund’s incomplete adjuvant, Specol, lipopeptide Pam_3_-Cys-Ser-[Lys]_4_
Antigen	10 ng to 1 mg; optimal 10–100 μg
Injection site	Intramuscularly (breast muscle), subcutaneously (neck); preferably two injection sites
Injection volume	Up to 1 ml
Frequency of boosters	Boosters every 4 to 8 weeks; two to three times
Breeding for scientific purposes	Cages measuring 128 × 65 × 80 cm; two individuals in a cage
Age	At least 7 weeks

The total amount of IgY antibodies in a single egg yolk varies between species and oscillates between 50 and 150 mg of which 2%–10% display a desired antigenic specificity (34, 65). The production of specific IgY antibodies, much as the production of mammalian antibodies, is influenced by the molecular weight and dose of the antigen, the type of adjuvant used, the site and frequency of the administration of the antigen, and the animal’s condition (66). The enhancement of the post-vaccination response of the animal’s immune system to the administered antigen is achieved by the simultaneous administration of an adjuvant that non-specifically stimulates B lymphocytes. Among more than 100 described adjuvants, FCA, which is an emulsion paraffin oil with suspended inactivated *Mycobacterium tuberculosis*, is frequently used, especially for scientific purposes (67). Because of the possible side effects caused by Freund’s adjuvant (inflammation, tissue damage, and pain), research is being conducted to develop an adjuvant that can replace FCA. Promising candidates include Montanide adjuvant, which exhibits less severe side effects, and water soluble λ-carrageenan, which is less viscous than Freund adjuvants (37, 68).

Several methods concerning the isolation of IgY [e.g., caprylic acid–based protocol (69), PEG-based precipitation (33, 70), and dilution methods with pectin, followed by ammonium sulphate precipitation (71); for more detailed review of purification methods, see (32, 72)] have been developed, and the method of choice is most often dictated by further use, yield, and purity of isolated antibodies. Because of the demand for large amounts of antibodies, it was necessary to develop efficient, cost-effective, and scalable IgY isolation methods. The industrial-scale challenge is the separation of water-soluble proteins from the hydrophobic particles, particularly lipids. According to Bizanov, one of the most easily applied methods of native IgY isolation is water dilution followed by filtration (72). Recently described methodologies include the formation of aqueous biphasic systems from water-soluble proteins, followed by centrifugal partition chromatography (73). One limitation of the IgY purification is caused by the structural differences located within the Fc region, which results in their inability to interact with bacterial proteins A and G commonly used for purification of mammalian immunoglobulins. However, it was found possible to purify IgY with the application of protein M (transmembrane protein from human mycoplasma) (74). The current approach to food (supplement) production emphasizes the use of ecological methods, and, in the case of IgY products, there is no need for them to be highly purified for such purposes. Water dilution and desalting precipitation methods are considered the most cost-effective and eco-friendly technologies. These methods produce biodegradable waste that can be easily managed without significant harm to the environment, provided that proper waste management protocols are followed (75). In addition, post-production residues as egg whites and shells can be effectively used (e.g., for fodder production). In particular, eggshell waste exploitation is attracting more and more attention including the production of antibacterial materials, nanoparticles, adsorbents, and biomass (76, 77). When considering IgY antibodies as therapeutic agents, it is important to emphasize the variety of available preparations and the level of IgY purification required for a specified product. Different compositions may vary in terms of constituents of a hen’s egg, which are detailed and comprehensively described by Cherian (78). It is worth noting that various molecules within the egg may serve as allergens for the organisms exposed to IgY preparations. Many of these molecules are present in the white of the hen’s egg, such as ovoalbumin (50% of egg white proteins), ovomucoid [10% of egg white proteins; most allergenic egg white protein (79)], and lysozyme. Therefore, preparations isolated solely from hen’s egg yolks might be devoid of them. There are also allergenic proteins in egg yolks such as apovitellenis, phospvitins, and livetins (80).

There are several approaches to developing monoclonal IgY (mIgY) including hybrydoma technology and display technology (phage display, yeast surface display, and ribosomal display). mIgYs combines benefits resulting from the application of IgY (possibility to obtained antibodies specific to highly conserved mammalian proteins) and monoclonal immunoglobulins (increased repeatability between batches) (81). Hybridoma technology used for the production of the mIgYs [pioneering research developed by Nishinaka et al. (82)] proves to be difficult, more complex, and less efficient than the application of the mouse system. Mismatching and mis-recombining between the fusion partners, cultivation conditions such as temperature, and the final purification of the products (lack of reactivity with protein A or G) are among the problems encountered during the production of mIgY with hybridoma technique. Thus, mIgY production by hybridoma technology is complex and time-consuming compared with the production of murine monoclonal antibodies (mAbs) (83). As a substitute to the hybridoma, chicken lymphoma DT40 cell line that produces IgM-type antibodies could be employed. After treating the cells with trichostatin A, they are more susceptible to diversification at the immunoglobulin locus (84, 85). Alternatively, there are genetic engineering technologies and different selection methods (phage display and yeast surface display) that could be utilized to obtain mIgY preparations [chimeric chicken/human Fab (86) and scFv (44, 87–89)] for diagnostic/detection use. Nevertheless, the monoclonal IgY production methods are, at the moment, complex and expensive, and, therefore, mIgY are not competitive with polyclonal IgY when considering supplements/food additives market. Because of high immunogenicity of hen antibodies for mammals, their clinical applications in humans are limited. With the use of genetic engineering techniques, however, recombinant equivalents could be used (83). Yakhkeshi et al. have analyzed the current state of the IgY market, with a focus on both polyclonal and monoclonal IgYs (90).

## Mycobacterium tuberculosis

The 2020 WHO estimate says that TB, a major bacterial cause of death, afflicts 127 people per 100,000 (Global tuberculosis report, 2021). TB is caused by *Mycobacterium tuberculosis* that not only mainly infects lungs but also affects the kidneys (91), spine (92), and brain (93). This infection is especially dangerous for immunocompromised individuals suffering from HIV (94), cancer (95), and diabetes (96). *M. tuberculosis* is conducive to developing drug resistance and reactivating latent bacteria. Current treatment is based on first-line anti-TB drugs, such as rifampicin and isoniazid, and second-line drugs that are more sophisticated and expensive and display high toxicity (e.g., fluoroquinolones and aminoglycosides) (97). In some countries, Bacille Calmette-Guérin (BCG) vaccine against TB is used as prevention. Vaccine efficacy is maintained by a constant development of novel effective vaccine candidates (98, 99).

Sudjarwo et al. summed up the studies focused on the production of IgYs specific to *M. tuberculosis* antigens. Formaldehyde-inactivated *M. tuberculosis* (ATCC H37 Rv) was used for Lohmann laying hen immunization (four injections every 2 weeks of 80 μg of an antigen) followed by IgY isolation *via* the polyethylene glycol (PEG) extraction/ammonium precipitation method (40). The potency of generated IgY antibodies against *M. tuberculosis* antigens was evaluated with 3−(4,5−dimethylthiazol−2−yl)−2,5−diphenyl tetrazolium bromide–based cell viability assay with rat Peripheral Blood MononuclearCells (PBMCs) together with the measurement of Interleukin-2(IL-2) and Interferon-γ (INF-γ) levels in PBMC supernatants. The results have indicated that *M. tuberculosis* antigen–specific IgYs increased the production of IL-2 and INF-γ in a dose-dependent manner (41).

The patented approach to the use of specific IgY in the treatment of *M. tuberculosis* infections, especially caused by antibiotic-resistant strains, is a personalized therapy with the vaccine prepared individually from the strain isolated from the patient. The derived antibody was intended to be used orally alongside any other therapy (100). For a summary of IgY studies, see the **Table 2**.

**Table 2:** Production and application of IgYs specific to *Mycobacterium tuberculosis*.

Immunogen	Activity/Properties	Reference
Formaldehyde-fixed strains of *M. tuberculosis* (ATCC H37 Rv)	*In vitro*; immunoblotting, ELISA; IgY antibodies specific to *M. tuberculosis*	Sudjarwo et al. (40)
No specified data	*In vitro*; PBMC viability assay, ELISA; IgY anti-*M. tuberculosis* increased IL-2 and INF-γ production in rat PBMC; no specified data concerning adjuvant	Sudjarwo et al. (41)

If not specified in the Activity/Properties column, then FCA/Freund's Incomplete Adjuvant (FIA) was used as an adjuvant.

## Acinetobacter baumannii

The WHO has classified *Acinetobacter baumannii* as a pathogen that urgently requires the development of new antibiotics. Because only few effective antibiotics toward *A. baumannii*, together with *Pseudomonas aeruginosa* and *Enterobacteriaceae*, are currently available, it has been classified as the critical priority. *A. baumannii* is associated with the hospital-acquired infections of the urinary tract, skin, soft tissues, and bones (101). A multidrug-resistant *A. baumannii* is especially dangerous for patients in intensive care units where mortality ranges from 10% to 43% (102). The resistance mechanisms of *A. baumannii* include production of enzymes able to modify aminoglycosides, expression of multidrug efflux pumps, and permeability defects that make certain strains highly resistant to routinely used antibiotics (103). Among *A. baumannii* strains, some are considered extremely drug-resistant (XDR-Ab) or pan-drug-resistant (PDR-Ab): They exhibit resistance to a majority or nearly all antimicrobials tested routinely (104, 105). Carbapenems (including imipenem, meropenem, and doripenem), inhibitors of β-lactamase (sulbactam), minocycline (broad-spectrum tetracycline antibiotic), tigecycline (glycylcycline class antibiotic), and polymyxins (polycationic peptide antibiotics) represent a prospective treatment option against *A. baumannii* infections (106).

Passive immunization with specific antibodies to overcome *A. baumannii* infections is an alternative. Nielsen et al. described the therapeutic effect of mammalian monoclonal antibodies (Mab C8 and Mab 65; derived after immunization with sublethal inocula of virulent XDR-Ab clinical isolates) in lethal bacteremic sepsis and aspiration pneumonia in murine models of infection. They proved a synergistic effect of Mabs when administrated with antibiotics (107, 108). Yeganeh et al. used an immunogenic peptide derived from *A. baumannii* OmpA protein for the immunization, which led to the selection of 3F10-C9 Mabs exhibiting the potential for further evaluation as a novel therapeutic approach (109). Shi et al. described the potency of IgYs obtained after hen immunization with formaldehyde-inactivated PDR-Ab strain. IgYs were able to inhibit the growth of PDR-Ab *in vitro* (at a concentration of 20 mg/ml, they significantly inhibited the growth of PDR-Ab within 24 h) and reduced the mortality of PDR-Ab–infected mice (IgYs administrated intraperitoneally at 250 mg/kg). The results showed a decreased level of serum cytokines Tumor necrosis factor-α [(TNF-α) and IL-1β] and reduced inflammation of lung tissue (42). Different studies reported a protective effect of IgYs specific to OmpA and Omp34 of *A. baumannii* in the murine pneumonia model of infection. The highest specificity exhibited antibodies raised against a combination of recombinant OmpA and Omp34 proteins, whereas IgYs antibodies raised against inactivated *A. baumannii* cells displayed the lowest specificity (43, 110). The analysis of Omp34 structure revealed an immunodominant loop (L3) exposed in the native form of the protein. The construct composed of five connected L3 loops (Omp34L3X5) used for immunization led to antibodies able to recognize not only the immunogen but also native Omp34 as well as *A. baumannii* cells. The antibacterial potency of specific IgYs was evaluated in a murine model of *A. baumannii*–induced pneumonia. In the comparison studies (intranasal administration of 20 μl of IgYs at 2 mg/ml) between anti-Omp34 activity and anti-Omp34L3X5, a higher potency of action was observed for anti-Omp34 IgYs (survival rate of 100% *vs.* 83%) (45). Another research on IgYs specific to *A. baumannii* application was reported by Ranjbar et al. The IgYs specific to biofilm-associated (Bap) protein (amino acids 706–1,061) of *A. baumannii* prevented the biofilm formation (concentration of specific IgYs form 50 to 200 μg/ml) and displayed the protective effect in mice infected with pathogen [40 μg of IgY antibodies/20 μl of Phosphate Buffered Saline (PBS)] (46).

Two inactivated strains of *A. baumannii*, standard and multidrug resistant, were used as antigens to produce IgY in the invention presented by Zhen et al. The *in vitro* analysis revealed the significant inhibitory potency of both groups of specific IgY against the *A. baumannii* even at the lowest tested concentration (5 mg/ml), whereas the inhibition of the second strain showed a more dose-dependent characteristic. The activity of the antibodies was also confirmed in the mouse pneumonia model established with the multidrug-resistant strain of *A. baumannii*, where the 4-day mortality rate was the same (20%) as in the group receiving cefoperazone and sulbactam as treatment, and much lower than in the negative control group (90%). Alongside with biological testing, several possible medical formulations of patented IgY are presented including tablets, capsule/microcapsule, ointment, and injection (47). For a summary of IgY studies, see the **Table 3**.

**Table 3:** Production and application of IgY antibodies specific to *A. baumannii*.

Immunogen	Activity/Properties	Reference
Formaldehyde-fixed strains of *A. baumannii* (ATCC BAA1605 and clinically isolated strain)	Murine model; intraperitoneal injection lowered mortality of infected mice, decreased TNF-α and IL-1β, reduced inflammation of the lung tissue	Shi et al. (42)
Formaldehyde-fixed *A. baumannii*, recombinant OmpA and recombinant Omp34 proteins	Murine pneumonia model; protection against *A. baumannii* infection (with OmpA as a most potent antigen)	Jahangiri et al. (43)
Formaldehyde-fixed *A. baumannii*, recombinant OmpA and recombinant Omp34 proteins	Murine pneumonia model; intranasal delivery therapeutic effect in the murine pneumonia model	Jahangiri et al. (44)
Recombinant Omp34L3X5 and recombinant Omp34 proteins	Murine model; intranasal delivery protection against *A. baumannii* infection	Maghaddam et al. (45)
Bap recombinant protein	Murine model; intranasal delivery anti-Bap IgY reduces mortality caused by pneumonic infection when administered prophylactically	Ranjbar et al. (46)
Inactivated standard and multidrug-resistant *A. baumannii* cells	Murine model; IgY of 20 mg/ml (150 μl per 10 g of body weight) 24 h before inoculation and 250 μl per 10 g of body weight daily for 5 days significant mortality reduction	Zhen et al. (47)

If not specified in the Activity/Properties column, then FCA/FIA was used as an adjuvant.

## Pseudomonas aeruginosa

Another microorganism that develops various mechanisms against antibiotic therapy is gram-negative *Pseudomonas aeruginosa*. It is a common cause of nosocomial infections (estimated prevalence amongst all healthcare-associated infections is 7.1%–7.3%) and is especially dangerous to immunocompromised individuals and patients with lung disease including autosomal recessive acquisition of mutations in cystic fibrosis (CF) (111). In patients with CF, the treatment of *P. aeruginosa* needs to be initiated at a very early stage of the infection, for example, by inhalation with tobramycin or aztreonam, compounded by antibiotic therapy including cefepime or meropenem. Patients with CF treated with antibiotics need to be monitored for nephrotoxicity (111). Kollberg et al. proposed an alternative, which is immunotherapy with antipseudomonal IgYs, which was supposed to prevent or delay the infection of the lower airways and the resultant colonization of the lungs by *P. aeruginosa* (49). Antipseudomonal IgY decreases the level of adhesion to the oropharynx. Oral administration of IgYs entails no risk of having resistance to IgY develop because antibodies are not absorbed into blood (112).

One of the challenges was to determine the mechanism of antibacterial action of *Pseudomonas*-specific IgY antibodies. The antigenic specificity of the IgYs provided additional information. Nilsson et al. in their *in vitro* studies revealed that the IgY-specific *P. aeruginosa* is reactive against flagellin (types a and b) that is responsible for the motility and chemotaxis of the bacteria. Thus, chicken antibodies are able to reduce invasion because they reduce adherence, mobility, and inflammatory response (48). Thomsen et al. revealed that IgY-specific to *P. aeruginosa* opsonize the pathogen from different strains (including clinical isolates) *in vitro*. As a consequence, the innate activity mechanism is mobilized through polymorphonuclear neutrophils. Postulated mechanism does not include conventional F_c_ receptor–dependent opsonization. Instead, the probable mechanism assumes phagocytosis mediated through alterations in physio-chemical conditions of the bacteria (50).

Promising results concerning antipseudomonal non-antibiotic activity of IgYs prompted further studies on animal models. The murine pneumonia model was used for the investigation regarding prophylactic therapy for *P. aeruginosa*. Specific antibodies delivered to Balb/c mice facilitate the bacterial clearance and decrease inflammation (52). Thomsen et al. evaluated the effectiveness of the combination: azithromycin that is beneficial for the immunomodulatory mechanism and specific IgYs against *P. aeruginosa* that were able to enhance opsonization. Balb/c mice were subjected to the antipseudomonal IgYs while one experimental group was pretreated with azithromycin (20 mg/kg). Specific IgY significantly reduced the infection. In addition, azithromycin combined with IgY enhances the reduction in pulmonary inflammation (53). In the *in vitro* research conducted by Sanches et al., IgY specific to *P. aeruginosa* (0.625–2.5 mg/ml) revealed synergistic antimicrobial action with beta-lactams: ceftazidime, imipenem, and meropenem (51). Anti-*Pseudomonas* IgY were also evaluated against *P. aeruginosa*–resistive urinary tract infections. Research conducted with the use of the murine model of urinary tract infection revealed that co-administration of specific IgY (0.2 mg per mouse, described previously by Thomsen et al. (50)) simultaneous with mice infection decreases bacteriuria. In addition, both control (unspecific) and specific (antipseudomonal) IgYs (0.2 mg per mouse) exhibited a prophylactic effect when administered intravesically before infection with bacterial solution (54).

Otterbeck et al. conducted research into the effect of specific IgY antibodies (immunization with inactivated *P. aeruginosa*) on the colonization lower airways in large animal models, anesthetized and mechanically ventilated porcine models. The animals were nebulized with *P. aeruginosa* or *P. aeruginosa* supplemented with anti–*P. aeruginosa* IgYs (50 mg). Specific IgY antibodies decreased the bacterial colonization of the respiratory tract for 12 h. After that time, the therapeutic effect wore off, and the authors noticed an increase in bacteria (55). In other experiments conducted with antipseudomonal IgYs and animal models of ventilator-associated pneumonia, Otterbeck et al. did not register any significant reduction in the bronchoalveolar lavage concentration of *P. aeruginosa*. In these trials, antibodies were administered intravenously (100 mg per animal) (57) or injected bronchially (100 mg per animal) (56). The results contradicted those obtained in *in vitro* assays, in the pneumonia mice model, and in patients with CF; however, it is difficult to compare because different administration routes were applied (50, 112). Similar inhibition results—lack of protection against *P. aeruginosa* despite good reactivity—were obtained by Zamani et al. with IgYs specific to whole pathogen and toward pilQ-pilA-DSL chimeric protein. Antibodies (10 mg/kg) were administered intravenously to rabbits 24 h before *P. aeruginosa* infection (58).

Antipseudomonal IgYs were the subject of two clinical trials. The efficacy of anti–*P. aeruginosa* IgY as a preventive agent for patients with CF was analyzed in Sweden (Cystic fibrosis center, Children´s University Hospital, Uppsala, Sweden) from 2008 to 2016 [*Post Marketing Study of Anti-pseudomonas IgY in Prevention of Recurrence of Pseudomonas Aeruginosa Infections in Cystic Fibrosis (CF) Patients*. ClinicalTrials.gov identifier: NCT00633191. Updated 1 September 2016. Accessed 30 January 2023]. Antibodies were obtained after immunization with *P. aeruginosa* and purified with the water dilution method. Fourteen participants with CF infected occasionally with *P. aeruginosa* were subjected to preliminary antibiotics therapy followed by gargling with specific IgYs solution. As a result, it was observed that patients who received this oral immunotherapy became infected later than those not treated with the IgYs. In another trial conducted from 2011 to 2017 [*Phase III Study to Evaluate Clinical Efficacy and Safety of Avian Polyclonal Anti-Pseudomonas Antibodies (IgY) in Prevention of Recurrence of Pseudomonas Aeruginosa Infection in Cystic Fibrosis Patients*. ClinicalTrials.gov identifier: NCT01455675. Updated 6 July 2017. Accessed 30 January 2023], the number of patients was increased to 164, and they were divided into the placebo (70 ml of gargling solution without IgY) and IgY (70 ml of gargling solution; 50 mg of anti–*P. aeruginosa* IgY) group (double-blind). The results show that detection of anti–*P. aeruginosa* antibodies in the oral cavity was possible for 8–24 h after gargling. The patients’ condition monitored by the CRP) level revealed no active infection during the course of testing. The experiment also sought to answer the question whether the serum of treated patents contains antibodies specific to antipseudomonal IgYs that could reduce effectivity of the therapy and induce allergic reactions. According to final report, the level of anti-IgY antibodies was low (even lower than reported for therapies conducted with mammalian antibodies). In this trial, the efficacy of antipseudomonal IgY antibodies was not possible to determine because of the unexpected low amount of events in the placebo group (59). For a summary of IgY studies, see the **Table 4**.

**Table 4:** Studies concerning the production and application of IgY antibodies specific to *P. aeruginosa*.

Immunogen	Activity/Properties	Reference
Mixture of formaldehyde-fixed strains *P. aeruginosa*: PAO1, PAO3, PAO5, PAO6, PAO9, and PAO11	*In vitro*; immunoblotting; antibodies specific to flagelin a and b	Nilsson et al. (48)
Different O-antigen strains of *P. aeruginosa*: O1, O3, O5, O6, O9, and O11 (purchased from ImmunSystem I.M.S.)	*In vitro*; respiratory burst assay; polymorphonuclear neutrophil–mediated bacterial killing IgY-mediated enhancement of PMN phagocytosis, promotion of the opsonization; adjuvant manufactured according to the standard procedure described by Kollberg et al. (49)	Thomsen et al. (50)
Formaldehyde-fixed strains *P. aeruginosa* Pa48_spm-1+_ and Pa23_vim-2+_ isolates	*In vitro*; growth inhibition assay; specific IgY inhibitory activity, synergistic action of specific IgYs and selected beta-lactams	Sanchez et al. (51)
Different O-antigen strains of *P. aeruginosa*: O1, O3, O5, O6, O9, and O11 (purchased form ImmunSystem I.M.S.)	Acute pneumonia mice model; passive immunization IgY therapy promote bacterial clearance and limit inflammation in *P. aeruginosa* lung infection; adjuvant manufactured according to the standard procedure described by Kollberg et al. (49)	Thomsen et al. (52)
Different O-antigen strains of *P. aeruginosa*: O1, O3, O5, O6, O9, and O11 (purchased form ImmunSystem I.M.S.)	Murine lung infection model; passive immunization combinatory treatment with azithromycin enhance pulmonary elimination of *P. aeruginosa*; no specified data concerning adjuvant	Thomsen et al. (53)
Different O-antigen strains of *P. aeruginosa*: O1, O3, O5, O6, O9, and O11 (purchased from IgY Lab System)	Murine urinary tract infection model; intravesical passive immunization reduction of the *P. aeruginosa* colonization in bladder	Schwartz et al. (54)
inactivated *P. aeruginosa*	Porcine model, mechanically ventilated; transient reduction of the *P. aeruginosa* colonization in the airways after nebulization	Otterbeck et al. (55)
Inactivated *P. aeruginosa*	Porcine model, mechanically ventilated; bronchially instilled specific IgYs did not reduce the concentration of *P. aeruginosa* in the lower airways	Otterbeck et al. (56)
Inactivated *P. aeruginosa*	Porcine model, mechanically ventilated; intravenous specific IgYs did not reduce the concentration of *P. aeruginosa* in the lower airways	Otterbeck et al. (57)
Inactivated *P. aeruginosa*, chimeric protein pilQ-pilA-DSL	Rabbit model of sepsis; intravenous protection from *P. aeruginosa* infection was not effective in rabbit sepsis model; Montanide ISA 70 V G adjuvant	Zamani et al. (58)
Mixture of two formaldehyde-fixed strains *P. aeruginosa*: PAO1 (ATCC 15692) and Habs1 (ATCC 33348)	Phase I clinical trial; gargling prevention against chronic *P. aeruginosa* infection	Kollberg et al. (49)
strains of *P. aeruginosa*	Phase III clinical trial; gargling as an effect of the trial efficacy could not be described; no specified data concerning adjuvant	(59)
Mannose-sensitive *P. aeruginosa* strain antigen	Rat animal model; 2 ml (1 mg/ml) every 12 h, three doses bacteriostatic effect comparable with ceftazidime; no specified data concerning adjuvant	Qi et al. (60)
Mannose-sensitive *P. aeruginosa* strain antigen	Mice model; 2 ml of IgY a day for 2 weeks with or without cisplatin (1 mg/kg) enhanced inhibition in tumor growth in combination therapy group; no specified data concerning adjuvant	Qi et al. (60)
Mannose-sensitive *P. aeruginosa* strain antigen	Human; orally supplemented every other day improved function of the immune system; no specified data concerning adjuvant	Qi et al. (60)
Ultrasonically disintegrated *P. aeruginosa*	*In vitro*; Fab’ IgY fragment of 5, 10, and 20 mg/ml; incubation up to 36 h results in growth inhibition	Zhang et al. (61)
*P. aeruginosa*	Rat animal model; 2 g of IgY/day for 3 days + 1 dose 8 h after inoculation; reduction in bacteria and endotoxin level and pathological changes in liver or ileum; no specified data concerning adjuvant	Rongjian et al. (62)
Recombinant type A and B Flagellin	Murine model; burn wound infection, 500 μg of IgY mixed with lethal dose of *P. aeruginosa*, additional IgY doses 2, 24, 48, and 72 h later; 100% animals survival rate for anti–flagellin B IgY; acute pneumonia model, bacteria mixed with 500 μg of IgY, additional doses (250 μg) 2 and 24 h later, both tested groups 100% survival rate; adjuvant: ISA70VG + CpG-ODN.	Ahmadi et al. (63)
Recombinant outer membrane protein F	Murine model; burn wound infection, one dose of 0.1 or 10 mg of IgY preincubated with *P. aeruginosa;* 1 mg of IgY preincubated with pathogen and 0.5 mg of IgY 12 h after infection; prophylactic treatment with 0.5 mg of IgY 2 h prior to infection and 0.5 mg of IgY 12 and 24 h after infection, increased survival rate up to 87.5%.	Norouzi et al. (64)

If not specified in the Activity/Properties column, then FCA/FIA was used as an adjuvant.

An antimicrobial treatment in the case of cancer patients can be beneficial as an adjuvant therapy (113). IgY antibodies specific to the mannose-sensitive strain of *Pseudomonas aeruginosa* were prepared and tested as immune adjuvant therapy. In the case of *P. aeruginosa* infection tested in a rat model, the antimicrobial activity of the antibodies was similar to those observed in the ceftazidime treatment group. The therapeutic value of anti–*P. aeruginosa* was confirmed *in vivo* in a lung cancer model with improved inhibition of tumor growth in the group receiving cisplatin and IgY combination treatment compared to cisplatin treatment alone. A clinical study on a group of patients with metastatic lung cancer receiving chemotherapy showed improvement in cellular and humoral immunity (based on blood analysis) after the introduction of IgY antibody as a supplemented adjuvant treatment (60). The protective effect of anti–*P. aeruginosa* IgY was also described in the invention of Rongjian and co-workers (62). The portal hypertension model was established in rats with the treated group receiving yolk immunoglobulins before (for 3 days) and after inoculation with *P. aeruginosa* (one dose after 8 h). After the model was completed, the analysis of collected specimens revealed the reduction in mesenteric lymphadenitis and lower level of portal blood and intestinal bacteria, and endotoxins in blood. The pathological changes in the liver and ileum were also reduced in the group treated with specific IgY compared to the untreated portal hypertension group. The same invention describes the effect of IgY administration on intestinal infection in severely burned rats model with similar results, confirming not only the antibacterial but also preventive value of IgY in the case of secondary infections, e.g., in sepsis or other trauma.

The IgY antibodies produced with whole-cell lysate were employed to create an anti–*P. aeruginosa* Fab’ fragment (61). The Fab’ fragment was yielded by the pepsin digestion approach (114). In the invention, the authors presented the description of fragment activity with enzyme-linked immunoassay (ELISA) and *in vitro* the bacteria growth inhibition test (61).

Egg yolk antibodies were also generated after immunization with *P. aeruginosa* recombinant flagellin A and B proteins. The *in vitro* reduction of bacterial strain motility, biofilm formation, and cell invasion confirmed the activity of specific IgY. Experiments were performed on PAK and PAO1 (type A and B flagellin–specific strains) and R5 (multidrug-resistant) strains showing not only strain-specific but also cross-reactive potential of tested immunoglobulins. Most differences between antibodies specific to type A and B flagellin were observed in opsonophagocytic assay and in the *in vivo* burn-wound infection mouse model. Both experiments showed the superiority of anti–flagellin B IgY with 100% animals survival rate after infection with PAK and PAO1 strains (40% in R5 group and 0% in other infected groups) and with an up to 94.7% reduction in cells’ survival percentage (PAK strain) in opsonophagocytic assay. The possible explanation of these differences is hypervariability of flagellin A central domain observed between strains. Nevertheless, antibodies of both specificities provided 100% protection against *P. aeruginosa* lethal pneumonia when tested in mice, compared with 0% in groups not receiving IgY and 20%–40% in control IgY–treated groups (63). The neutralization of *P. aeruginosa* wound infections in mice with IgY specific toward recombinant outer membrane protein (OMP) F was tested by Norouzi and co-workers. The survival rate was 25% when specific antibody was only preincubated with bacteria (dose of 0.1 and 10 mg) and up to 87.5% when first dose of IgY was given 2 h before infection (1 mg) and additional treatment after 12 and 24 h (0.5 mg) (64).

## Gastrointestinal infections

The easiest and, therefore, most common portals of entry for pathogens, including bacteria, are the respiratory and gastrointestinal tracts. Mucosal surfaces as defensive barriers prevent entry and colonization of pathogenic microorganisms while ensuring the possibility of the colonization by symbiont microbiota. Still, the presence of vulnerable mucosal surfaces and isolated, steady-state, moist environment of those tracts could be a convenient starting point for further invasion. On the other hand, the most convenient application of IgY antibodies is *via* the oral or inhaled route allowing the preventive/therapeutic IgY action based on passive immunity at the site of the infection. Because of the substantial advantages of IgY technology, including ethical and economical aspects, antibacterial IgY antibodies and their preparations for human and veterinarian use are the subject of not only scientific publications but also patent applications. In this context, the often underestimated superiority of IgY antibodies compared with mammalian IgG are that eggs are generally non-controversial and well-tolerated food product and IgY antibodies can be isolated in a relatively low-impact and easy manner.

## Helicobacter pylori

*H. pylori* is one of the most extensively studied bacterial targets for specific IgY production. *H. pylori* infections are associated with several gastrointestinal diseases, including peptic ulcer disease and gastric adenocarcinoma. Decreasing prevalence of *H. pylori* infections in recent years is mainly due to the lower incidence rate observed in developed countries, but not globally, with a prevalence of 37% in the northern American region and nearly 80% in Africa (115). The decline in prevalence is mainly attributed not only to a better healthcare system and hygiene level but also to the general epidemiological awareness. The growing antibiotic resistance of *H. pylori* points to the urgent need for the development of new therapeutic approaches (116, 117). The analyzed alternatives to the standard antibiotic-based treatment include probiotic administration (as adjuvant) (118), vaccines (119), natural and synthetic non-antibiotic compounds, or phage therapy (120). One of the possible alternatives that work in the form of passive immunization is the specific IgY antibodies, and multiple possible approaches to the generation of specific immunoglobulin Y and the final product formula and/or its composition are extensively described in the scientific literature and patents.

### H. pylori whole cell

In the case of anti-microbial antibodies generation, the most straightforward idea for the immunogen design is the utilization of inactivated, whole-cell bacteria. In many cases, immunogen preparation is less expensive and easier, with polyclonal antibodies showing reactivity toward multiple bacterial antigens. Still, polyclonal immunoglobulins generated by such an approach can be the source of cross-reactivity mainly with other microorganisms, including bacteria present in healthy human intestinal microflora. In the study described by Shin and co-workers, specific IgY antibodies were able to inhibit *H. pylori* growth by 90% and urease activity by 84.5% when used at a concentration of 10 mg. *In vivo* studies show increased infiltration of lymphocytes and neutrophils, and, consequently, a reduction in *H. pylori*–induced gastric mucosal injury in the IgY-treated gerbil model. Treatment, in both *in vitro* and *in vivo* studies, was applied with a dose of 1 and 10 mg but with the use of non-affinity purified material, and, therefore, the amount of antigen-specific antibodies was at least tenfold lower. Furthermore, while considering therapeutic use in the form of an orally administered product, the authors showed not only the pH stability of IgYs but also the thermal stability. According to the study, the antibodies can be treated for at least 10 min at 60°C with an activity loss of only 20% (121). The thermal stability of the IgY antibodies was also confirmed at a temperature of 65°C for 30 min, which is one of the standard pasteurization procedures (122).

Even more promising results were yielded by the experiments performed by Yang et al. (123). The activity of anti–*H. pylori* IgY antibodies tested on mice showed dose-dependent potential in eradicating the pathogen from the stomach after intragastric *H. pylori* inoculation. The efficacy of the treatment was confirmed by rapid test for the urease activity (Campylobacter-like organism test, CLO test), which showed 87.5% efficacy (*H. pylori* elimination from the stomach) in groups receiving a dose of 200 or 500 mg/kg. The inflammatory lesions were minimized with higher doses of antibodies. The treatment was more effective than in the previous study (121), but the study was conducted on different animal species using different treatment scheme, and antibodies were generated using formaldehyde-deactivated whole cells *vs*. cell lysate (123). Wang et al. presented interesting results from their study on specific IgY antibodies that were tested alone and in combination with sucralfate (124). Antigen-specific antibodies showed similar antibacterial activity and inhibitory potential toward urease activity when tested alone and in combination, but the potency of action was improved in *in vivo* studies for antibodies supplemented with sucralfate. Combination therapy resulted in the highest infection clearance rate (83.3%), which is superior to the effect obtained after clarithromycin and omeprazole therapy (66.7%) that is used frequently as a dual therapy for the treatment of *H. pylori* infections. Importantly, the immunohistochemical and pathological examination of gastric tissue showed little or no changes in groups treated with specific IgY antibodies and specific IgYs in combination with sucralfate, which confirmed the protective effect of avian antibodies. The study also shows that the specific anti–*H. pylori* IgY has the ability to inhibit the growth of *H. pylori in vitro* but, at the same time, does not influence the growth of *Escherichia coli* and *Staphylococcus aureus*. The cross-strain reactivity of *H. pylori*–specific IgY antibodies was tested by Solhi et al. The inactivated bacteria cells used for hen immunization came from four *H. pylori* strains isolated from patients. The antibodies were capable not only of inhibiting the growth of the strain used originally for immunization but also of cross-reacting with other strains used in the experiment. Their potency was in the range of 48.98%–78.8% for same strain growth inhibition and 29.21%–86% for cross-strain analysis (125).

Because of differences between “classical” chicken IgYs and truncated duck IgY(ΔFc) antibodies, there is increasing interest in the generation and analysis of this type of avian immunoglobulins. The IgY(ΔFc) antibody generated with three strains of *H. pylori*, with a confirmed activity toward the antigen, together with its application as active ingredient of yogurt was patented (126).

There is a clearly visible trend toward patenting of *H. pylori–*specific IgY in a more processed form, with three main product categories: food supplements, pharmaceuticals, and functional food (mainly dairy products). In many of these inventions, the immunogen used for IgY production was *H. pylori* cells, but examples with the use of pure virulence factors (or mixtures thereof) especially urease are also present (described below). Moreover, as *H. pylori* belongs to digestive tract pathogens, in many IgY preparations, it was one of several antigens used for immunization. Such hyperimmune antibodies with their characteristic or commercially available products are presented in section IgY compositions and applications.

One of the examples of the application of *H. pylori*–specific IgYs, generated with the use of cell lysate, is the preparation of gastric floating tablets. This formulation allows an increase in retention time in the stomach, enhancing the therapeutic potency of the product (127, 128). This was confirmed in the *in vivo* study on mice inoculated with *H. pylori* and receiving floating preparations. The effect on these mice was compared to groups treated with the standard therapy and a non-floating preparation of specific IgY. The patented compositions were as active as the standard therapy and much more effective than non-floating preparation, based on the results of the rapid urease test (RUT) (127). Among patents describing functional food products enriched with anti–*H. pylori* antibodies, there are examples presenting functional products such as pudding or soy milk, together with the thermal stability assessment of IgY at different temperatures (129, 130). It has been also confirmed that toothpaste containing specific IgY antibodies has the ability to freshen breath effectively (131).

Considering the drawbacks of utilization of whole cells for the production of IgYs, several *H. pylori* proteins were successfully tested as immunogens. The experiments designed to identify most immunodominant proteins of the *H. pylori* cells lysate, when used for hens immunization, allowed the indication of several proteins as potent immunogens, including urease subunits, peroxiredoxin, chaperonin GroEL, flagellin A (FlagA), DNA starvation/stationary phase protection protein, heat-shock protein 60 (HSP60), and probable thiol peroxidase (132, 133). However, the pool of known *H. pylori* antigens includes several other generally recognized virulence factors such as vacuolating cytotoxin A (VacA), cytotoxin-associated gene A (CagA), catalase, outer inflammatory protein A (OipA), neutrophil-activating protein (NAP), blood group antigen–binding adhesin A (BabA), and sialic acid–binding adherence (SabA) (134, 135).

### H. pylori urease

One of the most studied virulence factors is urease, which has the highest expression level among *H. pylori* proteins and is crucial for its pathogenesis. The acidity of the stomach creates unfavorable living conditions for *H. pylori*. The enzymatic reaction catalyzed by urease results in the generation of ammonia and carbamate that increase pH. This is one of the most important features for *H. pylori* survival and pathogenesis, improving its nutrition and colonization conditions along with influencing the immune response of the host (135).

The high immunostimulatory potential of urease manifests by the presence of specific antibodies in infected patients’ sera (136). The IgY antibodies specific toward urease subunits alpha and beta are not only generated as a result of hen immunization with whole-cell lysate (132) or purified protein (137–139) but also selected immunodominant peptidyl epitopes used as Bovine Serum Albumin (BSA) conjugates (140). Interestingly, immunization with plasmid encoding urease B subunit resulted in specific IgY production in ducks (141, 142). As urease activity testing is the basis of *H. pylori* infection diagnostics, the potential of *H. pylori*–specific IgY antibodies to inhibit the enzyme activity was verified with antibodies generated with whole cells used as an antigen. The presence of anti-urease antibodies in such polyclonal/polyspecific IgYs isolated from hens yolk confirmed the high immunogenicity of urease (121, 124, 125). In addition, a more targeted approach where the antibodies were generated with peptide fragments selected on the basis of epitope mapping yielded specific IgYs capable of not only recognizing urease but also inhibiting its enzymatic activity with only 18.3 ± 7.6% of the control activity remaining (at the concentration of 10 mg/ml) (140). In the study reported by Suzuki et al. (138), the group of *H. pylori–*positive volunteers receiving the dietary supplement containing IgY antibody specific toward urease showed a decrease in the results of ^13^C-urea breath test (UBT) in 13 of the 17 research participants after 4-week of treatment. Further work by the same team presented the study on Mongolian gerbils where the effect of dietary anti-urease IgY administered with or without famotidine on the ongoing infection was compared with the prophylactic use when the antigen-specific antibodies supplemented with famotidine were used prior to *H. pylori* infection. In the first experiment, the animals received treatment (IgY, famotidine, and a combination of both) 10 weeks after being inoculated with *H. pylori*. None of the dietary supplements managed to eradicate the infection; however, the administration of IgY-famotidine allowed reducing mucosal myeloperoxidase (MPO) activity. In the second experiment, the IgY-famotidine diet was introduced a week before inoculation with *H. pylori*, and, 8 weeks later, no sign of colonization was observed (137). As the IgY stability and, therefore, antigen binding capacity is altered at lower pH of the stomach, the improved performance of IgY antibodies in the presence of famotidine can be attributed to the reduction in acidic conditions by this H_2_-receptor antagonist. In the experiment on mice infected with *H. pylori* and treated with IgY antibody specific toward recombinant UreC (recombinant urease subunit alpha) administered as a diet or solution, the therapeutic effect was observed 10 weeks after the treatment was withdrawn. Animals from IgY-treated groups showed a significant reduction in specific anti–*H. pylori* antibody titer in the sera, reduction in stomach tissue inflammation, and a decreased presence of pathogens in the mucosa layer. The results suggested a slight but not significant advantage of the IgY treatment in liquid form over administration as a food ingredient (139).

As the condition of the gastrointestinal tract can influence the IgY performance, combination therapy or different formulations/additives are often considered as methods to improve the outcome of treatment. *In vitro* and *in vivo* studies analyzed the synergy of action between anti-urease IgY and *Lactobacillus johnsonii* no. 1088 (LJ88) when used alone and in combination (143). LJ88 is a lactic acid bacteria isolate with unique anti–*H. pylori* activity (144, 145). The combined use of specific IgYs and LJ88 showed a significant synergistic effect on *H. pylori* growth. The effect was observed on five different strains including those that are clarithromycin resistant. For the *in vivo* study, the germ-free or human gut microbiota-associated mice model was used. The animals were fed with specific IgY or with living or killed LJ88 as a monotherapy and as a combination therapy. The most profound effect was observed for the combination therapy with anti-urease IgY and living LJ88 in the human gut microbiota-associated model. The reduction in the number of *H. pylori* in the stomach changed from 1.48-fold (IgY group) and 126-fold (LJ88 group) in the case of monotherapy into 1,259-fold when both dietary supplements were used (143). The evaluated synergistic combination is now used in IgYGate^®^ GastimunHP Plus food supplement recommended for patients during gastritis treatment (see section IgY market). Urease-specific IgY antibodies activity was also analyzed by Mony et al. in a mouse model. *H. pylori*–infected animals were fed twice a day with IgY doses from 50 to 500 mg/kg. The results clearly indicate that the antibodies can eradicate *H. pylori* infection in a dose-dependent manner (146).

One of the main drawbacks of IgY antibody’s oral application is its limited stability in low pH of the stomach contents (27, 121), even considering IgY’s superiority when compared with mammalian IgG (147). It is especially important when the target location of the activity of IgY is the stomach itself rather than the intestine, and gastro-resistant formulation cannot be used. Observations suggest that IgY used as a dietary supplement shows higher activity when administered in the right combination or formulation. Such characteristics of IgYs together with growing consumer interest in functional foods create vast opportunities for food and supplement market. One of the possible practical preparations of anti-urease IgY antibodies is an additive (1% *m*/*v*, 4.5 g of IgY/day) to the yogurt containing *Lactobacillus acidophilus* and *Bifidobacterium* spp. cultures (122). The resultant product was tested on humans. There was a significant reduction in *H. pylori* infection symptoms as measured by ^13^C-UBT over 4 weeks. The study showed that the specific IgY antibody remained relatively stable in the prepared drinking yogurt over 3 weeks with a 15% reduction in activity.

The patented invention of the pharmaceutical composition of IgY and famotidine as active ingredients describes the generation of IgY antibodies specific toward recombinant urease, which were able to dose-dependently inhibit urease adhesion to porcine gastric mucin. *In vivo* experiment on mice infected with *H. pylori* analyzed the effectiveness of treatment with the IgY antibody in the diet as a monotherapy, together with H_2_-receptor blocker (famotidine) or inhibitor of a proton pump (omeprazole), received during 4 weeks starting 1 week after the inoculation. The highest dose of IgY (0.25% in diet) was able to eradicate *H. pylori* infection completely even with IgY used as a monotherapy. Lower doses eliminated the pathogen completely only in a combination therapy (148). Interestingly, the recombinant protein was used as an immunogen in the form of inclusion bodies directly isolated from bacterial cells. Such frequently obtained during the recombinant protein production structures are not considered an optimal material for inducing the antibody production; still, they can trigger T cell response toward the linear epitopes (149). There are data showing that inclusion bodies vaccination *via* oral, intranasal, and subcutaneous routes enables the specific antibody generation (150–152).

### Other H. pylori immunogens

Urease is, by far, the most studied virulence factor of *H. pylori* also in the context of specific IgY production. Several studies also analyzed the therapeutic potential of immunoglobulins Y specific toward other important *H. pylori* proteins. Outer inflammatory protein modulates inflammatory processes of the host cells but is also involved in *H. pylori* colonization of mucosa tissue and, therefore, in the development of more severe gastrointestinal conditions such as duodenal ulcer, gastritis, and gastric cancer. Therefore, infection with OipA-positive *H. pylori* is generally associated with worse clinical outcomes for the patients (135, 153). IgY antibodies developed with the recombinant OipA protein used as an immunogen inhibit the *H. pylori* attachment to the AGS cells *in vitro* (154). Another important factor in *H. pylori* pathogenesis is vacuolating cytotoxin A. VacA cytotoxin is involved in pores formation, which triggers the processes of programmed cell death and modulates the immune response of the host (135). VacA enables the accumulation of CagA—another *H. pylori* oncoprotein—in gastric epithelial cells (155). Anti-VacA IgY antibodies generated by Hong and co-workers were tested on mice as a preventive treatment being added to animals drinking water for 2 weeks prior to inoculation with *H. pylori*. The treatment resulted in a significant reduction in mouse sera levels of anti–*H. pylori* IgG and anti-VacA IgG, indicating that the specific IgY antibody has a protective effect against *H. pylori* colonization. The histological examination of gastric mucosa showed no significant morphological changes when compared to the uninoculated group. The number of eosinophils infiltrations was reduced to 40% of the level of the *H. pylori* infected group, and the immunohistochemical analysis revealed weak or no signal from the reaction with *H. pylori* antigens in the tissue of anti-VacA IgY-treated animals (156, 157). Neutrophil-activating protein (Nap) exhibits strong pro-inflammatory properties as it is capable to induce neutrophils adhesion and activation (135). Hen yolk antibodies specific to Nap protein were able to diminish the capacity of *H. pylori* binding to the AGS cells (158). In the work presented by Attallah et al., avian antibodies generated with 58-kDa antigen (HP58) along with whole-cell lysate were used as a post-inoculation treatment in mice. Passive immunization was carried out as one dose, 1 day after inoculation (both preparations of IgYs) or 1, 4, and 12 weeks later (only anti-HP58 IgY). In all cases, the therapy reduced the percentage of *H. pylori*, signs of inflammation and the degree of gastritis. The most profound reduction in infection was observed in the group receiving specific antibody 1 week after inoculation (159). Another virulence factor crucial for bacteria movement is flagella (135). Anti-flaA immunoglobulin Y production with the use of recombinant immunogen was described in the invention by Peng and co-workers (160). The specific IgG sera level, after bacteria inoculation of mice, was comparable in IgY and clarithromycin treated groups, confirming avian antibody activity.

Because of the limited possibilities of patenting of single antigens and, more importantly, the desire to increase the potency and the functional range of action of the obtained preparations (e.g., hyperimmune IgY), many alternative approaches to the antigen design and preparation can be found in patents description (see section IgY compositions and applications).

One of the patented inventions describes the manufacturing of IgY antibodies and the use thereof as a pharmaceutical product for the treatment of gastritis or gastric ulcer *via* inhibition of *H. pylori* growth and colonization potency. The prepared antibodies were obtained using as a vaccine the mixture of formaldehyde-deactivated whole bacteria cells mixed with recombinant urease alpha and beta subunits. The description not only includes the optimization of the vaccination doses but also verifies the immune response of hens in situations where vaccines were composed as *H. pylori* alone or a mixture of *H. pylori* + *E. coli* or *H. pylori* + *S. cholerasuis*, with much worse anti–*H. pylori* response. The activity toward *H. pylori* of obtained IgYs was confirmed in the agglutination test. Moreover, the IgY antibodies show inhibitory potency in *H. pylori* binding to the AGS cells and in urease activity (161).

Another combination therapy approach included avian antibodies specific toward urease and flagella. In this scenario, the immunogens were isolated and used for vaccination separately. The specific IgYs reduced the number of *H. pylori* present in the stomach and partially suppressed gastritis in infected mice when used alone and completely eradicated *H. pylori* from the stomach and, to some extent, reduced gastritis when used in combination. A strong synergistic effect was observed even in the minimal tested dose at which anti-flagella IgY alone showed almost no activity. A similar synergistic effect occurred in infected mice treated with the combination of anti-urease IgY and *L. acidophilus* although no *H. pylori* inhibition took place in the group receiving only lactic acid bacteria (162). *Lactobacillus acidophilus* is well known as one of the human probiotic strains capable of inhibiting *H. pylori* growth (163); therefore, tested on mice with normal flora *L. acidophilus* anti–*H. pylori* activity is not observed making the synergistic effect even more profound. In the next experiment, the *H. pylori* infection in mice was treated with an anti-urease antibody isolated from egg yolk and compared with rabbit serum IgG with the same specificity. The performance of mammalian IgG in the elimination of *H. pylori* from the mouse stomach was visible but not as effective as avian antibodies (162).

A combination of lactic acid bacteria and IgY antibody is also the subject of invention describing functional food compositions including yogurt, milk drink, and food supplement intended for inhibition of *H. pylori* infections and treatment of gastritis, gastric, and duodenal ulcers. The patented procedures describe the preparation of IgY specific toward flagella and outer membrane fraction, and the antibody was tested in combination with two *Lactobacillus* strains: *L. acidophilus* and *L. casei*. The active ingredients’ potency was tested in a mouse model and in human trials. Volunteers received treatment four times a day for four weeks in a form of fermented milk with active strains and yolk antibodies. The follow-up diagnostic tests revealed a reduction in urease activity [UBT and Campylobacter-like organism (CLO) tests] and lower density of *H. pylori* in the antrum (eight of the 21 treated subjects). The animal *in vivo* studies was designed to verify the prophylactic and the therapeutic potential of the active ingredients fed to the animals in combination or as monotherapy in yogurt. The prophylactic effect was analyzed after 1-week pre-treatment, followed by *H. pylori* inoculation with the 6-week continuation of treatment. In the combination therapy group, 80% of the animals were infection-free (based on urease activity), compared with 50% in the IgY diet group and 60% in the active strains group. In the subsequent experiment, the therapy was introduced after inoculation resulting in 60% infection-free animals in the combination therapy group, 40% and 20% in the groups fed with active strains and IgY, respectively (164).

An interesting therapeutic approach for the use of antigen-specific IgY antibodies is the chemical modification resulting in bifunctional conjugates. *H. pylori*–specific hen antibodies were conjugated to antibiotics (or their derivatives) such as metronidazole, clarithromycin, norfloxacin, or amoxicillin (165). In general, antibody-drug conjugates provide a chance for more focused therapy reducing off-target toxicity, side effects, and treatment time and play an increasingly important role in cancer therapy (166). The synthesis was carried out *via* conjugation between functional groups present in antibiotic structure and protein molecules (e.g., carboxy and amino groups) or based on the preceding introduction of specific functionality, e.g., PEG linker. The conjugation reactions were carried out under mild conditions mainly by the active ester method. The preliminary *in vivo* test on mice suggests the therapeutic efficacy of bifunctional anti–*H. pylori* antibody can exceed 90% (165).

Considering the IgY food products consumed by humans, the items dedicated to the treatment or prevention of *H. pylori* infections seem to be well established on the market with the IgYGate® GastimunHP or Ig-Guard Helico^®^ as an examples (see section IgY market). The scientific literature and patents are focused on *H. pylori* as a human pathogen and therefore the specific antibodies are considered for human use; however, the *Helicobacter* species (referred as non-*H. pylori*) causing gastrointestinal tract infections affects not only humans but also animal species, including pets and farm animals (167).

In the case of *H. pylori*, the literature and patent sources show a high number of possible immunogens that can be used for specific IgY preparation. For most of the cited antibodies, the preventive or therapeutic effect was observed *in vivo*. Vaccines that use whole cells, which are in general easy to prepare and cost-effective, can be designed as a cell lysate or inactivated whole cells. The whole-cell vaccine can induce production of antibodies specific for conformational epitopes, but cell preparation can lead to the loss of some of the extracellular antigens. On the other hand, cell lysate will include intracellular antigens that will not be targeted by the IgY antibodies, but it can also include the synthesized virulence factors before they were secreted from the cells. There were no clear indications which approach resulted in more active preparations due to different study designs and tested subjects. The possible disadvantage of the latter approach is the strong intracellular immunogens that can reduce the immune response toward surface antigens, especially considering the fact that standard culture conditions not necessary induce expression of virulence factors (132). The selection of a single immunogen (or a limited number of pure immunogens) reduces the antibacterial activity to the inhibition of particular antigen function. In the case of *H. pylori*, the most abundant bacterial protein—urease—can be found both inside a cell and on the cell surface, making it the most obvious choice for antigen selection. In fact, the antibodies specific toward urease show not only enzyme activity inhibition but also a reduction in *H. pylori* infection or inflammation (**Table 5**). In addition, antibodies targeting other extracellular virulence factors of *H. pylori* such as flagella or vacuolating cytotoxin A showed promising *in vitro* and *in vivo* activity although the antibacterial activity of IgY specific toward different antigens cannot be easily compared. For a summary of IgY studies, see the **Table 5**.

**Table 5:** Studies concerning the production and application of IgY antibodies specific to *H. pylori*.

Whole-Cell Immunogen	Activity/Properties	Reference
Cell lysate	Mongolian gerbils model; 10 and 1 mg daily for 4 weeks, significant improvement of infiltration of lymphocyte and neutrophil for the 10 mg/day group	Shin et al. (121)
Inactivated whole cells (formaldehyde)	Murine model; 50, 100, 200, and 500 mg/kg IgY twice a day for 18 days; dose-dependent *H. pylori* elimination, improved gastritis and villi injuries; ISA 70 adjuvant	Yang et al. (123)
Whole bacteria	Murine model; 6 mg of IgY every 2 days, four doses, also with sucralfate as an additive; improved *H. pylori* clearance and pathological injury degree; recombinant cholera toxin subunit B (rCTB) as adjuvant	Wang et al. (124)
Inactivated whole cells (formaldehyde), 4-strains	*In vitro*; 10 mg/ml; inhibition of bacteria growth and urease activity (also cross-strain)	Solhi et al. (125)
Cell lysate	Murine model; 2 weeks (dose mouse weight dependent); reduction of number of infected animals based on RUT test	Li et al. (127)

Urease Immunogen	Activity/Properties	Reference
BSA-conjugated with urease peptides	*In vitro*; 1 and 10 mg/ml; inhibition of urease activity	Shin et al. (140)
Urease	Human; 1.5 g IgY three times a day, 4 weeks; reduction of ^13^C-urea breath test values; no specified data concerning adjuvant	Horie et al. (122)
Urease (both subunits)	Human; 900 mg, three times a day, 4 weeks; reduction of ^13^C-urea breath test values	Suzuki et al. (138)
Urease (both subunits)	Mongolian gerbils model; IgY of 25 mg/g and famotidine of 0.16 mg/g, 10 weeks; reduction of MPO and protection against colonization	Namura et al. (137)
Recombinant UreC	Murine model; 60 mg, 28 days in diet or in solution (PBS); stomach tissue inflammation reduction	Malekshahi et al. (139)
Urease (both subunits)	Murine model; 0.5 mg, 28 days with or without LJ88 strain; reduced number of *H. pylori* in stomach	Aiba et al. (143)
Urease (both subunits)	Murine model; 50, 100, 200, or 500 mg/kg of IgY twice a day; 18 days, dose-dependent decrease of *H. pylori* infection	Mony et al. (146)
Recombinant urease	Murine model; 0.25%–0.00025% in diet alone or in combination with famotidine or omeprazole; elimination of *H. pylori* infection; no specified data concerning adjuvant	Yoshikatsu et al. (148)
Urease	Human; standard medical treatment in combination with GastimunHP (15 days) *vs*. standard treatment; reduction of ^13^C-urea breath test values; no specified data concerning adjuvant	*NCT number:* NCT02721355

Other Immunogens	Activity/Properties	Reference
m1 and m2 VacA	murine model; 290 ml/L—access to 100 ml/daily, 2 weeks before inoculation; reduced anti–*H. pylori* and anti-VacA IgG level in sera, reduced signs of inflammation in gastric mucosa	Sook et al. (156)
Recombinant NAP	*In vitro*; IgY of 0.5 and 1 mg/ml; inhibition of *H. pylori* binding to the AGS cells	Borhani et al. (158)
Whole-cell lysate/ 58 kDa antigen (HP58)	Murine model; 0.7 g per animal, one dose 1 day (both IgYs), 1, 4, or 12 weeks after inoculation (only anti-HP58); reduction of number of H. pylori and inflammation in gastric mucosa	Attallah et al. (159)
1. Urease (isolated) 2. Flagella (isolated)	Murine model; IgY anti-urease or anti-flagella used as monotherapy or in combination, or combination therapy anti-urease IgY + *L. acidophilus*; reduction or elimination (combination therapy) of *H. pylori* infection; no specified data concerning adjuvant	Yoshikatsu et al. (162)
Flagella and outer membrane fraction	Human; 4 × 100 ml per day (4 weeks) of fermented milk (active strains and 0.1%–10% IgY); reduction of ^13^C-urea breath and urease CLO test values, reduction of *H. pylori* number in antrum; Drakeol^®^ adjuvant	Seong et al. (164)
Flagella and outer membrane fraction	Murine model; 3 × 1 ml per day (4 weeks) yogurt with active strains in combination with IgY or both active ingredients as monotherapy; reduction of urease activity – infection clearance; Drakeol^®^ adjuvant	Seong et al. (164)
Recombinant flagella	Murine model; 60 mg in 1 ml per day, 4 weeks; reduction of IgG serum level	Peng et al. (160)

If not specified in the Activity/Properties column, then FCA/FIA was used as an adjuvant.

## Escherichia coli

Human and veterinary medicine is alarmed at the antibiotic/multidrug resistance developed by *Escherichia coli*. Several strains of this gram-negative bacteria can induce lethal bloodstream infections (of community or hospital origin) (236). *E. coli* has an immense ability to accumulate genes—probably from other bacteria—responsible for AMR, and it can act as a donor of resistance genes (237). Infections caused by pathogenic strains of *E. coli* are classified into: enterohemorrhagic (EHEC and STEC), enterotoxigenic (ETEC), enteroagreggative (EAEC), enteroinvasive (EIEC), enteropathogenic (EPEC), and diffusely adherent (DAEC).

Enterohemorrhagic *E. coli* (EHEC) is a common foodborne pathogen that leads to gastrointestinal infections, hemolytic uremic syndrome, and renal failure (238). The colonization of host tissues by *E. coli* can be inhibited with IgY specific to virulence factors such as intimin and proteins that facilitate contact between host intestinal cells and pathogen: translocated intimin receptor (Tir), as well as EPEC secreted proteins (Esp) EspA, EspB, EspD, and EspF (173). The genes coding intimin are present mainly not only in EPEC but also in some EHEC *E. coli* strains (239). Girard et al. described the potential of anti-intimin and anti-Tir IgYs (5 mg/ml) to reduce *E. coli* adherence tested in porcine ileal in *in vitro* organ culture model, whereas antibodies specific to EspA, EspB, and EspD did not reduce the adherence of EHEC (O157:H7) (173). In a similar work, Cook et al. demonstrated that anti-EspA, anti-intimin, and anti-EscF IgY and IgG antibodies are not only able to bind recombinant and native antigens of EHEC but also prevent the adhesion of EHEC to HeLa cells. The observed differences in reduction of bacteria adhesion between experiments where anti-EspA IgY was tested may result from different protocols of adherence assays used by the authors (240).

EHEC strains are known to produce Shiga toxins (Stx) following their passage through the acidic barrier of the stomach and subsequent adherence to the large intestine. Subsequently, Stx enters the blood circulation and reaches the kidneys as the major target organs (241). Two types of Stxs have been identified: Stx1 and Stx2 (sharing 56% of the overall amino acid identity) (242). Different subtypes of Stxs are produced by EHEC strains. Several research studies have been focused on the development of Stxs neutralizing agents including specific IgYs. Wang et al. reported that IgYs obtained after immunization of hens with Stx1 disrupted binding of Stx1 to the HeLa cells in a dose-dependent manner. Moreover, anti-Stx1 IgYs had a protective effect on BALB/c mice when challenged with Stx (5 LD_50_ dose, calculated by the method of Reed and Muench). The protective effect was observed for all animals when the highest tested dose of IgYs (3.6 mg) was applied (174). Another report concerning the preventive effect of IgYs against Stxs suggests that IgYs administrated intraperitoneally/intravenously (up to 100 mg/kg) specific to Stx1 or Stx2 are able to reduce the mortality of mice infected with a lethal dose of corresponding Stx [625 μg/kg partially purified Stx1 (2.5 LD_50_) and 17.4 μg/kg partially purified Stx2 (2 LD_50_)]. The results concerning a time dependency of the treatment with specific IgYs suggest that, to neutralize Stxs, they need to be given before Stxs bind to target organs. In addition, anti-Stx2 IgY antibodies administered orally to mice prevented death caused by streptomycin resistant *E. coli* strain producing Stx2, GPU993-S. This proves that IgY-mediated Stx2 inactivation in the intestine can be a powerful therapeutic tool to fight the infection when co-administrated with antibiotics (175). IgYs specific to Stx2e, a toxic factor responsible for diarrhea and edema in swine, have shown a therapeutic potential as evaluated with the Kunming mice challenged with the Stx2e. Animals were injected intraperitoneally with specific IgYs (at different dilutions: stock solution, 1:2, and 1:4) followed by injection of Stx2e after 6 h (839 μg/ml, 0.3 ml; LD_50_ is not specified). Control groups received saline, IgYs alone, or Stx2e alone. The results showed the IgY-dependent reduction in Stx2e toxicity (176). Ma et al. described the application of serotype-specific IgYs and chitosan nanoparticle (CN) conjugates that were able to selectively target STEC in the gastrointestinal tract. Purified lipopolysaccharides (LPSs) extracted from *E. coli* membrane (purified O-antigen repeating units) were used as immunogens to develop specific IgYs that were further conjugated with CN at different CN : IgY ratio (10:1, 10:2, and 10:4). Conjugates (10:2) revealed the greatest antimicrobial potency against the *E. coli* O157:H7 strain. The *in vivo* evaluation of the CN-IgY conjugates was performed on the *Caenorhabditis elegans* animal model and demonstrated a strong and specific activity against *E. coli* strains, which suggested that it might be applied to inhibit the spread of pathogens without suppressing the beneficial bacteria (177). The activity of IgY antibodies specific toward Shiga toxin type 2 recombinant subunit (Stx2B) was analyzed in both *in vitro* (Vero cells) and *in vivo* (mice) models in comparison with rabbit sera IgG antibody activity. The *in vitro* study showed that both IgG- and IgY-specific antibodies (affinity purified) were able to neutralize the cytotoxic effect of Shiga toxin; however, the IgG antibody was four times more active than the chicken immunoglobulins, providing 100% protection at a concentration of 1.09 µg/ml (IgG) in comparison to 4.38 µg/ml (IgY). As for the *in vivo* experiment, the mice were injected intravenously with the Shiga toxin type 2 holotoxin (5 LD_50_ dose, calculated by the method of Reed and Muench) or with preincubated toxin-antibody (IgG or IgY) mixture. All mice injected with the toxin alone died by the third day of the experiment, whereas all mice receiving IgY at a concentration of 8.75 µg/ml alongside the toxin survived until the end of the experiment (4 days). The IgG antibody was tested at a maximal concentration of 0.55 µg/ml, resulting in a 75% survival rate (189). In a similar experiment performed by Fathi et al., the inactivated toxin isolated from the *E. coli* O157:H7 (stx1+/stx2+) strain was used for the production of the IgY antibody that was able to provide 100% toxin neutralization at a concentration of 2 mg per mouse when tested *in vivo* in the mice model (Stx LD_50_ was calculated as 0.140 μg/kg, 5 LD_50_ dose applied for mice challenge) (190).

The patented immunoglobulin Y generated with recombinant type I Shiga toxin subunit B possesses the inhibitory potency toward the cytotoxicity of Shiga toxin as analyzed on Hela cells *in vitro*. The experiment showed a dose-dependent protective effect on cells viability with the IC_50_ equal to 0.428 μM and complete inhibition of cytotoxic effect at an IgY concentration of 17.5 μM. The *in vivo* protective effect of specific IgY antibodies was verified on mice with a 100% survival rate in the group receiving an intraperitoneal injection of toxin (5 LD_50_, 65 μl) preincubated with 3.6 mg of IgY. In the group where the concentration of 1.2 mg of IgY was used, 41.67% (five of the 12) animals were alive after 7 days, and 8.3% (one of the 12) in the group in which the toxin was mixed with 0.4 mg of IgY. No animals in the control group survived (191).

IgY antibodies specific to LPS described by Zhen et al. showed antibacterial activity in mice with induced endotoxemia (intraperitoneal LPS injection at 20 mg/kg). The 70% of mice treated with specific IgYs (200 mg/kg) survived more than 7 days after the infection, whereas none from the saline-injected control group did. In addition, an increased level of anti-inflammatory cytokine IL-10 was observed resulting in the downregulation of the TNF-α production in the serum of endotoxemia mice (178).

Enterotoxigenic *E. coli* strains are mainly dangerous for young animals, primarily piglets and calves, and for humans. ETEC infection is a major cause of diarrhea among travelers and children in the emergent countries. The manifestation of infection includes serious diarrhea, which leads to an increased mortality (243). Although maternal antibodies protect neonates during first days of life, after a relatively short period a risk of ETEC infection rises. The development of vaccines and/or supplements that can confer passive protection against ETEC is of high need. The main virulence factors of ETEC are adhesins and enterotoxins. The diarrhea arises as a result of binding heat-labile enterotoxin (LT) and heat-stabile enterotoxin (ST). Two ST toxins, STa and STb, are mainly associated with piglets, calves, and humans, and porcine ETEC strains, respectively (244). The IgYs specific to fusion enterotoxin protein LTB-STa-STb (Bab) obtained by You et al. were able to neutralize the toxic effect of ST. In a suckling mouse model, anti-Bab IgYs were able to neutralize STa (2× dilution of IgYs) and STb (32× dilution of IgYs) (179). Another protein important for ETEC invasion is K99 fimbrial protein that enables colonization of the host epithelium. The main component of K99 is FanC subunit (245) used for the development of anti-FanC IgY antibodies that were able to specifically bind FanC protein of ETEC in ELISA assays (168). Anti-FanC IgY has the potential to be developed for both diagnosis and treatment purposes in relation to ETEC infection.

Because ETEC-specific IgYs are considered a potential alternative to antibiotics, an approach to enrich animal food with IgYs of immunized hens has been undertaken. Han et al. used egg yolk powder from immunized hens (HEYP) as a food additive. Three formaldehyde-inactivated *E. coli* strains (ETEC K88, ETEC K99, and ETEC 987P) and corresponding single strains separately served as immunogens. The weaned pigs were treated with HEYP (3 g/kg), which was followed by an analysis of their immune response, diarrhea incidence, and intestinal permeability. The results indicated that an addition of egg yolk powder to the basal diet attenuated diarrhea improved intestinal health and serum immunity. Authors did not observe significant differences in the dosage effects of HRYP. Consequently, anti-HEYP IgY formulations used in the weaned pigs diet could be an effective antidiarrheal additive (180). The potency of IgYs specific to ETEC K88 as a bacteriostatic factor was further evaluated by Han et al. *in vitro* and on the mice intestinal infection model. The results from *in vivo* experiments suggest that the bacteriostatic mechanisms include agglutination of bacteria, reduction of adherence, and destruction of the integrity of bacterial cell wall. Mice were treated (oral administration) with specific IgY solutions (32, 16, and 8 mg/ml; 250 μl) followed by ETEC K88 inoculation. The results indicated the protective potency of medium (16 mg/ml) and high (32 mg/ml) doses of anti-ETEC IgYs to reduce intestinal inflammation and prevent enteric pathogens infection (181). The hypothesis that dietary supplementation with IgY antibodies specific to *E. coli* may improve the intestinal function of early weaned piglets and suppress the *E. coli* infection was examined by Li et al. The piglets were treated with commercial *E. coli*–specific IgY (500 mg/kg), and the effect was compared to colistin sulphate and entramycin. The results demonstrated that antibiotics decreased not only the growth of *E. coli* but also the growth of beneficial *Lactobacillus* sp. in the intestines, which was not the case when specific IgYs were supplemented as those reduced the *E. coli* proliferation and did not affect the *Lactobacillus* sp. population (182).

In addition, the patent descriptions present the IgY antibodies specific toward pathogenic *E. coli* strains: K88, K99, or 987P as a treatment of diarrhea in piglets. The IgY antibodies generated by the inactivated bacteria of these strains were served as an active ingredient of milk powder for piglets (246). In another invention, the mixture of pilin protein isolates from three *E. coli* strains (987P, K88, and K99) was applied for the generation of IgY antibody (247) designed as an active ingredient of milk powder for piglet ETEC diarrhea prevention (248).

Infectious agents, including ETEC, are main causes of severe diarrhea. The intake of colostrum by newborns and passive immunization strategies can prevent harsh symptoms of infection. Vega et al. proposed the immunization strategy called IgY DNT focused on the prevention of the neonatal calf diarrhea based on application of IgYs specific to various pathogens including group A rotavirus, coronavirus, ETEC, and *Salmonella* sp. Hens were first immunized with commercially available vaccines, including *E coli* J5. Newborn calves received fresh milk or fresh milk supplemented with powdered IgY DNT (10 mg/ml), which resulted in a significant reduction in diarrhea severity in IgY DNT-treated animals (183). The effect of dietary probiotics in combination with anti-K99 IgYs was evaluated by Karamzadeh-Dehaghani et al., who used whole formalin inactivated *E. coli* K99 cells (O101:K99^+^) as the immunogen. Egg yolk powder (1 g/day) was applied as a single food additive or together with a commercial probiotic mixture (3 g/day, Hypro-calves) that contains dextrose and seven bacteria species (*Enterococcus faecium*, *Pediococcus acidilactici*, *Streptococcus thermophilus*, *Lactobacillus bulgaricus*, *Lactobacillus acidophilus*, *Lactobacillus rhamnosus*, and *Bifidobacterium bifidum*). This combination ensured a lowered incidence of diarrhea in calves (184).

EPEC is one of the most common etiological factors of diarrhea in the emergent countries. First IgYs specific to EPEC antigenic fractions were described by Amaral et al., who used whole EPEC cells as an immunogen (169). An intriguing feature of enteropathogenic *E. coli* (EPEC) is the bundle-forming pilus (BFP), a critical adhesion factor that significantly boosts the bacteria’s virulence. This pilus is composed of the BfpA protein, which plays a key role in its formation. Pre-incubation of EPEC with IgYs specific to BfpA inhibits the adherence of EPEC to HeLa cells. In addition, pretreated anti-BfpA IgY (400–800 μg) inhibits the growth of EAF(+) but not the EAF(−) strain of EPEC (170). Melo et al. indicated that anti-BfpA IgYs that blocked BfpA on the surface of EPEC-EAF(+) inhibits its ability to induce apoptosis of HeLa cells which is important since the induction of apoptosis of epithelial cells is the factor that supports the growth of EPEC (171).

As a potential tool for the treatment of meningitis triggered by *E. coli* infection Mwale et al. developed scFv antibodies specific to OmpA protein responsible for *E. coli* adhesion to the endothelial cells. The hens were immunized with different OmpA variants (full length and truncated). For the construction of monoclonal IgYs libraries, a phage display technology followed by panning was applied. The reactivity of the obtained IgYs against *E. coli* was confirmed by immunofluorescence (172).

One of an important poultry disease is an infection with avian pathogenic *E. coli* (APEC, a subgroup of extraintestinal pathogenic *E. coli*, ExPEC). Kariyawasam et al. utilized *E. coli*–specific IgYs in their research to provide active and passive protection for chickens. The antigens used for immunization were live *E. coli*, inactivated *E. coli*, and individual *E. coli* antigens: LPS, type 1 pilus adhesin (FimH), P pilus adhesin (PapG), and aerobactin outer membrane receptor (IutA). Passive antibodies (100 mg, 1 ml) administered among hens provided protection against the homologous serotypes challenge (except anti-FimH IgY). Anti-PapG and anti-IutA IgY were effective also when heterologous infection was induced (185). For the induction of an active protection, they used nonvirulent mutants of APEC (ΔgalE, ΔpurA, and ΔaroA) as well as a wild type (which induced a stronger response) showing that vaccination provided protection in a serogroup-specific manner (249).

A growing interest with regard to the use of natural additives for livestock farming resulted in a growing amount of research focused on the development of products that can replace or support antibiotic treatment. One of the approaches designed to confer protection against *E. coli* in hens is the application of *E. coli*–specific egg yolk antibodies as a dietary additive (up to 0.4% *w/w* of specific or nonspecific IgYs, seven different dietary treatments). Mahdavi et al. revealed that a 3-week intake of at least 0.2% *w/w* of specific IgYs improves the immune response of hens challenged by *E. coli* strain used for immunization (O78:K80) (186). Karthikeyan et al. performed meta-analysis concerning the use of chicken IgY antibodies as a prophylactic/therapeutic agent to decrease the risk of infectious diarrhea. The studies under analysis included research performed with the participation of piglets, mice, poultry, and calves. The results support the hypothesis that IgY can be used as an alternative therapy to treat *E. coli* infection and its consequences such as diarrhea (250).

The patented solutions for the generation and application of egg yolk antibodies specific toward *E. coli* antigens are intended not only for use by humans but also for veterinary applications as its pathogenic strains are responsible for serious and even life-threatening infections, especially in newborn mammals without fully developed gut microbiome (251, 252). The approach toward vaccine design included the use of whole bacteria antigens, mainly based on selected enterotoxigenic *E. coli* strains (ETEC), a mixture of a few *E. coli* strains, or pure (recombinant) proteins used as an immunogens. There are also many patented examples of utilization of *E.coli* as one of the multiple different bacterial strains used for the production of hyperimmune IgY antibodies in eggs, which will be further discussed in section IgY compositions and applications.

The *E. coli* 0157:H7 was applied as an immunogen for hen immunization as a whole bacteria preparation (formaldehyde-fixed) or as selected fractions of a culture (e.g., pili and adhesins). The specific antibodies were prepared in a form of feed additive based on soybean hulls. Their activity in the prepared composition was confirmed by ELISA. Finally, the feed additive was added to the farm cattle diet. Five of the tested animals were positive for *E. coli* 0157:H7 at the beginning of the experiment; however, after 30 days of treatment, tests were negative, confirming the therapeutic potency of the IgYs (187).

TEM β-lactamases are one of the most important and recognized factors responsible for antibiotic resistance in *E. coli* and other gram-negative bacteria (253) and, therefore, are a good candidate as a target for specific antibodies capable not only of recognizing and binding the protein but also of inhibiting its enzymatic activity. In the invention describing the generation of IgY antibodies directed against *E. coli* TEM-1 β-lactamase, the authors performed the functional analysis of the amino acid sequence of the enzyme and selected two peptidyl fragments building both the active site and binding pocket of the protein. The epitopes were used as immunogens in the form of KLH-conjugates. The invention also presents the generation of immunoglobulins Y specific against whole-enzyme TEM-1 and whole-cell bacteria. The whole-cell immunogen was prepared by thermal or e-beam inactivation of a culture. The generated IgYs were tested as precipitation-purified and affinity-purified preparations. The growth inhibition of TEM-1 producing *E. coli* was observed for precipitation-purified antibodies specific toward whole-cell antigen and for affinity-purified IgYs, specific toward one of the selected epitopes and full-length protein used alone or in combination with ampicillin (254).

The OMP A (OmpA) is another bacterial protein of great interest as an antigen. The patented invention describes the production of recombinant *E. coli* OmpA protein and subsequently immunoglobulin Y specific toward OmpA. The antibody was able to cross-react with OmpA protein expressed by most of the other tested bacteria belonging to the *Enterobacteriaceae*. The anti-OmpA IgY inhibited the *E. coli* E44 invasion while tested on glioma C6 cells (255). The IgY antibody derived from the mixture of the OMP and pilin proteins (*E. coli* O78 strain) used as an immunogen showed protection against pathogenic *E. coli* in chickens (256). Another interesting antigen used for hen immunization is intimin, a membrane protein responsible for the attachment of *E. coli* to the epithelial host cells, produced and frequently used for identification of enteropathogenic *Escherichia coli* (257). The product of eae gene cloned from the genome of the *Escherichia coli* O157: H7 strain was utilized for the production of recombinant intimin protein and further for the generation of avian Y antibodies in hens (258).

Among the patented solutions aiming to overcome the diversity of enterotoxigenic *E. coli* strains, there are several describing fusion proteins used subsequently as antigens. In one invention, the fusion protein is based on epitopes selected from adhesins of nine different strains of ETEC. The utilization of fusion protein allowed the generation of specific IgY antibodies capable of recognizing all adhesins selected for fusion protein design. The potency to inhibit the adhesion of ETEC to mammalian cells was confirmed not only for strains chosen for antigen design but also for other isolated *E. coli* strains responsible for clinical outbreaks (259). Another invention describes the fusion protein (188) that includes functionally connected adhesion proteins: flagellin FliC and EtpA (260). The analysis of hen yolk antibodies included not only IgY activity and stability but also tests in mice, which showed a significant inhibition of cell adhesion in two of the three strains tested, as analyzed on the basis of PCR detection of ETEC-specific enterotoxin primers (188). The fusion plasmids that included three copies of pig-derived enterotoxigenic *E. coli* (K88ac) flagellar gene (fliC) or two copies of human enterotoxigenic *E. coli* fliC gene were also used to produce the recombinant proteins and further applied for hen immunization, resulting in specific antibodies production presenting the ability to inhibit the bacteria adhesion to the intestinal porcine enterocytes (IPEC-J2) (261) or showing *in vivo* anti-adherent activity in mice (262). In another example, the K88ac strain was a source of adhesin gene faeG and, together with adhesin gene fedF of strain F18ac, was used for recombinant fusion protein production. The IgY antibody produced by immunization with FaeG-FedF fusion protein allowed for reduction in bacteria adhesion to epithelial cells *in vitro*, and the diarrhea incidents in piglets *in vivo* (263). The trivalent fusion protein, described in another invention, was designed on the basis of the sequences of enterotoxin genes eltB, STI, and STII of infecting pigs strains of *E. coli*, with the region encoding flexible amino acid linker connecting genes. The recombinant fusion protein was utilized for the generation of IgY antibodies together with the K88 strain of *E. coli* natural pili and/or inactivated whole bacteria, and the *in vitro* effect of obtained antibodies on the *E. coli* K88 growth was confirmed (264).

The interesting antimicrobial application of anti–*E. coli* IgY antibodies (generated by inactivated bacteria) is the modification of polymer—polybutylene adipate terephthalate—surface with a layer of IgY-functionalized chitosan hydrogel that provides this compostable packing material with antimicrobial characteristic and represents a non-toxic, antibiotic-free and organic modification. The functionalized material provided a significant reduction in *E. coli* growth, which was verified *in vitro* (265). For a summary of IgY studies, see the **Table 6**.

**Table 6:** Studies concerning the production and application of IgY antibodies specific to *E. coli*.

Immunogen	Activity/Properties	Reference
Recombinant FanC protein	*In vitro*, immunoassays; anti-FanC IgY specifically recognize FanC protein	Nasiri et al. (168)
Formaldehyde-fixed EPEC	*In vitro*, immunoassays; obtained IgYs recognize virulence factors of *E. coli*	Amaral et al. (169)
Recombinant BfpA protein	*In vitro*, *in cellulo*; inhibition of *E. coli* expressing BfpA protein growth *in vitro*	de Almeida et al. (170)
Recombinant BfpA protein	*In cellulo*; IgY as a tool for describing induction of apoptosis and caspase-3 activation in host cell	Melo et al. (171)
OmpA, full recombinant protein and peptide	*In vitro*, phage display technology, evaluation of the diagnostic potency OmpA scFv antibodies	Mwale et al. (172)
Recombinant Tir, EspA, EspB, EspD, and intimin proteins	Porcine ileal *in vitro* organ culture model; inhibition studies weaned pigs; oral administration, anti-intimin and anti-Tir IgY reduce adherence of *E. coli* in porcine ileal IVOC, anti-intimin IgY reduce severity of attaching and effacing lesions in the small intestine of pigs	Girard et al. (173)
Recombinant Stx1B protein	Murine model; intraperitoneal injection ensure protective activity against binding Stx1	Wang et al. (174)
Formaldehyde-fixed partially purified recombinant Stx1 and Stx2	Murine model; intraperitoneal or intravenous injection ensure protective effect of specific IgY, reduced mortality of infected mice	Neri et al. (175)
Formaldehyde-fixed Stx2e and recombinant Stx2e B proteins	Murine model; anti-Stx2e IgY and anti-Stx2e B IgY could protect mice from Stx2e challenge; oil adjuvant	Feng et al. (176)
O-antigens isolated from LPS od 7 different *E. coli* strains (O26, O45, O103, O111, O121, O145, and O157)	Synthetic gastric (SGF) and intestinal (SIF) fluids, *Caenorhabditis elegans* model; chitosan nanoparticles-IgY conjugates act as a specific antimicrobial agent; commercial GeneTel Laboratories adjuvant	Ma et al. (177)
Formaldehyde-fixed *E. coli* O111	Murine model of endotoxemia; intraperitoneal injection ensure protective effect of specific IgY, reduced mortality of infected mice	Zhen et al. (178)
Formaldehyde-fixed ETEC F4 cells, recombinant Bab protein	Murine model; injection; anti-Bab IgY have the capacity to neutralize the toxicity of Sta and STb	You et al. (179)
Formaldehyde-fixed K88, K99 and 987P *E. coli* strains	Weaned pigs; oral administration; hyperimmunized egg yolk powder reduces the *E.coli* invasiveness; without adjuvant	Han et al. (180)
Formaldehyde-fixed K88 (BNCC 125988)	Murine model; oral gavage; prophylactic, ameliorating activity of anti-ETEC IgY, without adjuvant	Han et al. (181)
*E. coli*	Weaned pigs; oral administration; suppression enteric *E. coli* growth, commercial Zyme Fast (Changsha) Biotechnology adjuvant	Tan et al. (182)
*E. coli* J5	Newborn calves; oral administration (milk supplementation), after administration of IgY anti-DNT reduction of severity and duration of the diarrhea was observed	Vega et al. (183)
Formaldehyde-fixed *E. coli* K99	Calves; oral administration (milk supplementation); specific IgY administered with probiotics reduces diarrhea prevalence	Karamzadeh-Dehaghani et al. (184)
Killed *E.coli*, FimH, PapG, IutA, LPS	Broiler chickens; intramuscular injection; antibodies administered among hens provided protection against homologous challenge; Quil A adjuvant	Kariyanwasam et al. (185)
Killed *E. coli* O78:K80	Broiler chickens; dietary supplementation; specific IgY improve intestinal condition through immune system	Mahdavi et al. (186)
Inactivated whole cells (formaldehyde) and cells fractions	Cattle; average 10 lbs of feed additive per day; infection clearance; no specified data concerning adjuvant	Nash et al. (187)
Recombinant FliC and EtpA fusion protein	Murine model; single dose of 50 µl IgY solution 1 h after inoculation; in two of the three groups cells adhesion was inhibited; white oil, aluminum stearate, Span-80 adjuvant	Peng et al. (188)
Shiga toxin type 2 recombinant subunit	Murine model; preincubated mixture of toxin and antibody of 0.55 and 8.75 µg/ml; toxin neutralization at higher dose	Parma et al. (189)
Inactivated Shiga toxin	Murine model; preincubated mixture of toxin and antibody of 0.5, 1, and 2 mg per mice; dose-dependent toxin neutralization	Fathi et al. (190)
Shiga toxin type I recombinant subunit B	Murine model; preincubated mixture of toxin and antibody of 0.4, 1.2, and 3.6 mg; dose-dependent toxin neutralization	Wang et al. (191)

If not specified in the Activity/Properties column, then FCA/FIA was used as an adjuvant.

## Salmonella typhimurium

Control of *Salmonella* infection in poultry, cattle, and pigs is an important public health issue because is often transferred to humans through the consumption of infected meat, milk, and eggs. Infections caused by non-typhoidal *Salmonella* are estimated at around 93.8 million of cases and cause 155,000 of deaths worldwide (266). The majority of human infections are caused by *S. enteritidis* and *S. typhimurium*, serotypes of NTS. The possibility of contaminated food products finding its way to the market (such as multicountry outbreak of salmonellosis caused by Belgium chocolate products in 2022) makes it important to monitor all *Salmonella* cases (267). The life-threating symptoms of salmonellosis concern dehydration and other complications that it causes in children and elderly patients. Antimicrobial therapy combined with a supply of electrolytes is the common treatment strategy against gastroenteritis caused by *S. typhimurium*. However, increasing antibiotic resistance (including MDR) is becoming a worldwide therapeutic problem (268).

It is possible to obtain IgY antibodies from chicken deliberately immunized with *S. typhimurium*. Chalghoumi et al. pioneered the development of IgYs that, as a result of double immunization (OMPs as an antigens), are able to specifically recognize *S. enteritidis* and *typhimurium*. In addition, the authors observed that the titer of IgY obtained was affected by the type of adjuvant utilized. The use of Freund`s adjuvant resulted in higher antibodies titers than the application of the immunostimulating complex matrix; however, the amount of an antigen for immunization with Freund’s adjuvant (100 μg) was also higher than for the immunostimulating complexes matrix (10 μg) (192). The production of IgY antibodies specific to *S. typhimurium* and *S. enteritidis* was also performed in quails by Esmailnejad et al. As an immunogen, heat-inactivated *Salmonella* cells and Freund`s adjuvant were used (193).

In their later work, Chalghoumi et al. demonstrated that specific anti-OMP IgYs are able to block the adhesion (>31.25 μg/ml) and growth (250 μg/ml) of *Salmonella* on a monolayer of Caco-2 cells. Noteworthy, non-specific IgYs also had a protective effect at higher concentrations. The authors hypothesized that this effect could be the result of other yolk components that were not removed from the formulation (194). Chalghoumi et al. also evaluated the potential of feed supplementation with IgY antibodies raised against OMP proteins of *S. enteritidis* and *S. typhimurium*. The results showed that IgYs were not able to protect against the colonization of the chicken cecal because of their denaturation and degradation inside the gastrointestinal tract; however, they revealed that other components of egg yolk powder are beneficial for infected hens (195).

Li et al. demonstrated that oral administration of specific anti–*S. typhimurium* IgY (0.4 ml, 20 mg/ml) confers passive protection against *S. typhimurium* infection deliberately induced in mice. Treatment with specific IgY changes the mucosal immune response of gut-associated lymphoid tissue through the reduction in the lymphocytes populations in certain areas and the weakened increase in the proinflammatory cytokines such as INF-γ and TNF-α (196).

The determination of the IgY antibodies level and their specificity is also used for monitoring serological response of infected chickens (269), differentiation between infected and vaccinated animals (DIVA strategy) (270), and description potential of cross-protective immunity against challenge with *S. typhimurium* and *S. enteritidis* (271). Senevirathne et al. developed attenuated *S. typhimurium* strain (O-antigen–deficient; JOL2377) that induces a beneficial immune response after mucosal and parenteral immunization. IgYs produced during immunization do not recognize LPS (272). For a summary of IgY studies, see the **Table 7**.

**Table 7:** Studies concerning the production and application of IgY antibodies specific to non-typhoidal *Salmonella*.

Immunogen	Activity/Properties	Reference
Outer membrane proteins from *S. enteritidis* and *S. typhimurium*	*In vitro*; ELISA; IgY antibodies specific to *Salmonella* OMPs; FCA or immunostimulating complexes matrix adjuvant	Chalghoumi et al. (192)
Heat-inactivated *S. enteritidis* and *S. typhimurium*	*In vitro*; ELISA, Western blotting IgY antibodies specific to *Salmonella*	Esmailnejad et al. (193)
Outer membrane proteins from *S. enteritidis* and *S. typhimurium*	*In vitro*/*in cellulo*; adhesion and growth inhibition assays blocking the cell attachment and growth of *Salmonella* in the IgY-concentration dependent manner	Chalghoumi et al. (194)
Outer membrane proteins from *S. enteritidis* and *S. typhimurium*	Chickens; oral administration; lack of protective effect after specific IgYs administration	Chalghoumi et al. (195)
Formaldehyde-fixed *S. typhimurium*	Mice model; oral administration; inhibitory activity of specific IgY against inflammatory process	Li et al. (196)

If not specified in the Activity/Properties column, then FCA/FIA was used as an adjuvant.

## Campylobacter jejuni

Another bacterium responsible for gastroenteritis is *Campylobacter*. The infection symptoms are diarrhea, fever, abdominal pain, and headache. The symptoms develop within 2 to 5 days, and only 5% to 10% of infected people require hospitalization (273). Infection is the most dangerous for very young children, elderly, and immunosuppressed people. In more complicated cases, there is a risk of being affected with the Guillain–Barrés syndrome (1 in 1,000 infections) (274). The Foodborne Diseases Active Surveillance Network (FoodNet) reports that 20 cases in 100,000 are diagnosed with *Campylobacter* infection every year. According to the WHO, the sources of transmission are similar to the salmonellosis, food animals (e.g., poultry and cattle, pigs). It was noted that, because of treating food animals with fluoroquinolones, the existing trend of fluoroquinolones resistance in *C. jejuni* has increased (274). Prevention of *Campylobacter* infections consists in testing each chain of food production and all stages of poultry production, disinfection, and good hygienic practices.

Chickens are considered the reservoir host of *C. jejuni*, and their meat is a primary source of infection. On the basis of the previous studies concerning specificity of maternal IgY antibodies from *C. jejuni* infected hens, Al-Adwani et al. produced IgY antibodies specific to colonization-associated proteins (CAPs) of *C. jejuni* (CadF, FlaA, MOMP, FlpA, and CmeC). CAPs are essential for *C. jejuni* adherence to the host cells, motility, and survival. Anti-CAP IgY revealed broad spectrum reactivity against heterologous strains of *C. jejuni*, which suggests high potential of this antibodies as a passive immunotherapeutics. In addition, the said authors revealed a significant reduction in the binding of *C. jejuni* to the chicken hepatocellular carcinoma cells (LMHs) when treated with anti-CadF, MOMP, and CmeC IgY antibodies (1:10, IgY:media) (197). The effectiveness of short term passive immunotherapy was evaluated in another work on anti-CAP IgY performed by Paul et al. For the immunization, there were used previously described CAPs with additional Peb1A and JlpA. The addition of hyperimmunized egg powder (single specificity and a mixture of different anti-CAPs) as an additive to the animal feed [10% (*w*/*w*) egg yolk in feed] did not cause the significant reduction in *C. jejuni* colonization. The authors suggest, similarly to the Chalghoumi et al. (195), that IgYs might degrade or/and denature during the passage through the chicken intestine (198). A different immunization strategy was adopted by Hermans et al. In their research, whole-cell lysate and hydrophobic fraction of the cells serve as immunogens. Obtained IgYs [5% (*w*/*w*) egg yolk in feed] were used as passive therapy 3 days after the inoculation of hens with *C. jejuni*. The administration of IgYs significantly reduced the amount of cecal *C. jejuni*. In addition, the Western blot combined with mass spectrometry analyses made it possible to describe immunodominant antigens of *C. jejuni*. These proteins could be promising vaccines because of their conservativeness (199). Thibodeau et al. decided to reveal whether different immunization strategies affected the activity/performance of obtained antibodies. They used for the immunization whole cells of *C. jejuni* from four different strains and OMPs from different strains. The immunogen was delivered orally in the case of whole *C. jejuni* cells or injected subcutaneously as a formalin-inactivated cells or OMP. Results show that oral administration and injection activate the production of antibodies able to recognize proteins from homologous and heterologous strains of *C. jejuni*. The characterization of the obtained IgYs revealed their similar activity against *C. jejuni* of corresponding strains; however, the agglutination occurred only if homologous strains and IgYs were used, probably because of higher avidity (200). In another research, Garba et al. prepared encapsulated formulation of egg yolk powder containing *C. jejuni*–specific IgY. In the *in vitro* assays (e.g., agglutination and mobility) encapsulated antibodies revealed similar properties but with a lower degree. An animal model showed that antibodies did not significantly prevent cecal colonization (201). These results contradict from achieved by Hermans et al. (199); however, the authors suggest that the optimization of the conditions is necessary.

Vandeputte et al. performed studies on the basis of previous results presented by Hermans et al. (199), which clearly suggest the possibility of conducting passive immunotherapy in chickens by means of IgY antibodies. The authors immunized hens with two types of immunogen: bacterin that was composed of heterogeneous strains of *C. jejuni* and *Campylobacter coli*, and subunit vaccine composed of highly conserved and prevalent proteins selected in agreement with previous studies, responsible, e.g., for chemotaxis or amino acid transport. Both prophylactic and therapeutic efficacy of the IgY were evaluated [5% (*w*/*w*) egg yolk in feed]. A significant reduction in *Campylobacter* titers was observed after therapeutic administration and diminished infection susceptibility in the prophylactic protocol (202).

Treating chickens with IgY antibodies specific to enterobactin, conserved and important siderophore for gram-negative bacteria was a promising idea for controlling *C. jejuni* colonization of the intestine. The immunization of hens with the Ent conjugate elicited a strong response and production of specific IgYs. Nevertheless, the supplementation of feed [2% (*w*/*w*) egg yolk in feed] with egg yolk powder enriched with anti-Ent IgY did not confer protection against *C. jejuni* colonization in the intestine. The said researchers evaluated the specific IgY titers in different compartments, and the results revealed a significant decrease in IgY titer in the gizzard contents, which could be the cause of insufficient protection (203).

When it comes to IgY antibodies and *C. jejuni* infections, a large part of the available literature consists of reports on monitoring the response of the animal’s immune system after vaccination (275–282). Different variants of the vaccines are described, not only *C. jejuni* cells but also, for example, nanoparticle-encapsulated OMPs (283), glycoconjugates (284), or probiotics (285). An interesting aspect is also vaccination with *S. typhimurium* that produce *C. jejuni* CjaA protein, the most frequently tested *Campylobacter* antigen (286). IgY antibodies also served as a immunological tool for describing how B lymphocytes influence the clearance of *C. jejuni* in the chicken intestinal tract (287). For a summary of IgY studies, see the **Table 8**.

**Table 8:** Studies concerning the production and application of IgY antibodies specific to *Campylobacter jejuni*.

Immunogen	Activity/Properties	Reference
CadF, FlaA, MOMP, FlpA, and CmeC recombinant proteins of *C. jejuni*	*In vitro*/*in cellulo*; immunofluorescence, Western blot, and adhesion inhibition assay; specific against CAP IgY recognize *C. jejuni* cells and recombinant CAPs; anti-CadF, MOMP, and CmeC IgY reduce adherence of *C. jejuni* to LMH cells	Al-Adwani et al. (197)
CadF, FlaA, MOMP, FlpA, and CmeC, Peb1A, JlpA recombinant proteins of *C. jejuni*	Chickens, oral administration; lack of difference between hyperimmunized egg yolk powder treated and non-treated animals	Paul et al. (198)
*C. jejuni* KC40 whole-cell lysate and hydrophobic protein fraction	Chickens, oral administration; reduced amount of cecal *C. jejuni* after administration specific IgYs	Hermans et al. (199)
4 strains of *C. jejuni* for oral inoculation; *C. jejuni* OMP extracts or formalin-inactivated 4 strains of *C. jejuni*	*In vitro*; agglutination, motility and bactericidal assays; similar agglutination and bactericidal potential of IgY obtained according to different protocols	Thibodeau et al. (200)
Oral inoculation with live *C. jejuni* mixture strains (OI); OMP proteins or formalin- inactivated *C. jejuni* strains	*In vitro*/*in vivo*, chickens; oral administration; lack of significant *in vivo* activity against *C. jejuni* cecal colonization	Garba et al. (201)
Bacterin (13 different strains of *C. jejuni*, formalin-inactivated); composition of recombinant proteins AtpA, Ef-Tu, GroEL, Tig, CheV, and LivJ	Chickens, oral administration; prophylactic activity of obtained IgYs, especially anti-bacterin, reduction in cecal *C. jejuni* after therapeutic administration of IgYs	Vandeputte et al. (202)
Enterobactin conjugate (Ent-KLH)	Chickens, oral administration; administration of anti-Ent IgY did not confer protection against *C. jejuni* colonization	Wang et al. (203) Zeng et al. (204)

If not specified in the Activity/Properties column, then FCA/FIA was used as an adjuvant.

## Clostridium difficile

*Clostridium difficile* poses health and life hazard, especially in immunocompromised individuals. Infections caused by this pathogen are often associated with antibiotic/hospital treatment and can result in diarrhea and colitis. The growing antibiotic resistance of *C. difficile* creates therapeutic difficulties, and its ability to form spores increases the chances of transmission. Alternative treatment options include small–molecular weight antimicrobials, fecal microbiota transplantation, phage therapy, or antibody therapy (288). The development of both mammalian and avian antibodies is largely focused on targeting toxins of *C. difficile* as antigens. The recombinant C-terminal fragment of toxin A, one of the main multidomain *C. difficile* toxins, was the subject of extended study focused not only in the creation of specific IgY antibodies but also in the application of the antibodies prepared as IgY-loaded microbeads coated with pH sensitive material as a colonic-specific delivery system. The study also provided the experimental data showing the extended pH along with thermal and long-term storage stability of such a formulation. The *in vivo* release analysis of IgY from microbeads in the digestive tract showed that most IgY could be detected in the colon with a maximum IgY activity of 87.5% detected 8 h after administration (289). A similar analysis of the activity and stability of anti-toxin B IgYs was a part of the patent description (290). In a subsequent work, the toxin A and toxin B C-terminal fragment served as an immunogen prepared in the form of fusion protein. The activity of the resultant antibodies was confirmed in the rabbit red blood cell aggregation assay (291, 292). Optimized in the course of this study, the chitosan-Ca pectinate microbeads provide a higher IgY load (up to 50%) as compared to the results from previous approach (21%) (289) and efficient colon-specific antibody release (up to almost 73%) in the rat’s digestive system (291, 292).

Vaccines based on three *C. difficile colonization factors*, containing flagellar cap protein (FliD), flagellar structural protein (FliC), and cell wall–associated cysteine protease (Cwp84), each prepared as a recombinant protein, were used for hen immunization. As for the antibodies, the *in vitro* experiment showed that anti-FliD IgY inhibited *C. difficile* adhesion to human colon–derived cells more efficiently than other prepared antibodies. On the basis of this result, the anti-FliD IgYs were tested *in vivo* on *C. difficile* spore-challenged hamsters: A significant increase in animal survival proved the protective effect of specific IgY antibodies resulting most likely from the inhibition of pathogen colonization (205). Even more details regarding the *in vivo* activity of the three specific antibody preparations can be found in the patent description showing that the survival rate on the ninth day of the experiment in the group receiving anti-Cwp84 IgY and a mixture of all three antibodies was 25% (both groups), 37.5% in the group treated with anti-FliD IgY alone, and 70% in the group receiving the specific anti-FliD antibody prepared in egg yolk. The group receiving only egg yolk showed a 12.5% survival rate (293). These results indicate how important IgY formulation is when administered orally, all the more so as in the case of *C. difficile* infections the target site of IgY activity is the colon.

Guajardo and co-workers employed paraformaldehyde-deactivated *C. difficile* spores as immunogen for hen vaccination. The antibodies produced in this manner were able to cross-react with spores of different strains and, in some cases, also with *C. difficile* vegetative cell antigens. The neutralization of spores by specific IgY antibodies resulted in the inhibition of spores adherence to Caco-2 cells *in vitro*. Administration of spore-specific antibodies alone or in combination with vancomycin as a treatment of inoculated mice allowed delayed initiation of *C. difficile* infection by 1.5 and 2 days, respectively (206). The avian antibody generated with formaldehyde-inactivated *C. difficile* cells was also produced and presented in patent description together with an *in vitro* study of the inhibition of *C. difficile* adhesion to the surface of porcine intestinal epithelial cells (IPECs). After 2-h preincubation of *C. difficile* with immunized hens’ dried egg mélange (rehydrated), immunized hens’ serum, culture medium, or fraction of egg yolks from non-immunized hens, bacterial cells were incubated with IPEC for 48 h. The calculated colony forming unit (CFU)/ml showed a significant adherence decrease in treated groups with results for control groups standing at 2,300 and 4,000, for non-immunized groups 1,920 and 860, 10% egg melange groups 150 and 550, and 10% serum groups 60 and 0 (294).The combination therapy including specific egg yolk antibody was presented in case study examples in the account accompanying the invention by Borody describing the therapeutic effect of treatment of *C. difficile* infections with specific antibodies and strains of probiotic bacteria *Lactobacillus rhamnosus* or *Bifidobacterium.* The effectiveness of combined therapy was presented in two case studies of patients with chronic diarrhea and diagnosed *C. difficile* infections. In both cases, the egg yolk antibodies were able to reduce the symptoms, but only the inclusion of a probiotic into the therapy allowed for complete eradication of the infection (207). For a summary of IgY studies, see the **Table 9**.

**Table 9:** Studies concerning the production and application of IgY antibodies specific to *C. difficile*.

Immunogen	Activity/Properties	Reference
Recombinant FliD protein	*In vivo*, hamsters; 0.5 mg of IgY per day for 10 days; increased animals survival	Murvey et al. (205)
Inactivated *C. difficile* spores (paraformaldehyde)	*In vivo*, murine model; IgY of 100, 200, and 600 µg, pre- and post-inoculation and for 3 following days, IgY of 600 µg + vancomycin of 50 mg/kg, days 3–9 delayed signs of infection	Pizzaro-Guajardo et al. (206)
*C. difficile* specific	*In vivo*, human case study; 10 g/day combine therapy with probiotic bacteria; infection clearance; no data specified concerning adjuvant	Borody (207)

If not specified in the Activity/Properties column, then FCA/FIA was used as an adjuvant.

## Peridontal pathogens

Dental health is most commonly threatened by periodontal disease and tooth decay. According to Centers for Disease Control andPrevention (CDC), gingivitis and its more advanced stage periodontitis threaten the 47.2% of U.S. adults aged 30 and older with disease progression increasing with age. The main cause of periodontal disease is infection of the tissues adjacent to the teeth by bacteria such as *Streptococcus mutans*, *Fusobacterium nucleatum*, *Porphyromonas gingivalis*, and *Solobacterium moorei*. Human periodontitis (aggressive and chronic) started to be treated by systemic antibiotics though their selection and administration remain unsettled (295, 296). Pathogens responsible for periodontitis vary in their susceptibility and resistance to antibiotics; therefore, the role of microbiological profiling is extremely important. Currently, the periodontal therapy focuses on the restoration of homeostasis and balance in oral microbiota (297). In this regard, IgY antibodies can offer promising possibilities in achieving this goal.

## Streptococcus mutans

*S. mutans* occurs naturally in dental plaque. Since mid-1960s, it is regarded as a primary etiologic factor in dental caries. Its adaptations to living in this region include its ability to synthesize glucan from sucrose, which facilitates colonization, acidogenicity, and aciduricity (298). The interaction between streptococcal surface adhesins and receptors on the salivary pellicle initiates the cariogenic process. Subsequently, the synthesis of glucans contributes multiple binding sites for glucan binding proteins (GBPs) that are linked with the bacterial cell wall (211). The occurrence of dental caries due to the more and more advanced prevention is limited; it can be, however, a serious problem for people suffering from hyposalivation, for patients with head and deck tumor after radiation, people with Sjögren’s syndrome; it can also be triggered by pharmaceuticals (212). For the last 40 years, research has been conducted toward the development of a *S. mutans* vaccine. Its aim was to induce the production of secretory IgA antibodies that could reduce *S. mutans* accumulation on the tooth surface. Despite many efforts, animal studies and clinical trials there is no commercially available vaccine yet (299). There are also clinical trials that test drugs or supplements such as chlorhexidine and/or licorice mouthwash (*Efficacy of Licorice on Reducing Salivary Streptococcus Mutans Versus Chlorohexidine in Caries Risk Patients*. ClinicalTrials.gov identifier: NCT03590977. Updated 18 July 2018. Accessed 10 February 2023; *Changes in Streptococcus Mutans Colonization With Different Oral Hygiene Protocols in Adult Patients With Fixed Orthodontic Appliance*. ClinicalTrials.gov identifier: NCT05016713. Updated 9 February 2023. Accessed 10 February 2023), probiotics (*Probiotic Lozenge Reduce Streptococcus Mutans in Plaque in Orthodontic Bracket Patients*. ClinicalTrials.gov identifier: NCT02357771. Updated 23 September 2015. Accessed 10 February 2023), or natural products such as xylitol (*Prevention of Transmission of Bacteria That Cause Cavities From Mothers to Their Children*. ClinicalTrials.gov identifier: NCT00066040. Updated 3 November 2022. Accessed 10 February 2023), and ginger and cinnamon (*Cinnamon and Ginger in Comparison to Chlorhexidine Gluconate 0.2% on Oral Streptococcus Mutans*. ClinicalTrials.gov identifier: NCT03061916. Updated 23 February 2017. Accessed 10 February 2023).

In the 1990s, passive immunization with IgY antibodies specific to *S. mutans* began to be investigated (300–302). The first attempt to use particular GBP (GBP-B) as immunogen was made by Smith et al. In two experiments with a different IgY content, they examined if anti–GBP-B IgYs could act protectively against *S. mutans* accumulation in the oral cavity of rodents. There were conducted two experiments with various amount of IgY in the animal diet and duration (9 and 24 days) of the supplementation. Food was enriched with 0.17% of the total diet weight in the first experiment and 0.44% (first 9 days) and 0.3% (next 14 days) of IgY in the second. Drinking water was supplemented with IgY (75 μg/ml). At the end of the experiment (day 78), rats were colonized with *S. mutans*; however, there were significant differences between group treated with specific and control IgYs. It is worthy to emphasize that no dietary supplements were given during the last 54 days of the experiment. The results indicated the protective effect of anti–GBP-B IgY that could suppress the accumulation of *S. mutans* in the oral cavity of rats and provide protection from dental caries (211).

A different approach to obtaining antibodies capable of dental caries prevention was chosen by Hamada et al. (303) and Krüger et al. (212). They applied cell-associated (CA) glucosyltransferases (gtf) as an immunogen for hens. Gtfs are considered a major virulence factor of *S. mutans* in the dental caries pathogenesis. Because *S. mutans* accumulation is most troublesome for individuals with hyposalivation, Krüger et al. decided to evaluate the anti-streptococcal potency of anti–CA-gtf IgY in a desalivated rat model mimicking the clinical situation of the most affected patients. They conducted animal experiments on the basis of the initial results that showed that specific IgY can reduce the aggregation of *S. mutans* and inhibit GftB and GtfC not only in solution as tested by Hamada et al. (303) but also, to a lesser extent, when bound to saliva coated hydroxyapatite beads. As a result, caries was significantly reduced in the group treated with anti–CA-gtf IgY (10 mg/ml) (212). The potency of anti–CA-gtf IgY to reduce *S. mutans* adherence and forming dental plaque in the human oral cavity was evaluated by Nguyen et al. Dental students [99 healthy men (n = 76) and women (n = 23) at the average age of 23] were subjected to lozenges (72 mg of IgY per pill; slow dissolving by sucking) with specific anti-gtf IgY or a placebo. The daily intake of lozenges with specific IgY reduced *S. mutans* colonization, and, moreover, almost 35% of the post-trial saliva samples in the group treated with specific IgY had no colonies of *S. mutans* (215).

The persistency of IgY antibodies in saliva and their influence on dental biofilm formation in combination with chitosan in soybean milk in malnourished rats was evaluated. Among different groups (fed only with soy milk, soy milk and chitosan, soy milk and anti–*S. mutans* IgY, and soy milk with anti–*S. mutans* IgY and chitosan), the group that received soy milk supplemented with chitosan [antibacterial agent (304)] and anti–*S. mutans* IgY revealed the lowest colonization (213). In subsequent research, Bachtiar et al. applied gel enriched with specific anti–*S. mutans* IgY (2% specific IgY) on rat teeth, which resulted in a reduced *S. mutans* quantity on the tooth surface. The formulation of the water-based carboxymethyl cellulose gel was stable for 30 days at room temperature. Additional components of the gel did not affect the IgY activity (214). Bachtiar et al. also verified the possibility of disturbing communication between autoinducer molecules and their receptors that could influence the *S. mutans* biofilm formation. For this purpose, anti-ComD IgY antibodies were obtained and verified for immunoreactivity against *S. mutans* isolates from patients with and without dental caries. They also revealed in a bacterial *in vitro* assay that 0.14% solution of specific anti-ComD IgY is able to inhibit the biofilm formation of *S. mutans* (208).

Jain et al. evaluated the influence of chewable tablets containing xylitol and IgY antibodies on salivary *S. mutans* in nearly 150 children. Tablets were enriched with 20 mg (“after breakfast pill”) and 40 mg (“before bed” pill) of IgY and were applied for 15 days. This passive therapy decreased *S. mutans* colonies and conferred prolonged protection against recolonization (216). Nanostructured antimicrobial material functionalized with anti–*S. mutans* IgY was applied by Chen et al. They synthesized structures of hydroxyapatite (nanosheet-assembled or nanorod-assembled) that were further modified with anti–*S. mutans* IgY. The bacterial kinetics assay revealed high antimicrobial potential against *S. mutans* (nearly 100% of the initial bacteria were killed after 24 h) (209).

Another common dental ailment is dentin hypersensitivity caused by the exposure of dentinal tubules. Because of the enamel and dentil defects, dentin is susceptible to *S. mutans*–induced dental caries. Occlusion that reduces pain is among the accepted types of treatment. Yan et al. assumed that biocompatible material that could physically protect the tubules and, in parallel, decrease *S. mutans* colonization might reduce pain and desensitize the dentinal tubules. In their research, amorphous calcium phosphate was loaded with IgY (IgY@ACP). Its activity was evaluated with the use of dentin disks and *in vivo* on rat incisors. After treating with IgY@ACP, dentinal tubules were occluded by mineralized hydroxyapatite-like layer. Adhesion rate of *S. mutans* was significantly reduced. The *in vivo* effect needs to be further evaluated (210).

The application of IgY antibody produced with the CA glucosyltransferase fraction isolated from *S. mutans* strains of different serotypes is an early description of patented inventions. Antibody activity was confirmed by ELISA and the adherence inhibition test, and IgY was used as active components of toothpaste and mouth wash (305, 306). In the following years, other patented toothpaste compositions utilized IgY antibodies specific toward water-soluble and insoluble glucosyltransferase and other *S. mutans* antigens, including whole cells (307), or inactivated whole cells mixed with the soluble protein fraction (308). In the alternative approach, the immunogen applied for vaccination was the mixture of *S. mutans* serotype C and D in Freund’s adjuvant. The derived IgY antibodies were patented as active ingredients of functional food and hygiene products such as milk/milk powder, chewing gum, and toothpaste (309). In another patented example, IgY antibodies produced with the C and D serotypes of *S. mutans* served as an anti-caries ingredient of chewable tablets. It proved to be effective in pain reduction (86.73%) (217). The patented oral sprays, three different preparations containing the IgY antibody specific for the serotypes mentioned above as active ingredients (0.1%–5.0%) were applied to humans, among them children aged 2–5. The spray was used in children three times a day for two months, with the subsequent examination after 1 year. The caries surface in the treated group was significantly decreased compared to the non-treated group, but among the preparations of the three tested sprays the best results were achieved for the spray that also contained triclosan, which also has anti-*S mutans* activity. In adults, the spray was tested in patients with bleeding gums and oral ulcers. The oral spray was used in the same manner for 1 month with a statistical reduction in the symptoms of the disease at the end of the study (218).

Microcapsules that were added as an anticaries component to toothpaste were made from immunoglobulin Y produced with only serotype C of *S. mutans*. Volunteers brushed their teeth with this substance twice a day for 3–4 weeks. So much was enough to significantly reduce the percentage of *Streptococcus mutans* in saliva (when compared to the total of anaerobic bacteria). Antibody-containing microcapsules did not show any signs of toxicity when tested in rats and were characterized by good stability and activity in the toothpaste formulation (tested up to 120 days) (219). For a summary of IgY studies, see the **Table 10**.

**Table 10:** Studies concerning the production and application of IgY antibodies specific to *Streptococcus mutans*.

Immunogen	Activity/Properties	Reference
ComD DNA coding region isolated from *E. coli* Dh5α	*In vitro*; ELISA, SDS-PAGE, bacterial culture, and biofilm assay; anti-ComD IgY reveal immunoreactivity against two different strains *S. mutans*, reduction of biofilm formation; no specified data concerning adjuvant	Bachtiar et al. (208)
*S. mutans*	*In vitro*; bactericidal kinetics; high antimicrobial activity, no specified data concerning adjuvant	Chen et al. (209)
Commercial IgY Biological Technology, Hangzhou, China	*In vitro*, rat model; significantly reduced adhesion rate in dentinal tubules	Yan et al. (210)
GBP-B protein purified from *S. mutans* SJ strain	Rat model; oral administration (food and water); anti–GBP-B IgY can inhibit accumulation of *S. mutans* in rat oral cavity	Smith et al. (211)
CA-gtf purified from *S. mutans* MT8148	Desalivated rat model; administration with drinking water passive administration of IgY anti–CA-gtf demonstrates the prophylactic effect and reduce the progression of dental caries	Krüger et al. (212)
No specified data	Malnourished rats; reduction of *S. mutans* biofilm after feeding with soybean milk supplemented with *S. mutans*–specific IgY and chitosan	Bachtiar et al. (213)
Obtained by Institute of Agriculture in Bogor	Rat caries model; topical administration; curative effect of the specific IgY-enriched gel	Bachtiar et al. (214)
CA-gtf purified from *S. mutans*	Human trial; lozenges oral administration; anti–CA-gtf IgY suppressed *S. mutans* colonization; oil-in-water emulsion adjuvant	Nguyen et al. (215)
No specified data	Human trial; chewing tablets for children; reduction of *S. mutans* biofilm, prolonged protection against recolonization	Jain et al. (216)
Inactivated *S. mutans* serotypes C and D	Human; tablet after each meal and next 3–4 h after/7days; reduction of pain	An et al. (217)
Inactivated *S. mutans* serotypes C and D	Human; three times a day over 1 or 2 months; reduction of gum bleeding and oral ulcers (1 month treatment, adults); reduction of caries surface (children, 2 month treatment); no specified data concerning adjuvant	Zhao (218)
Inactivated *S. mutans* serotypes C	Human; brushing teeth twice a day 3–4 weeks; inhibitory effect on *S. mutans*	Zhihai (219)

If not specified in the Activity/Properties column, then FCA/FIA was used as an adjuvant.

## Fusobacterium nucleatum

*F. nucelatum*, gram-negative anaerobic bacteria are another microorganism that is strongly associated with oral diseases (periodontitis and halitosis) and extraoral infections (gastrointestinal and urinary tract). As microflora changes during plaque formation from gram positive (*S. mutans*) to gram negative, *F. nucelatum* colonization increases (220). Interestingly, *F. nucleatum* can have a symbiotic relationship with the host. *F. nucleatum* DNA/RNA was also present in nucleic acid samples from colorectal cancer tissue and oral cancer (310, 311). Alkharaan et al. also found a correlation between circulating and salivary antibodies against *F. nucleatum* and pancreatic cancer (312), which is brought about by *F. nucleatum’s* capacity to invade different cell types such as epithelial, endothelial, and fibroblasts (311). Search for new treatment against *F. nucleatum* is ongoing. Some of the approaches include vaccines (313), plant extracts (314), probiotics (315), photodynamic therapy (316), and passive immunotherapy.

Anti–*F. nucleatum* IgY antibodies were described for the first time by Xu et al. IgY antibodies obtained after *F. nucleatum* immunization were able not only to specifically recognize bacterial cells but also to inhibit biofilm formation. *In vivo* studies performed for the evaluation of specific IgY (2 mg per animal) inhibitory activity on alveolar bone loss in mice that is a common symptom of infection revealed a significant reduction in bone loss compared with control animals (220). Similar results were obtained by Wang et al. By the application of halitosis and periodontitis rat model, they were able to evaluate the effectiveness of specific IgYs (40 mg/ml) administrated in oral cavities as an agent against bone loss. The ELISA assay revealed that the level of anti-inflammatory cytokines, IL-6, and TNF-α was reduced after 4 weeks of IgY administration (221). For a summary of IgY studies, see the **Table 11**.

**Table 11:** Studies concerning the production and application of IgY antibodies specific to *Fusobacterium nucleatum*.

Immunogen	Activity/Properties	Reference
Formaldehyde-fixed *F. nucleatum* (JCM 11024)	*In vitro* and mice model; oral administration; inhibition of *F. nucleatum* growth and biofilm formation, decrease in alveolar bone loss	Xu et al. (220)
IgY against F. nucleatum obtained by Maxam Ltd. (China)	*In vitro* and halitosis and periodontitis rat model; reduced alveolar bone loss, decreased level of IL-6 and TNF-α	Wang et al. (221)

If not specified in the Activity/Properties column, then FCA/FIA was used as an adjuvant.

## Porphyromonas gingivalis

The important role in pathogenesis of periodontitis is played by gram-negative anaerobic *P. gingivalis*, which requires for its growth protoheme (317). *P. gingivalis* acquires the hemagglutinin (responsible for cell adherence) and hemolytic (lyse of erythrocytes) activity (223). In addition, bacteria secrete factors that destroy periodontal tissues: gingival protease, LPSs, indole, and organic acids. After secretion of these virulence factors, inflammatory mediators are released and the dysregulation of the host’s immune system occurs (318). Scaling and root planing performed with the application of antibiotics and antimicrobial agents such as chlorhexidine mouthwash is the classic approach to reduce periodontitis and *P. gingivalis* colonization (228, 319). Alternative anti–*P. gingivalis* therapies include passive immunization with monoclonal antibodies (320), photodynamic therapy (321), and combining new chemicals with traditional antibiotics such as colloidal bismuth with metronidazole (322). Another approach is the use of specific IgY antibodies that could be applied as passive immunization agents against *P. gingivalis*.

Li et al. decided to combine *P. gingivalis* and *Aggregatibacter actinomycetemcomitans* also considered a pathogen responsible for chronic periodontitis to achieve immunogen. This is the first example of IgY with such “dual” reactivity against periodontitis. The resultant antibodies (2–8 mg/ml) inhibited biofilm formation (222). Tezuka et al. focused on the prevention of the adherence of *P. gingivalis* to mucosa. As the molecular target for producing IgY antibodies, they chose 122k-HagA protein, which is a fusion protein composed of a 80-kDa HagA (hemagglutinin A) with the PVQNLT functional motive and a 42-kDa maltose-binding protein. Because the PVQNLT motif is known to be a common feature of hemagglutinin molecules, the large capacity of IgY obtained by Tezuka et al. to neutralize hemagglutinin activity is dictated by this region (223). Hamajima et al. choose as an immunogen the coaggregation factor, conserved among *P. gingivalis* OMP. Generated antibodies inhibited the coaggregation of *P. gingivalis* with *Streptococcus gordoni*, which are considered to be an early colonizer of the oral cavity (224, 323).

Gingipains are regarded as modulators of *P. gingivalis* attachment to the cells and one of the most important pathogenic factors of *P. gingivalis*. They belong to the cysteine proteases family and are located in the outer membranes, vesicles, and extracellular structures. Their enzymatic activity disrupts cytokines and complement system components as well as downregulates host inflammatory response (225, 324). Therefore, gingipains can effectively serve as immunogens for the production of specific IgY antibodies with therapeutic potential. Yokoyama et al. obtained gingipains specific IgY that was a potent inhibitor of gingipains enzymatic activity and additionally pretreatment of gingipains with specific IgY (50 mg/ml) inhibited gingipains-induced detachment of epithelial cells in *in vitro* assay (225). In their further research, they evaluated the activity of anti-gingipains IgY potential among five patients with a detectable levels of *P. gingivalis* colonization. The specific IgY as an ointment containing 20–30 mg of 20% IgY was administered simultaneously with scaling and root planing. The level of *P. gingivalis* measured by real-time PCR in the group of patients treated with specific antibodies decreased and was sustained for 4 weeks after treatment (226).

IgY antibodies specific to gingipains were also used in research concerning passive immunotherapy against biofilm formation in cats. After 40 days of a diet enriched with specific IgY (around 213 mg of IgY/cat/day), the analyzed parameters such as plaque formation, dental calculus, gingivitis index, and percentage of *Porphyromonas gingivalis* of the oral cavity were evaluated. The plaque index was reduced when compared to the control group (325).

The efficacy of lozenges containing anti-gingipains IgY was the subject of a clinical trial (*Evaluation of IgY Antibody Effectiveness in Supportive Therapy of Periodontitis Patients*. ClinicalTrials.gov identifier: NCT02705885. Updated 11 March 2016. Accessed 13 February 2023). Lozenges were administered supportively to scaling and root planing as a food supplement. The test group received lozenges with specific anti-gingipains IgY (12 mg/lozenge), whereas the control group received lozenges with sham-immune IgY (placebo). After 8 weeks of treatment, the tested parameters improved in both groups—a significant reduction in the gingival bleeding index and probing pocket depth. The authors point out that it would be worth looking at the effects without prior scaling and root planing procedures (227).

In another clinical, trial Xu et al. experimented with IgY antibodies characterized by a different specificity and different method of antibody delivery. Patients with moderate to severe chronic periodontitis were divided into three groups receiving anti–*P. gingivalis* IgY (whole cell) mouthwash (antibody titer, 1:1,280), 0.2% chlorhexidine mouthwash, and placebo mouthwash (sterile water, glycerin, sorbitol, citric acid, and sodium citrate). Patients rinsed their mouths with three times a day for 1 min for 4 weeks. The use of anti–*P. gingivalis* IgY compounded with scaling and root planing revealed a decrease in probing depth and increase of clinical attachment level. The level of *P. gingivalis* was reduced in all groups tested, in contrast to what Yokoyama et al. had found (226). The bleeding symptom on probing and the plaque index results were changed by the same degree in both groups, with anti–*P. gingivalis* IgY and chlorhexidine. The results concerning level of red complex bacteria revealed significant reduction when compared with the placebo group (228).

The patented solutions that describe the applications of anti–*P. gingivalis* IgY antibodies are focused more on specific formulations than on different immunogen/specificity analysis. The vaccines used for the generation of specific antibodies utilize inactivated whole cells or cell lysate as an immunogen. Among the inventions described, there is a toothpaste containing between 0.5% and 5.0% of anti–*P. gingivalis* IgY with the dose-dependent anti-bad breath effect observed. The suppression of volatile sulfide production by *P. gingivalis* (causing a bad breath problem) by IgY was verified *in vitro* in artificial saliva acting as a culture medium (326). The result showed that, after 8 h of the experiment, the volatile sulfide production was reduced to 12.75% (IgY groups) of the non-treated cultures. The use of IgY gave superior results compared with those of the metronidazole-treated culture.

In another invention, IgY antibody was derived from hen immunization with lysed cell soluble protein fraction and tested in the chronic periodontitis model in rats. The groups were observed for 4 weeks, and, in the specific IgY-treated group, a significant reduction in the hemorrhage index and the plaque index decrease were observed compared to the control (229). A similar *in vivo* study was performed in a periodontitis rat model to verify the action of anti–*P. gingivalis* IgY encapsulated in liposomes. These experiments confirmed the good clinical value of such preparations (230). In another invention, several food products with anti–*P. gingivalis* IgY antibody as an active ingredient (0.5%) including chewing gum, candies, and chocolate were presented. Among the confectionery tested, the chocolate and chewing gum preparation sustained antibody activity. The latter showed a higher degree of antibacterial activity and a reduction in bacteria adhering to the surface of the teeth, which were verified in human subjects (327). Most of the patented inventions utilizing anti–*P. gingivalis* IgY apply whole cells or lysate as an antigens; however, one of the early inventions on the subject also describes immunogens prepared from the fimbrial and capsular fractions and the application of resulting IgYs as an active component of a toothpaste (307). For a summary of IgY studies, see the **Table 12**.

**Table 12:** Studies concerning the production and application of IgY antibodies specific to *Porphyromonas gingivalis*.

Immunogen	Activity/Properties	Reference
Formaldehyde-fixed *P. gingivalis* and *A. actinomycetemcomitans*	*In vitro*; aggregation and inhibition of the biofilm formation by specific IgY	Li et al. (222)
Recombinant HagA protein	*In vitro*, rabbit erythrocytes; significant neutralization of the hemagglutinating activity of *P. gingivalis*	Tezuka et al. (223)
Recombinant OMP protein	*In vitro*; coaggregation assay; specific antibodies inhibited coaggregation of *P. gingivalis* with *S. gordonii*	Hamajima et al. (224)
Gingipains	*In vitro*, human epithelial cells; inhibition of gingipains enzymatic activity, inhibition of gingipains activity by specific IgYs, inhibition of gingipains-induced detachment of cells; oil adjuvant	Yokoyama et al. (225)
Gingipains	Periodontitis patients; significant reduction of *P. gingivalis* after anti-gingipains IgY treatment; oil adjuvant	Yokoyama et al. (226)
Gingipains	Randomized controlled clinical trial; improvement in clinical parameters such as reduction of the gingival bleeding index and probing pocket depth; oil adjuvant	Nguyen et al. (227)
Commercially obtained mouthwash (Anhui Province Bioengineering Co. Ltd, Hefei, China) containing anti–*P. gingivalis* IgY	Randomized placebo-controlled clinical trial; improvement in clinical parameters such reduction red complex bacteria	Xu et al. (228)
Cells lysate soluble protein fraction	*In vivo*, rats; IgY solution of 1 mg/ml used to rinsed the periodontal pockets for 4 weeks; reduction of the hemorrhage and the plaque indexes	Jiang et al. (229)
No specified data	*In vivo*, rats; 2 mg/ml, once a week, 4 weeks; improvement in clinical symptoms of periodontitis	Xu et al. (230)

If not specified in the Activity/Properties column, then FCA/FIA was used as an adjuvant.

## Solobacterium moorei

Another microorganism tightly connected with oral cavity and intestinal track pathologies such as halitosis and oral infections is anaerobic gram-positive bacillus *Solobacterium moorei*. It is also characterized as an opportunistic pathogen that induces bloodstream and surgical wound infections (328, 329). Although it is sensitive to many antibiotics such as ampicillin, chloramphenicol, and erythromycin, an alternative therapy is worth considering due to, for example, the side effects produced by the mentioned substances and the possibility of acquiring antibiotic resistance. Antibiotic therapies are assisted by surgical debrident and drainage of pus that oxygenize affected tissues (330).

Li et al. through the immunization of hens with formaldehyde fixed cells and complete (first immunization)/incomplete (booster injections) Freund adjuvant obtained anti–*S. moorei* IgY. Antibodies were isolated and purified by water dilution followed by ammonium sulfate precipitation. After IgY characterization by ELISA and Sodium dodecyl-sulfatepolyacrylamide gel electrophoresis (SDS-PAGE), bacteria growth inhibition and biofilm formation assays were performed. Anti–*S. moorei* IgY (10 to 40 mg/ml) was able to significantly inhibit the growth and biofilm formation of *S. moorei*. Li et al. evaluated also the influence of specific IgY treatment on the *S. moorei* colonization of the oral cavity of mice. Treatment was performed by the application of 4 mg of specific IgY powder in phosphate-buffered saline supplemented with 2% carboxymethyl cellulose for 6 weeks. The amount of bacteria significantly decreased afterward (331). The invention describing the application of developed IgY antibodies contains the preparation of halitosis controlling products including mouthwash, oral spray, toothpaste, chewing gum, or lozenges containing 0.8%–1.0% of anti–*S. moorei* specific IgY (332).

## Staphylococcus aureus

According to CDC, as much as 30% of population is carrying the gram-positive *Staphylococcus aureus* without any harm. The dangerous condition resulting from *S. aureus* infection occurs when bacteria spreads to the bloodstream (bacteremia or sepsis), heart (infection of heart valves), or bones. *S. aureus* bacteremia incidence ranged from 9.3 to 65 cases/100,000/year. The seriousness of the infection is evidenced by the fact that the mortality rate for *S. aureus* infections is 30% (333).

Some of the types of *S. aureus* include MRSA (methicillin-resistant *Staphylococcus aureus*), MSSA (methicillin-susceptible *Staphylococcus aureus*), VISA (vancomycin-intermediate *Staphylococcus aureus*), or VRSA (vancomycin-resistant *Staphylococcus aureus*) (334). The antibiotic resistance of *S. aureus* is an effect of horizontal gene transfer and mutations that alter the drug binding site, target DNA gyrase, or reduce membrane proteins. Other mechanisms cause changes in outer membrane permeability or efflux systems. MRSA strains are also able to produce enzymes that can hydrolyze β-lactams such as penicillin. There is an increasing resistance of *S. aureus* infections that makes treatment more and more difficult (335, 336). Novel strategies of treatment consist of iron chelation (337), phage therapy (338), nanoparticles (339), and quorum sensing inhibition (340).

Infections caused by antibiotic-resistant *S. aureus* are very dangerous for animals. Important problems faced by dairy industry are inflammations of mammary gland (bovine mastitis). The commercially available vaccines against mastitis are focused on prophylaxis rather than on therapy and are not efficient (341). Zhen et al. assumed that IgY antibodies specific for mastitis-causing *S. aureus* strains could provide passive protection. In their *in vitro* studies, they produced anti–*S. aureus* IgYs (1 to 10 mg/ml) that were able to inhibit the growth of *S. aureus*. The inhibition of *S. aureus* growth by specific IgY of 10 mg/ml was comparable with penicillin (100 μg/ml). The antibodies (final concentration 0.1, 1, and 10 mg/ml tested, 1 mg/ml effective) also enhanced the phagocytosis of *S. aureus* by milk macrophages through the changes in the bacterial surface. Phagocytosis, apart from adhesion inhibition, is the second mechanism of fighting *S. aureus* colonization (231). Similar results concerning the inhibition of the *S. aureus* growth were achieved by Guimarães et al., with IgY antibodies (5 μg/ml) received after immunization of hens with a different strain of *S. aureus* (233).

In further experiments, Zhen et al. treated cows with clinical and experimental mastitis with previously examined IgY anti–*S. aureus* IgY. Within the framework of treatment concerning different groups of animals, first two groups received specific antibodies (20 mg/ml) or penicillin (100 mg/ml) administered as an intramammary infusion for 6 days. The third group of animals was not treated with any infusions. The bacterial count in milk revealed a significant (higher than with penicillin) reduction in *S. aureus*. In addition, the cure rate of mastitis was higher in the group with IgY treatment (83.3% and 50% in experimental and clinical mastitis, respectively) than in the group with penicillin treatment (66.7% and 33.3%, respectively) (235).

Wang et al. evaluated IgY antibodies specific to certain types of *S. aureus* strains causing mastitis—encapsulated and non-encapsulated—which were more invasive for the bovine mammary epithelial cell line (MAC-T) because of the facilitated invasion due to the reduced/absent polysaccharides on the bacterial surface. The antibodies obtained by Wang et al. were able to effectively block the internalization of the homologous strain by MAC-T cells when used at a concentration of 5 mg/ml. The growth inhibition level was, however, not satisfactory. The authors suggest that further efforts concerning anti–*S. aureus* IgY should be focused on the study of the inhibition of the internalization rather than growth inhibition (234).

Kota et al. developed IgY antibodies after immunization of hens with staphylococcal protein A, the component of the cell wall that promote *S. aureus* evasion (342). The primary use intended by the authors was diagnostic applications, but they also evaluated their ability to inhibit the growth of *S. aureus*, its proliferation and formation of the biofilm. IgY antibodies (150 μg/ml) inhibited the growth of *S. aureus* for 8 h, and, after that period, the bacteria resumed to proliferate. The authors suggest that bacteriostatic anti-SpA IgYs might be used as an additive to cosmetic creams for controlling *S. aureus* colonization (232).

There are also two reports concerning the influence of adjuvant on the immunogenicity of *S. aureus* during antibody production. Freund’s complete adjuvant and Emulsigen-D were used by Grzywa et al., with two staphylococcal proteins: extracellular fibrinogen-binding protein (Efb) and major histocompatibility complex class II analog protein (Map). The study revealed a failure in inducing a specific immune response by Emulsigen-D (343). Instead, Kubo et al. used for immunization formaldehyde-inactivated whole *S. aureus* and λ-carrageenan, Freund’s complete adjuvant, and Freund’s incomplete adjuvant. The results showed that all adjuvants increased the immunogenicity; still Freund’s complete adjuvant induced the highest level of specific IgYs (68).

The methods of genetic engineering were used for the generation of scFv to be used as agents for the study of the antimicrobial mechanisms (344). IgY antibodies are also applied as a tool for the determination of *S. aureus* infection, presence of toxins, and determination/differentiation *S. aureus* strains. There are many interesting assays such as colorimetric immunoassays, ELISA, lateral flow devices, and immunosensors that utilize specific IgY (345–354). For a summary of IgY studies, see the **Table 13**.

**Table 13:** Studies concerning the production and application of IgY antibodies specific to *Staphylococcus aureus*.

Immunogen	Activity/Properties	Reference
Formaldehyde-fixed *S. aureus* (CVCC545)	*In vitro*; growth inhibition; *S. aureus* growth inhibition and increase in phagocytosis	Zhen et al. (231)
SpA protein from *S. aureus*	*In vitro*, growth inhibition; *S. aureus* growth inhibition	Kota et al. (232)
Formaldehyde-fixed *S. aureus* (ATCC 33593)	*In vitro*; growth inhibition; high inhibitory capacity of anti–*S. aureus* IgY; alhydrogel adjuvant	Guimarães et al. (233)
Formaldehyde-fixed *S. aureus* strains (ATCC 49521, 49525, 55804 (non-encapsulated))	Bovine mammary epithelial cell line; significant reduction of intracellular *S. aureus* in bovine mammary epithelial cells	Wang et al. (234)
Formaldehyde-fixed *S. aureus* (CVCC545)	Cows with experimental and clinical mastitis; intramammary infusion, high rate of the cure in experimental and clinical mastitis	Zhen et al. (235)

If not specified in the Activity/Properties column, then FCA/FIA was used as an adjuvant.

## IgY compositions and applications

Patent databases show an increasing number of records regarding the production and application of hyperimmune IgYs generated either through hen immunization with antigens mixture or, to a lesser extent, cocktails composed of different single-antigen–derived IgYs. Such an approach is clear because proposing a new set of therapeutically important bacteria to be used as antigens for IgYs development provides the required element of novelty and ensures priority rights. In addition, the targeted environment of the body where the antibodies are intended to be used is usually colonized by multiple bacteria species; thus, the IgY-based product of broad specificity might display higher therapeutic or prophylactic potential. One more argument that should not be overlooked is that increasing the range of IgYs specificity in the product does not increase the risk of toxicity, intolerance, or other adverse effects, which is not necessarily true in regards to classical drug mixtures. In fact, apart from egg protein intolerance, which is scarcely observed in the case of purified IgYs (355), the cross-reactivity with the healthy microbiome may be a potential concern. However, *in vivo* studies showed the positive effect of hyperimmune IgYs on the microbiome and immune status (356–358). In most cases, the patented inventions use inactivated whole cells or cell lysates as a source of antigens and provide examples of specific areas of application or even specific IgY-based products. The selected examples of products containing hyperimmune IgYs presented below are divided into categories on the basis of their target place of action. The examples include production of IgYs with at least two different bacteria species used as antigens. IgYs specific toward different strains of one bacterium were excluded. Although patents concerning the non-bacterial antigens were not included below, few interesting examples were provided (e.g., commercially available products; see section IgY market). Patents based on the application of antibodies obtained from external sources were not analyzed. Patents describing products (also IgYs with multiple specificities), which are commercially available, are included in section IgY market.

Among patents describing hyperimmune avian antibodies for human use the most common are digestive tract or mouth/teeth-directed IgYs. Several interesting inventions cover the atypical therapeutic potential of IgYs. The patent of Butz et al. along with clinical trials (IgNova GmbH, 2017-2018, *Efficacy and Safety of IGN-ES001 in Chronic Widespread Pain With or Without Fibromyalgia*, NCT03058224. Updated 7 August 2018. Accessed 2 March 2023) assesses how specific IgY supplementation influences the quality of life of patients with chronic widespread pain (with or without fibromyalgia) (359). As widespread chronic pain/fibromyalgia is difficult to diagnose and the underlying mechanisms and therapeutic options are not known, the impact of diet is considered as possible treatment. The patented pharmaceutical composition of IgYs specific to *S. typhimurium* and *E. coli* (360) was tested in a randomized, double-blind, placebo-controlled trial for 6 weeks with the daily dose of 2 g of specific (tested) or unspecific (placebo) IgYs. The results of the study showed an average (yet significant and distinguishable from placebo) but a promising improvement in the assessment of symptoms such as general pain, comorbidity, high disease activity, migraine, or irritable bowel syndrome. During the course of a trial, patients from both groups reported a similar number of mild adverse effects (e.g., headache) but no serious adverse events (359).

More common application of IgYs in the context of targeted disease was the subject of inventions describing the use of avian antibodies combined with bovine colostrum for the treatment of diarrhea (361, 362). From many antigen-specific IgYs generated individually, the mixture of antibodies with direct specificity toward rotavirus, *Escherichia coli* (ETEC), *E. coli (Shiga toxin-positive*) and *Salmonella*, and indirect specificity to *E. coli* (EIEC) and norovirus (*in vitro* neutralization) was selected and tested in children (University of Colorado, Denver, 2015-2017, *Impact of the Nutritional Product PTM202 on Acute and Long-Term Recovery From Childhood Diarrheal Disease*, NCT02385773. Updated 6 January 2017. Accessed 3 March 2023). The study was carried out in 324 subjects (aged 6 to 36 months) with acute diarrhea. The children were divided into treated and placebo groups. The 7 of g dose of the product containing dried colostrum and whole eggs obtained from immunized hens was administered once a day for three days (additional treatment, for example, antibiotic, was prescribed if necessary). The results demonstrated the effectiveness of the therapy only in patients infected with at least one targeted pathogen (direct or indirect). This observation is a strong indication that the immunoglobulins Y present in the composition play a crucial therapeutic role, although colostrum also contains protective IgGs (363). The mentioned above patent holder (PanTheryx, Inc.) also manufactures a food supplements line called DiaResQ^®^ for children and adults providing relief from diarrhea. The specific activity of the yolk antibodies used in these products is not listed; however, non-immune egg yolks contain bacteria-directed IgYs (due to normal animal contacts with bacterial antigens), which are also relevant in the context of intestinal tract diseases) (362, 364).

Several patents described bacterial antigens-directed IgY-based functional food products helpful in combating pathogens causing diarrhea and enteric infections including ice cream, yogurt (365, 366), and mayonnaise (produced with hyperimmune eggs) (366), kimchi (367), soy sauce (368), soybean paste (369), fruit juice (370), or milk powder for children (371). However, the functional activity/stability of IgYs in those products has not been analyzed thoroughly.

An interesting application of antibacterial IgYs as a component of food products is the composition of avian antibodies specific to several bacteria species responsible for the deterioration of processed meat products. Twelve bacteria species were selected and used separately for hen immunization. The analysis of IgYs activity showed that, for most antigens (except *S. epidermidis* and *S. typhimurium*), the animals responded in the production of antibodies with high titer. Interestingly, the lower titer IgYs also displayed a growth-inhibitory effect on target bacteria when tested *in vitro* (90% *S. epidermidis* and *S. typhimurium* and 99% *E. coli*). The mixture of antibodies added to sausages (0.5%) maintained their antibacterial effect for 21 days of storage at 20°C, highlighting the potential of IgYs to replace currently used food additives ensuring the safety of products, mainly processed meat products that are not completely pasteurized (372).

IgY preparations and IgY containing products designed to fight mouth infections such as periodontitis and caries are one of the most frequently patented utilizing mixture of hyperimmune IgYs specific toward different species of which the main are *Streptococcus mutans and Porphyromonas gingivalis*. The most obvious solution for teeth/gum infections is IgY-containing toothpaste. The invention utilizing hyperimmune IgYs with anti-carriers, anti-periodontal disease, or anti-bad breath activity was proposed by Paau et al. The product was tested in human subjects for nine weeks (used twice a day), and samples (saliva and plaque) were taken once a week. The dental caries prevention effect was observed as a significant reduction of *S. mutans* presence in the total number of anaerobic bacteria, with 20.05% in saliva and 13.96% in plaque (after 8 weeks) in the experimental group, whereas the control group was essentially unchanged (approximately 50%). The inhibitory potential of IgYs against pathogens causing periodontitis evaluated with samples taken from the periodontal area showed a decreased number of bacteria in the experimental group (40% reduction after four weeks and 62% in the eighth week), whereas, in the control group, the pathogen number was approximately 90% of the initial number (for the whole experimental time) (373). Two other inventions used fewer antigens (*S. mutans*, *S. sobrinus*, *F. nucleatum*, and *P. gingivalis*), but several approaches for the optimal IgY manufacturing procedure (immunization with mixes of four or two antigens at a time, whole cells alone or combined with cell fragments) were described. The patented toothpaste compositions containing anti-caries or anti-halitosis IgYs showed an antibacterial and anti-adhesive effect *in vitro* (374, 375). Another group of products is antibacterial mouthwash containing IgYs. Mouthwash formulated with avian antibodies specific to *P. gingivalis*, *F. nucleatum*, and *S. moorei* was developed for the prevention and treatment of bad breath. The regular application of the product (four times a day for 5 min) removed the bad breath problem (376). Other preparations of IgYs with confirmed *in vitro* antibacterial activity and specificity toward *P. gingivalis* and *F. nucleatum* were used as an active ingredient of the mouthwash product (377). Different methods for the oral application of antibacterial IgYs are spray preparations. In one of such inventions, not only oral spray but also lozenge and chewing gum that utilize IgY antibodies specific to *S. mutans*, *P. gingivalis*, and *H. pylori* were presented (378). Another patent application described the oral spray prepared with anti–*P. gingivalis* and anti*–A. actinomycete* IgYs with *in vitro* confirmed bacteriostatic effect and capacity for binding to the target pathogens and reduction of biofilm formation (379). More universal set of target antigens was used for the preparation of IgY-containing chewing gum (targeting mouth and throat pathogens). The product tested in humans, after a meal three times a day for 5 days, provided reduced gum bleeding and inflammation, periodontal congestion, edema, or bad breath (380).

One of the less standard applications is the utilization of IgYs as the active components of tooth coating composite. The composite was designed to be applied as a film on the surface of the teeth. As the invention should provide antibacterial functionality, several solutions with different active antibacterial substances were tested including IgY antibodies. The functional effectiveness of preparations that contained specific avian immunoglobulins was analyzed *in vivo* in rats. Two months after composite application, an average caries score revealed that the addition of antibodies into the composite reduced the number of pathogens present not only on the treated teeth but also on neighboring sites and cheeks. Furthermore, the effect was dose-dependent, and the total score was reduced from 104.9 ± 1.9 (composite without IgY) to 71.1 ± 4.1 (6% IgY) and to 42.8 ± 2.8 (20% IgY). The antiperiodontal effect was tested in children (case study) with the observed reduction (to 60%) of the relative number of bacteria (compared to the initial number) within 2 weeks after the composite was applied (381). Another possible method of administration of anti-caries and anti-periodontal IgYs, especially in the case of very small children, is their addition into an infant powder milk. Sheng et al. in their research identified most common infectious pathogens, selected potential immunogens, and immunized birds (**Table 14**). After IgY isolation and purification from egg yolks, obtained IgYs were mixed with milk/milk powder in a particular ratio to obtain immune milk (382).

**Table 14:** Hyperimmune IgYs—immunogens preparation and application of products.

IgY Specificity	Vaccine Preparation	Application	Reference
Digestive tract			
*S. typhimurium*, *E. coli* (F18ab)	Inactivated bacteria (formaldehyde), immunized separately; adjuvant not specified	Pharmaceutical composition, powder that can be formulated for oral use	Butz et al. (359)
Rotavirus, *Escherichia coli (*ETEC*)*, *E. coli (Shiga toxin-positive)* and *Salmonella*	Inactivated antigens used separately	IgY combined with colostrum as anti-diarrhea supplement	Grabowsky et al., Gaensbauer et al. (362, 363)
*E. coli.* (ETEC) *Salmonella enteritidis*, *Salmonella typhimurium*, *H. pylori*	Mixed (all four, or first three) or separately used, inactivated bacteria (formaldehyde); adjuvant not specified	Ice-cream, yogurt, mayonnaise (whole eggs used)	Lee et al. (366)
*E. coli.* (ETEC), *H. pylori*	Mixed inactivated bacteria (formaldehyde); adjuvant not specified	Ice-cream, yogurt	Baek et al. (365)
*E. coli* (ETEC), *H. pylori*	Mixed inactivated bacterial antigens (formaldehyde); adjuvant not specified	Kimchi, soy sauce, soybean paste, fruit juice	Choi et al., Lee et al., Baek et al. (367–370)
*E. sakazakii*, *Salmonella*	Mixed antigens	Immune milk powder for children	Bao et al. (371)
*A. hydrophila*, *B. cereus*, *C. jejuni*, *C. perfringens*, *E. coli* (0157:R7), *Lactobacillius*, *L. monocytogens*, *S. cerevisae*, *S. enteritidis*, *S. typhimurium*, *S. aureus*, *S. epidermidis*	Separately used thermally or chemically inactivated bacteria cells; adjuvant not specified	Processed meat products, e.g., grill sausages as a sterilization or bacteria growth inhibition substance	Song et al. (372)
Mouth			
*S. mutans* (serotype C, D, and G), *A. actinomycetemcomitans*, *F. nucleatum*, *P. gingivalis*, *A. viscosus*, *C. ochracea*, *T. denticola*, *B. forsythus*	Mixed bacterial antigen	Anti-caries and anti-periodontal disease and anti-halitosis toothpaste	Paau and Yang (373)
*S. mutans* and *S. sobrinus;* *F. nucleatum* and *P. gingivalis*,	Whole cells or whole cells and cell fragments. The immunization with all antigens or with mixed two bacteria at a time (anti-caries or anti-periodontal)	Anti-caries, anti-periodontal disease or anti- bad breath toothpaste	Chen et al. (374, 375)
*P. gingivalis*, *F. nucleatum*, *S. moorei*	Mixed bacterial antigen	Mouthwash for preventing and treatment of bad breath	Zhao et al. (376)
*P. gingivalis*, *F. nucleatum*	Mixed or separately used whole cells with or without bacteria fragments fraction	Anti-bad breath mouthwash	Chen et al. (377)
*S. mutans*, *P. gingivalis*, *H. pylori*	Separately used inactivated whole cells	Oral spay, lozenge, chewing gum	Pang et al. (378)
*P. gingivalis*, *A. actinomycete*	Mixed disintegrated bacterial suspension	Oral spay	Zou and Jia (379)
*S. aureus*, *E. coli*, *S. mutans*, *P. gingivalis*, hemolytic *Streptococcus*, *C. albicans*	Mixed bacterial antigen; adjuvant not specified	Medical chewing gum	Meng et al. (380)
*A. actinomycetemcomitans* (polysaccharide antigen), *P. gingivalis* (polysaccharide antigen), *F. nucleatum*, *C. rectus*, *B. forsythus* *T. denticola*, *S. mutans*	Separately immunized, for polisacharides the BSA conjugates were prepared, for the rest of bacteria, formaldehyde inactivated whole cells were used; adjuvant not specified	Tooth coating composite	Oka (381)
*S. mutans*, *P. gingivalis*, *C. nucleus*, *A. viscosus*, *Actinomyces* spp.	Mixed bacterial antigens	Anti-caries and anti-periodontal disease milk powder	Sheng et al. (382)
Skin			
*P. aeruginosa*, *E. coli*, *S. aureus*, β-hemolytic *Streptococcus*	Mixed proteins antigen obtained after cells disruption	Cream for burn treatment	Chao & Zhenqiang (383)
*P. aeruginosa*, *E. coli*, *S. aureus*	Mixed inactivated bacteria cells	Antibacterial spray for application on burns and wounds	Lin (384)
*S. aureus*, *E. coli*, *A. baumannii*, *P. aeruginosa*, β-hemolytic *Streptococcus* group A, *C. albicans*	Mixed inactivated bacteria	Wound treatment cleaning composition, band-aid, and liquid	Yongxiang et al., Meng et al. (385, 386)
*P. acnes*, *S. aureus*	Mixed bacterial cell fragments soluble fraction from disintegrated cells	Anti-acne spray	Fu (387)
*P. acnes*, *S. aureus*, *P. aeruginosa*, *E. coli*, hemolytic *Streptococcus*, *C. albicans*	Mixed inactivated cells	Mask with antibacterial and acne activity	Nong et al. (388)
*P. acnes*, *S. aureus*, *P. aeruginosa*, hemolytic *Streptococcus*, *C. albicans*	Mixed inactivated cells	Antibacterial and anti-acne toner, mask mud, and gel sleep mask	Meng et al. (389)
Respiratory tract			
*S. aureus*, hemolytic *Streptococcus*, *S. pneumoniae*, *E. coli*, *A. baumannii*, *P. aeruginosa*	Mixed inactivated bacterial cells	Inhalation solution for respiratory tract infections	Nong et al. (390)
*S. aureus*, *E. coli*, β-hemolytic *Streptococcus*	Mixed or separately used inactivated bacterial antigens	Throat-moistening lozenges	Jiang et al. (391)
S. *pneumoniae*, β-hemolytic *Streptococcus* group A, *H. influenzae*, *S. aureus*, *S. graminis*, *S. albicans*, *M. catarrhalis*	Mixed bacterial antigens	Anti-pharyngitis milk powder for infants	Sheng et al. (382)
Gynecological			
Papillomavirus, *S. aureus*, *E. coli*, *P. aeruginosa*, *C. albicans*, β-hemolytic *Streptococcus* group A	Mixed inactivated antigens	Vaginal gel or foaming agents	Fu et al. (392, 393)
*C. albicans*, *S. aureus*, *N. gonorrhoeae*, *G. vaginalis*, (papillomavirus *)	Protein extract used as a mixture.	Preparation for gynecological inflammations	Zhang (394)
*S. aureus*, *P. aeruginosa*, *E. coli*, *S. mutans*, *C. albicans*, β-hemolytic *Streptococcus* group A	Mixed inactivated antigens	Composition against mouth and gynecological infections	Duan et al. (395)

*Optional HPV recombinant proteins.

If not specified in the Vaccine Preparation column, then FCA/FIA was used as an adjuvant.

Although some of the IgY compositions for oral use target bacteria that can also be responsible for respiratory tract infections, few formulations designed specifically to target this type of infections have been patented. Avian antibodies prepared *via* immunization with mixed antigens specific to *S. aureus*, *Streptococcus hemolyticus*, *S. pneumoniae*, *E. coli*, *A. baumannii*, and *P. aeruginosa* were used for the preparation of inhalation solutions to treat respiratory tract infections. Nebulization was performed twice a day (4 ml each time; 1.25%–2% IgY solution) for 5 to 7 days. In the groups of a minimum of 50 patients divided into groups with upper or lower respiratory tract infections or with bacterial pneumonia, approximately 90% of patients reported relief or complete disappearance of symptoms. Unfortunately, it is not clear how the study control was designed (390). The effectiveness of throat-moistening lozenges prepared with IgYs of antigenic specificity toward *S. aureus*, *E. coli*, and β-hemolytic *Streptococcus* was tested by a large cohort of volunteers (1,500, experimental; 1,500, control group). The experimental group received five tablets for 1 day. As a result, 96.65% of the volunteers confirmed the moisturizing effect of the IgY-containing lozenges (391). The hyperimmune egg yolk antibodies with specificity directed to respiratory tract pathogens were also added into the infant milk powder as a possible route of delivery (382).

The products designed for topical skin application utilize another route for providing the IgY-based passive immunity. IgY-containing skin products can be divided into cosmetics and wound (including burns) healing substances with antibacterial properties. The burn treatment cream invented by Lu and Qi contains IgYs generated with protein antigens isolated from *P. aeruginosa*, *E. coli*, *S. aureus*, and β-hemolytic *Streptococcus*, as active ingredients display the bacteriostatic activity (verified *in vitro*) (383). IgYs with a similar specificity profile were also used for the preparation of antibacterial sprays for burn healing (384). Antibodies manufactured with a set of antigens (*S. aureus*, *E. coli*, *A. baumannii*, *P. aeruginosa*, β-hemolytic *Streptococcus* group A, and *C. albicans*) were used for the development of wound-healing liquids, cleansing composition, and band-aid. According to patent description, the developed formulations provide a significant antibacterial effect *in vitro* while wound-healing effectiveness was tested in rabbits. During treatment, no edema or erythema was observed in the injured area, which started to build up after the treatment was removed. On the basis of *in vivo* studies and tests performed on human subjects, the products were evaluated as non-irritative and safe for use on the skin. In addition, clinical tests (group of 30 volunteers) of the products provided confirmation of the improvement of wound-healing time with the superior performance of the liquid and band-aid formulations (385). Other compositions in spray form for burn healing based on IgYs of the same antigen specificity were tested in human patients with a three-times–per–day treatment, and inhibition of bacteria growth was analyzed up to 8 days. The targeted bacteria growth was reduced to values below 10% as compared to the nontreated group on day four of the treatment and below 1% on day 8 (386).

Anti-acne cosmetics represent another group of IgYs containing products intended for skin application. Apart from anti-*Propionibacterium acnes* specificity, antibody compositions usually include anti–*S. aureus* activity. Such two-component mixture of antigens has been selected for the generation of IgYs that were subsequently used for the development of a topical spray. Furthermore, the authors showed that binding of targeted bacteria by IgYs promotes phagocytosis by human neutrophils *in vitro* (387). Different approach was based on a set of antigens including *P. acnes*, *S. aureus*, *P. aeruginosa*, *E. coli*, hemolytic *Streptococcus*, and *C. albicans* used to generate hyperimmune IgYs that were further used for the preparation of an antiacne and antibacterial face mask. The antibacterial activity of six formulations presented were tested *in vitro* confirming their effectiveness not only toward *P. acnes* but also against all targeted microorganisms. The formulations were additionally tested *in vivo* on rabbits (eye and skin irritation tests) to ensure product safety. Among few skin care–related parameters tested in human subjects, the anti-acne effect was evaluated. The mask was used by volunteers once every 2 days, overnight, for 1 month. The test subjects reported anti-acne effect as well as skin improvement. Some differences between formulations were observed what can be a result of the presence of other active ingredients or stability of IgYs in different formulations (388). The same group adopted a similar approach for the development of anti-acne toner, mask mud, and gel sleep mask using hyperimmune IgYs specific toward the same set of microorganisms except *E. coli* (389).

Among inventions that apply IgYs as the active components of products for topical use, the gynecological products including gels or foams have been developed. However, in addition to anti-bacterial IgYs, the products contain IgYs generated with viral and/or fungi antigens. Two products, foaming agent and gel, were prepared with antibodies generated with six infectious agents: papillomavirus, *S. aureus*, *E. coli*, *P. aeruginosa*, *C. albicans*, and β-hemolytic *Streptococcus* group A, and were further evaluated *in vivo* in humans. Antibacterial activity was confirmed after 1 week of daily use of the product (once daily in the morning) (392, 393). Different invention describes a series of compositions designed to reduce gynecological inflammations that contain IgYs specific to *C. albicans*, *S. aureus*, *N. gonorrhoeae*, *G. vaginalis*, and papillomavirus. The products showed bacteriostatic activity *in vitro* and the ability to reduce the inflammation symptoms (e.g., itching, burning, or changes in tissue appearance) *in vivo* in three case study examples (treatment two times a day for at least 28 days) (394). Some of the compositions developed have more universal applications and can be used in both mouth and gynecological infections (395).

## IgY market

Although the IgY industry has been developing for more than two decades, there are not many products intended for humans that are very well established on the market. There is a significant difference in the consumer’s approach to these products in Asia and western countries. Immunoglobulin Y is much better recognized as a health supporting supplement in veterinary use, which is discussed at the end of the paragraph (90). The end-user products presented below are just a few examples of globally available food, hygiene, and cosmetics with the specific IgYs as functional ingredients. Other products, sometimes, with the same active components as that presented, e.g., Ovalgen^®^ or IgY Max^®^, are also available on the market (**Table 15**).

**Table 15:** Commercially available products intended for human use.

Product Name	Company	IgY Specificity	Described Activity	Reference
IgY Max^®^	IgY Nutrition	Antigens from 26 human relevant bacteria (killed bacteria)	Treatment and prevention of microbial imbalance	Hewlings (356)
IgYGate^®^ GastimunHP	EW Nutrition	*H. pylori*	Gut health and support during gastritis/gastric ulcers treatment	Yoshikatsu et al. (148)
IgYGate^®^ GastimunHP Plus	EW Nutrition	*H. pylori* *Lactobacillus johnsonii*	Gut health and support during gastritis/gastric ulcers treatment	Aiba et al. (143)
IgYGate^®^ DC-PG	EW Nutrition	*Porphyromonas gingivalis* (gingipain) *Streptococcus mutans* (cell-associated glucosyltransferase)	Teeth and gums strength and support during and after caries and gingivitis	Nguyen et al.; Yokayama et al. Uasa et al.; Kodama et al. (215, 225, 227, 396, 397)
Ig-Guard Helico^®^	ADbiotech Co., Ltd.	*H. pylori*, formalin inactivated bacteria used for immunization as an antigen-heterologous antibody complex	Support gastrointestinal tract health	Cheong et al. (398, 399)
AdoraCURE line:	ADbiotech Co., Ltd.	Acne bacteria: *Propionibacterium acnes*, *Propionibacterium pneumoniae*, *Staphylococcus aureus*, *Staphylococcus epidermidis*, *Micrococcus luteus* and *Actinomyces israelii* inactivated with formalin	Improvement of acne prone skin	Hong Gul (400)
Ulcer-Lock Helico-IgY	DAN Biotech Inc.	*H. pylori* *Escherichia coli* (O157:H7) *Salmonella* spp.	Preventing gastritis, gastric ulcers, food poisoning, and diarrhea	Ahn et al. (401)
Gastro-Lock	DAN Biotech Inc.	Human Rotavirus *Escherichia coli* *Salmonella* spp.	Preventing diarrhea, improvement of intestine microbiome	
AtoIB	DAN Biotech Inc.	*Staphylococcus aureus* recombinant endotoxin B	Support treatment of atopic dermatitis	Ark et al. (402)
Adsorb line	Zeal Cosmetics	*Propionibacterium acnes*, *Staphylococcus aureus*, *Pseudomonas aeruginosa*	Healthy, conditioned skin	Tsukamoto et al. (403, 404)
Orecare U Smile	TIENS	*Streptococcus mutans*	Children’s oral health	Wu (405)

IgY Max^®^ is the most complex IgY-containing supplement on the market. It contains hyperimmune egg yolk IgY antibodies produced by a vaccine based on antigens of 26 relevant human bacteria, including *Eschericha coli*, *Klebsiella pneumoniae*, *Pseudomonas aeruginosa*, *Salmonella* spp., *Streptococcus* spp., *Pseudomonas vulgaris*, *Propionibacterium acnes*, and *Haemophilis influenzae* (356). The ingredient activity is directed against non-beneficial species of human bacteria with its primary function being the treatment or prevention of microbial imbalance and support of the immune system in the intestine. The invention describes the human subject study to evaluate the safety, tolerability, and efficacy of the product. The results including an analysis of the serum level of markers of gut permeability such as zonulin, diamine oxidase, and histamine (connected with disorders such as Celiac’s, Chrohn’s disease, and colitis) showed that hyperimmune IgY treatment improves gut integrity by reducing inflammation and influencing the microbiome. In the second experiment, human subjects supplemented the daily diet with IgY Max^®^ and probiotics. The study showed that the combination of hyperimmune IgY with probiotics but also, to a lesser extent, the monotherapy of each supplement allowed the decrease in inflammation, which was determined by assessing the CRP parameter in sera. Among the selected antigens are microorganisms capable of inhabiting not only the digestive tract but also the skin (or wounds), respiratory system, or other mucosa (e.g., *Propionibacterium acnes* or *Pseudomonas aeruginosa*). The inventor listed other, e.g., topical roots of application for IgY Max^®^, therefore, the applicability of the product might become wider. The product was also a subject of a completed clinical trial with the group of 100 participants and different doses of IgY received daily for 12 weeks. The primary goal was to verify the level of C-reactive protein, but no follow-up results are posted as of now (Igy Nutrition, LLC, *The Influence of IgY Max on Inflammatory Markers and the Gut Microbiome*, 2016-2018, NCT02972463. Updated: 5 September 2018. Accessed 21 February 2023). In the patent description of IgY Max^®^, Freund’s complete adjuvant was listed as one to be used for immunization. Although Freund’s complete adjuvant is not recommended for use, even in animals, it is highly likely to also increase the pool of specificity of hyperimmune egg yolk antibodies by adding anti-mycobacteria IgY. It is not likely that the anti-mycobacteria IgY will have a comparative, heterologous protective effect similar to direct vaccination, due to the different underlying mechanisms that are responsible for the heterologous response toward unrelated infections observed after mycobacteria vaccination (406). Nevertheless, infections caused by members of the *Mycobacterium tuberculosis* complex are prevalent among wildlife and domestic animals, including humans (407); therefore, the benefits of including anti-mycobacteria specificity should not be overlooked.

Among the IgY antibody-containing supplements introduced to the market, there are several specifically designed to target *H. pylori* infections. ADbiotech Co., Ltd., the manufacturer of Ig-Guard Helico^®^, patented the method for the production of anti–*H. pylori* hen yolk antibodies based on formalin-inactivated whole-cell bacteria. Most importantly, the vaccine in addition to the antigen and adjuvant includes a small fraction of the complex between the antigen and the heterologous antibody (mammalian). The introduction of such complex to the vaccine in the right proportion to the main antigen allows for the enhancement of the immune response and, consequently, the quantity of specific IgY in yolk (398). The application of immunological complexes to enhance the host’s immune response is similar to that demonstrated for murine anti-MUC1 monoclonal antibodies used in cancer treatment (408). The company also holds the rights to the invention of the multicomponent vaccine used for IgY production. The mixture of formalin-deactivated *H. pylori* contains recombinant proteins urease A, urease B, CagA, and VacA. The resultant hyperimmune egg yolk antibody had superior *H. pylori* growth inhibitory potency compared to the activity of the specific IgY antibody generated with only *H. pylori* inactivated cells or *H. pylori* cells + urease A (399). *In vivo* tests in mice with Ig-Guard Helico^®^ treatment were discussed in the ***H. pylori*** section (123). The mentioned products are not the only ones available on the market to treat *H. pylori* infections. The EW Nutrition IgYGate^®^ line provides an end-user diet supplement that includes Ovalgen^®^, the functional ingredient that contains IgY antibody. The products of IgYGate^®^ brand include GastimunHP with IgY specific toward *H. pylori urease* and GastimunHP Plus, which additionally contain the probiotic *Lactobacillus johnsonii.* In the controlled clinical trial study [Immunology Research Institute in Gifu, 2016-2021, *Effect of Chicken Egg Antibody (IgY) on Patients With Chronic Gastritis*, NCT02721355. Updated 28 January 2021. Accessed 10 March 2023] on patients with chronic gastritis receiving standard treatment alone or in combination with the anti-urease IgY antibody (GastimunHP), ^13^C-UBT performed before the treatment and at week 8 revealed the decrease from 161.64 (baseline) to 49.42 (week 8) for the group receiving IgY along with standard therapy, and from 158.54 to 73.37 in the group with standard therapy only. The clinical trial on the treatment of patients with *H. pylori* infection and peptic ulcer disease with GastimunHP Plus (combining anti-urease IgY and *L. johnsonii*) was terminated due to COVID-19 [Institute of Gastroenterology and Hepatology, Vietnam, *Effectiveness of GastimunHp Plus in Supporting the Treatment of Peptic Ulcer Disease With Helicobacter Pylori Infection (GasHp)*, 2019-2021, NCT04025983. Updated 19 October 2021.Accessed 10 March 2023].

Another product of EW Nutrition, IgYGate^®^ DC-PG, is a combination of Ovalgen^®^ DC, a dental health support product with anti–*S. mutans* IgY as an active component and Ovalgen^®^ PG with immunoglobulin Y specificity to *P. gingivalis.* Both products were tested in human subjects, and the results on specific IgY activity are described above in the sections on *S. mutans* and *P. gingivalis*, respectively (215, 225, 227, 396, 397). The specific anti–*S. mutans* antibodies are also used as an ingredient of toothpaste, for example, Orecare U Smile intended for children’s oral health care. The effectiveness of toothpaste was tested in children (ages 4–12), who used it to brush their teeth for 2 months (twice a day), with a significant reduction of dental caries and oral ulcers (405).

The compositions offered by DAN Biotech provide IgYs specificity toward multiple bacterial antigens. In the case of Ulcer-Lock and Helico-IgY antibodies, the targets are *H. pylori*, *E. coli*, and *Salmonella* spp. (*S. typhimurium* and *S. enteritidis*), whereas, in the case of Gastro-Lock IgY, the specificity includes *E. coli*, *Salmonella* spp., and human rotavirus. On the basis of patents held by the company, anti–*H. pylori* IgY antibodies are manufactured with recombinant OMP used as an antigen (401). According to the information presented on the producer website, a test of Ulcer-Lock efficacy in Mongolian gerbil (*H. pylori* infection) showed a reduction in lymphocyte and neutrophil infiltration in the group receiving treatment with 10 mg of IgY compared to the untreated control group and a 1-mg IgY group. The patent’s information relates to company’s products description and includes antigens: recombinant adherent protein—intimin (enterohemorrhagic *E. coli* O157: H7 strain and enteropathogenic *Escherichia coli*) (258, 409), recombinant fimbria adhesin (cfaB gene of enterotoxigenic *E. coli*) (410), and flagella protein (isolated from *Salmonella enteritidis*, *Salmonella typhimurium*) (411).

The ACfine line includes cosmetic products designed mainly for acne-prone skin. Among the active ingredients, IgY antibodies are the ones playing an antibacterial role. According to the patent application, the manufacturer holds the priority rights to cosmetic products including the IgY antibodies specific toward *Propionibacterium acnes*, *Propionibacterium pneumoniae*, *Staphylococcus aureus*, *Staphylococcus epidermidis*, *Micrococcus luteus*, and *Actinomyces israelii*, bacteria responsible not only for acne in young people but also for other skin infections. The activity of hyperimmune IgY was verified in the model of TPA-induced ear edema in mice. The level of pro-inflammatory IL6 was reduced in the group treated with IgY and was concentration dependent. The analysis of MPO activity indicates a reduction in epidermal hyperplasia and the infiltration of inflammatory cells (400).

Another product line in the field of cosmetic industry is an Adsorb series by Zeal Cosmetics utilizing ostrich antibodies with anti–*P. acnes*, anti–*S. aureus*, and anti–*P. aeruginosa* activity along with few other antigenic specificities (non-bacteria related). Ostrich IgYs specific for *S. aureus* or *P. acne* were obtained after immunization with homogenized bacteria. The specific antibodies were able to suppress bacteria growth and react not only with the antigen used for immunization but also with selected, pure toxins, and enzymes. Anti–*S. aureus* and anti–*P. acnes* antibodies were also tested in human subjects with atopic dermatitis or acne as monotherapy and in combination, showing improvement after the first week of treatment in 73% of patients with atopy (anti–*S. aureus* IgY) and 69% of patients with acne (anti–*P. acnes* IgY). The combined therapy resulted in symptoms alleviation in 81% and 59% of patients, respectively. In addition, treatment of pyoderma in dogs with the use of anti–*S. aureus* antibody produced in ostriches substantially reduced skin lesions and improved the histopathological and inflammatory signs of the disease in three of the four tested dogs. However, this skin condition can be caused not only by *Staphylococcus* but also by *Streptococcus* infection, and, therefore, the lack of a therapeutic response can be attributed to another source of infection (403, 404).

The AtoIB series is designed to help with the treatment of atopic dermatitis caused predominantly by *Staphylococcus aureus.* Skin colonization by these bacteria is associated with the disruption of the protective barrier and microbial diversity that allows easier viral infections. The pathogenesis of *S. aureus* includes immune responses (e.g., elevated IgE) modulated by the bacterial virulence factors, including enterotoxin B (412). Cosmetic products developed by DAN Biotech use anti–enterotoxin B antibody to help minimize symptoms and support treatment. Although the website product description only mentions enterotoxin B as an antigen, the company is the patent holder for the production of specific IgY antibodies induced by immunization with recombinant enterotoxin combined with lysates from *Staphylococcus aureus* and *Streptococcus pyogenes.* Topical *in vivo* treatment in mice with wild-type toxin and IgY showed a reduction in the IgE level to the level of negative control. The therapeutic effect of the patented cosmetic compositions was confirmed in the test carried out in human subjects for 6 weeks with the application of the product twice daily, with a significant alleviation of symptoms of atopic dermatitis, including the reduction in colonization of *S. aureus* (402). Two compositions were tested, one with *S. aureus* enterotoxin B–specific antibodies and the other combining them with *S. aureus* lysate generated IgY. The results showed the reduction in itching (49.9% and 42.7%), reduction in eczema area and severity index (55.3% and 45.8%), and *S. aureus* colonization reduction (33.7% and 15.7%) with the superiority of composition including both antibodies. The results for negative control were 27.5%, 33.5%, and 13.2%, respectively.

In veterinary applications, egg yolk antibodies are a good solution as a supplement to health and immune system support in animals, as IgYs are well tolerated even by very young cubs. Globigen^®^ is a line of products developed by EW Nutrition and designed for piglets and calves. It provides support of the animal immune system and secures intestinal tract health, acting against diarrhea. The EW group member (Ghen Corporation, Japan) is a patent holder company where manufacturing of anti–*Escherichia coli*, anti–*Salmonella dublin*, and anti–*Clostridium perfringens* IgYs (among others) along with *in vivo* animal study is presented (413). For one of the commercially available additives of Globigen^®^, the anti-diarrhea protective potential was tested *in vivo* in 1-month-old piglets. The animals received IgY (in combination with phytomolecule-based supplement) in the diet 1 week before inoculation with the *E. coli* K88 strain, after three day-by-day inoculations, the experiment was continued for 42 days with daily IgY supplementation. The study also included the group receiving antibiotic treatment. The diarrhea incidents observed in the group receiving IgY during challenge and the first week after inoculation were significantly lower than in the positive control group with a reduction of 45.23% (positive control) to 30.55% (IgY group) during challenge, and 60.88% to 38.09%, days 1–7 after challenge. The difference, however, was not as substantial as in the case of antibiotic treatment. However, the treatment of piglets with IgY antibodies not only allowed a decrease in the incidence of diarrhea, but also had a positive impact on intestinal morphology and immunity (414). The results obtained with Globigen^®^ just confirmed the previous observations on the protective activity of anti–*E.coli* IgY against diarrhea and subsequent mortality in piglets (415, 416).

Another line of veterinary products is offered by ADbiotech and includes feed supplements mainly for calves and swine but also for dogs and cats (young), poultry, shrimp, and salmon. In addition to the patent describing the manufacturing of IgYs intended for human use (398–400), the company has a substantial portfolio with inventions of veterinary importance. Shrimp production can be endangered by early mortality syndrome (EMS), which is caused by bacteria from the genus *Vibrio* and the white spot syndrome virus. In the patented solution, IgY antibodies designed to provide protection against EMS were generated after hen immunization with mixed antigens including: *Vibrio parahaemolyticus*, *Vibrio harveyi*, and *Vibrio anguillarum*; recombinant OMP of *V. parahaemolyticus* and *V. harveyi*; and recombinant protein of white spot virus (VP28). The immunized groups were divided to verify the effectiveness of IgY and included the group receiving a mixture of all immunogens, the groups without *Vibrio* recombinant proteins or without all recombinant proteins. The activity of the IgYs was confirmed by immunochemical methods and verified in a bacteria growth inhibition test. Hyperimmune egg yolk antibody produced after immunization with all prepared antigens inhibited bacterial growth most effectively (417). In an alternative, patented approach, an additional bacteria strain (*Vibrio parahaemolyticus E1*) and an inactivated white spot virus were used together with three bacteria listed above, but the immunogen was enriched with the antigen-heteroantibody complex by addition of a specific mouse IgG antibody. The hyperimmune IgYs were able to increase the survival rate of shrimps from 40% (normal diet) to 75% (0.5% IgY in diet), 7 days after infection with *V. Parahaemolyticus* (418). A similar approach with the antigen-heteroantibody complex used as an immunogen was applied in the case of the invention related to the production of egg yolk antibodies specific to intracellular bacteria *Piscirickettsia salmoni* (419), *the* causative agent of piscirickettsiosis, a highly severe and prevalent disease in salmon (420). The immunogen composed of inactivated cells from *P. salmonis* and the complex of these cells and the mouse IgG against them. The IgY inhibited the growth of the target bacteria in 99.99% when tested in a series of dilutions up to 1:4,000 (419). This approach was also used to manufacture specific IgY by immunization of hens with mixed antigen, including *E. coli* and the rota virus as agents that cause digestive disease in calve (421) or *E. coli* and porcine epidemic diarrhea virus (PEDV) in the case of IgY intended for swines (422). A more classic approach for designing immunogens was presented in other inventions where IgYs intended as food additives for piglets were produced with the mixed immunogen comprising inactivated *Salmonella tiphimurium*, *Salmonella choleraesuis*, transmissible gastroenteritis virus, PEDV and *Escherichia coli* (423), or duck hepatitis virus, *S. thyphimurium*, *Rimeriella anatipestifer*, and *E. coli* in the case of IgY intended for ducks (424).

Dan Biotech also provides veterinary solutions that include the utilization of IgY antibodies specific toward bacterial antigens mainly for calves but also for swine (Ig Lock line). The products provide protection against the strains of *E. coli* and *Sallmonella* spp. with the addition of viral pathogens specific for the species.

Several other veterinary products are present on the market, mainly not only feed supplements/additives but also, for example, oral hygiene product (X ˘mile^®^ Gel) with anti–*P. gingivalis* yolk antibody as a functional ingredient. Many of these products are in the form of ready-to-use formulations intended for young animals.

## Discussion

**Figure 1:**
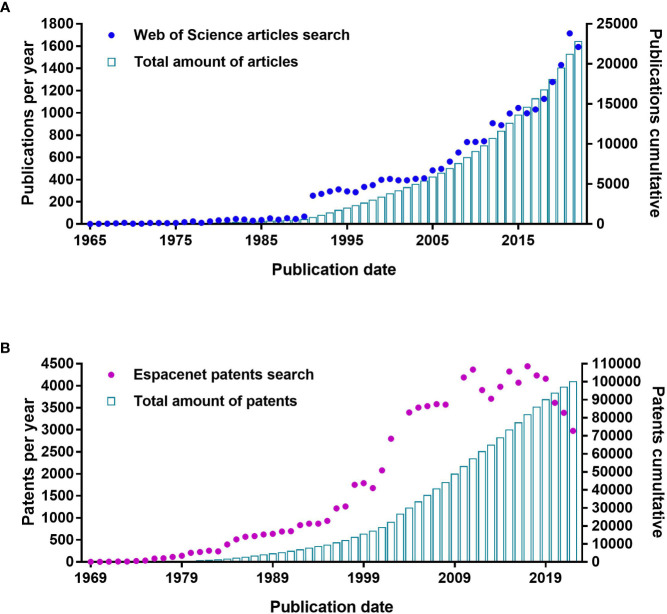
Number of articles **(A)** and patents **(B)** concerning IgY antibodies and bacterial infections according to date. The keywords used to search for articles (Web of Science) and patents (European Patent Office) databases are “bacteria AND IgY OR immunoglobulin Y OR yolk immunoglobulin”. The date range is limited to the end of 2022.

The production of avian antibodies specific to bacterial antigens continues to attract the attention of researchers and the biotech industry. Apart from the diagnostic segment of the IgY market, there is significant potential for antibacterial IgYs to be utilized as preventive or therapeutic compounds. To assess how the interest in IgY antibodies is changing, we performed a simple analysis of the development of new publications and patents (**Figure 1**). The first mention about IgY antibodies associated with bacteria (keywords: “bacteria AND IgY OR immunoglobulin Y OR yolk immunoglobulin”) according to the Web of Science database was noted in 1965. The number of new publications began to increase significantly in 1991 (over 200 per year). In the last year analyzed (2022), the number of new publications reached 1,600, giving a total of over 22,000 deposited papers (**Figure 1A**). In the case of patents, the first recorded application was in 1969. Patent growth accelerated significantly in the early 2000s (**Figure 1B**), and, by 2022, the total number of patents was around 100,000.

The passive immunization through IgY antibodies is easily achievable for many bacterial infections, as the colonization area is accessible without crossing integuments of the body. In our work, we have collected information on pathogens of the digestive tract (such a as *H. pylori*, *E. coli*, *S. typhimurium*, *C. jejuni*, and *C. difficile*), oral cavity (*S. mutans*, *F. nucleatum*, and *S. moorei*), and wounds (*S. aureus*) that are sensitive to the antibacterial effect of specific IgY antibodies. We indicate the possible ways and conditions of administration of antibodies to bring about the expected effect. Our analysis shows that topical or oral applications are mainly used. The oral application has its limitations connected mainly with IgY stability in conditions encountered in the digestive tract. On the other hand, IgY is natural egg protein with minimal risk of side effects. It is unexpected that the human-dedicated product market is not growing rapidly, especially given the accelerated growth in the natural/green food supplements market. One of the reasons is that some products are offered by locally operating companies and are not visible on the global market. The good example is ROMVAC Company offering several products based on IgY (IMUNOINSTANT brand). The company has several national patents describing the production, processing, and application of IgYs [for example (425, 426)], and the antibody activity was presented in scientific literature (357). Considering the costs of infrastructure required for IgY antibody production, low to moderate prices of raw antibodies or ready to use products, and generally low market interest, the slow growth of the IgY product range is understandable.

The veterinary segment of the IgY antibody market is increasingly more recognizable and growing, which can be attributed to the popularity and thus market pressure toward animal production with the minimal use of antibiotics. The activity of IgY antibodies in many cases is not as potent as those of antibiotics, for treatment of infections, but can be used freely as preventive agents as they are natural, nontoxic, well tolerated even by young animals, and easy and inexpensive to produce. In contrast to antibiotics, oral IgY supplements not only do not deplete the intestinal tract microbiome but also support it just as they support the immune system. Last but not least, IgY supplements do not need to be highly purified as they can be an additional source of protein, and the presence of lipids improves the stability of antibodies in the digestive tract. In developing countries, animal welfare is managed in a different way than in high-income countries because of the differences in consumer awareness, law and farming policies, and purchasing options. Parlasca et al. highlight the issue of limited options of nutritious food and supplementation. On the other hand, traditional farmers tend to develop closer bonds with their animals, and, in some developing countries, citizens become more sensitive to the animal welfare (427). The possibility to use IgY-derived food additives, ointments, or other preparations that could serve as prophylaxis or therapy for animals may be easily implemented in such regions because of low costs of the production and no risks connected with the use of such preparations and, thus, little need for veterinary supervision. Furthermore, because of the stability of IgYs, there are no storage or transportation issues associated with these preparations, as is sometimes, for example, the case with vaccines.

## Author contributions

AŁ-S, RG, and MS designed the concept of the work, wrote the manuscript and reviewed the final version. All authors contributed to the article and approved the submitted version.

## References

[1] Jechalke S Heuer H Siemens J Amelung W Smalla K. Fate and effects of veterinary antibiotics in soil. Trends Microbiol (2014) 22:536–45. doi: 10.1016/j.tim.2014.05.005 24950802

[2] Cassini A Högberg LD Plachouras D Quattrocchi A Hoxha A Simonsen GS. Attributable deaths and disability-adjusted life-years caused by infections with antibiotic-resistant bacteria in the EU and the European economic area in 2015: a population-level modelling analysis. Lancet Infect Dis (2019) 19:56–66. doi: 10.1016/S1473-3099(18)30605-4 30409683 PMC6300481

[3] World Health Organization. Global antimicrobial resistance and use surveillance system (GLASS) report 2021 (2021). Available at: http://www.who.int/glass/resources/publications/early-implementation-report-2020/en/.

[4] Goossens H Ferech M Vander Stichele R Elseviers M. Outpatient antibiotic use in Europe and association with resistance: a cross-national database study. Lancet (2005) 365:579–87. doi: 10.1007/978-3-211-89836-9_1109 15708101

[5] Llor C Bjerrum L. Antimicrobial resistance: risk associated with antibiotic overuse and initiatives to reduce the problem. Ther Adv Drug Saf (2014) 5:229–41. doi: 10.1177/2042098614554919 PMC4232501 25436105

[6] Maron DF Smith TJ Nachman KE. Restrictions on antimicrobial use in food animal production: an international regulatory and economic survey. Global Health (2013) 9. doi: 10.1186/1744-8603-9-48 PMC3853314 24131666

[7] Li X Wang L Zhen Y Li S Xu Y. Chicken egg yolk antibodies (IgY) as non-antibiotic production enhancers for use in swine production: a review. J Anim Sci Biotechnol (2015) 6:1–10. doi: 10.1186/s40104-015-0038-8 26309735 PMC4549021

[8] Mathew AG Cissell R Liamthong S. Antibiotic resistance in bacteria associated with food animals: a united states perspective of livestock production. Foodborne Pathog Dis (2007) 4:115–33. doi: 10.1089/fpd.2006.0066 17600481

[9] Manyi-Loh C Mamphweli S Meyer E Okoh A. Antibiotic use in agriculture and its consequential resistance in environmental sources: potential public health implications. Molecules (2018) 23(4):795–843. doi: 10.3390/molecules23040795 29601469 PMC6017557

[10] Browne K Chakraborty S Chen R Willcox MD Black DS Walsh WR. A new era of antibiotics: the clinical potential of antimicrobial peptides. Int J Mol Sci (2020) 21:7047. doi: 10.3390/ijms21197047 32987946 PMC7582481

[11] Laterre PF Colin G Dequin PF Dugernier T Boulain T Azeredo da Silveira S. CAL02, a novel antitoxin liposomal agent, in severe pneumococcal pneumonia: a first-in-human, double-blind, placebo-controlled, randomised trial. Lancet Infect Dis (2019) 19:620–30. doi: 10.1016/S1473-3099(18)30805-3 31056427

[12] Hickl J Argyropoulou A Sakavitsi ME Halabalaki M Al-Ahmad A Hellwig E. Mediterranean Herb extracts inhibit microbial growth of representative oral microorganisms and biofilm formation of streptococcus mutans. PloS One (2018) 13:1–24. doi: 10.1371/journal.pone.0207574 PMC6291084 30540782

[13] Leitgeb M Kupnik K Knez Ž. Primožič M. Enzymatic and antimicrobial activity of biologically active samples from aloe arborescens and aloe barbadensis. Biol (Basel) (2021) 10:1–19. doi: 10.3390/biology10080765 PMC8389549 34439997

[14] Klemperer F. Ueber natürliche immunität und ihre verwerthung für die immunisirungstherapie. Arch Für Exp Pathol Und Pharmakologie (1893) 31:356–82. doi: 10.1007/BF01832882

[15] Warr GW Magor KE Higgins DA. IgY: clues to the origins of modern antibodies. Immunol Today (1995) 16:392–8. doi: 10.1016/0167-5699(95)80008-5 7546196

[16] Hamal KR Burgess SC Pevzner IY Erf GF. Maternal antibody transfer from dams to their egg yolks, egg whites, and chicks in meat lines of chickens. Poult Sci (2006) 85:1364–72. doi: 10.1093/ps/85.8.1364 16903465

[17] Spillner E Braren I Greunke K Seismann H Blank S du Plessis D. Avian IgY antibodies and their recombinant equivalents in research, diagnostics and therapy. Biologicals (2012) 40:313–22. doi: 10.1016/j.biologicals.2012.05.003 PMC7106491 22748514

[18] Lee L Samardzic K Wallach M Frumkin LR Mochly-Rosen D. Immunoglobulin y for potential diagnostic and therapeutic applications in infectious diseases. Front Immunol (2021) 12:696003. doi: 10.3389/fimmu.2021.696003 34177963 PMC8220206

[19] Pereira EPV van Tilburg MF Florean EOPT Guedes MIF. Egg yolk antibodies (IgY) and their applications in human and veterinary health: a review. Int Immunopharmacol (2019) 73:293–303. doi: 10.1016/j.intimp.2019.05.015 31128529 PMC7106195

[20] Kyong AL Sung KC Yoon JL Jong HL Nan SK. Acid stability of anti-helicobacter pyroli IgY in aqueous polyol solution. J Biochem Mol Biol (2002) 35:488–93. doi: 10.5483/bmbrep.2002.35.5.488 12359091

[21] Shimizu M Miwa Y Hashimoto K Goto A. Encapsulation of chicken egg yolk immunoglobulin G (IgY) by liposomes. Biosci Biotechnol Biochem (1993) 57:1445–9. doi: 10.1271/bbb.57.1445 7764217

[22] Rahman S Van Nguyen S Icatlo FC Umeda K Kodama Y. Oral passive IgY-based immunotherapeutics: a novel solution for prevention and treatment of alimentary tract diseases. Hum Vaccines Immunother (2013) 9:1039–48. doi: 10.4161/hv.23383 PMC3899138 23319156

[23] Kovacs-Nolan J Mine Y. 17 - using egg IgY antibodies for health, diagnostic and other industrial applications. In: Van Immerseel F Nys Y Bain M, editors. Woodhead publ. ser. food sci. technol. nutr. (Sawston, Cambridge, UK: Woodhead Publishing) (2011). p. 346–73. doi: 10.1533/9780857093929.3.346

[24] Tesar DB Cheung EJ Bjorkman PJ. The chicken yolk sac IgY receptor, a mammalian mannose receptor family member, transcytoses IgY across polarized epithelial cells. Mol Biol Cell (2008) 19:1587–93. doi: 10.1091/mbc.e07-09-0972 PMC2291411 18256279

[25] Carlander D Kollberg H Wejåker PE Larsson A. Peroral immunotheraphy with yolk antibodies for the prevention and treatment of enteric infections. Immunol Res (2000) 21:1–6. doi: 10.1385/IR:21:1:1 10803878 PMC7090601

[26] Lee J Kang HE Woo HJ. Stability of orally administered immunoglobulin in the gastrointestinal tract. J Immunol Methods (2012) 384:143–7. doi: 10.1016/j.jim.2012.06.001 22691618

[27] Wang H Zeng X Lin J. Ex vivo evaluation of egg yolk IgY degradation in chicken gastrointestinal tract. Front Immunol (2021) 12:746831. doi: 10.3389/fimmu.2021.746831 34621278 PMC8490740

[28] Kovacs-Nolan J Mine Y. Microencapsulation for the gastric passage and controlled intestinal release of immunoglobulin y. J Immunol Methods (2005) 296:199–209. doi: 10.1016/j.jim.2004.11.017 15680164

[29] Li XY Jin LJ Lu YN Zhen YH Li SY Wang LH. Chitosan-alginate microcapsules for oral delivery of egg yolk immunoglobulin (IgY): effects of chitosan concentration. Appl Biochem Biotechnol (2009) 159:778–87. doi: 10.1007/s12010-009-8628-6 19412580

[30] Wu X Zhao S Zhang J Wu P Peng C. Encapsulation of EV71-specific IgY antibodies by multilayer polypeptide microcapsules and its sustained release for inhibiting enterovirus 71 replication. RSC Adv (2014) 4:14603–12. doi: 10.1039/c3ra46943c

[31] Gu L McClements DJ Li J Su Y Yang Y Li J. Formulation of alginate/carrageenan microgels to encapsulate, protect and release immunoglobulins: egg yolk IgY. Food Hydrocoll (2021) 112:106349. doi: 10.1016/j.foodhyd.2020.106349

[32] Karachaliou C-E Vassilakopoulou V Livaniou E. IgY technology: methods for developing and evaluating avian immunoglobulins for the *in vitro* detection of biomolecules. World J Methodol (2021) 11:243–62. doi: 10.5662/wjm.v11.i5.243 PMC8472547 34631482

[33] Pauly D Chacana PA Calzado EG Brembs B Schade R. IgY technology: extraction of chicken antibodies from egg yolk by polyethylene glycol (PEG) precipitation. J Vis Exp (2011) i:2–7. doi: 10.3791/3084 PMC3197133 21559009

[34] Schade R Staak C Hendriksen C Erhard M Hugl H Koch G. The production of avian (Egg yolk) antibodies: IgY. Altern to Lab Anim (1996) 24:925–34. doi: 10.1177/026119299602400607

[35] Wan Y Du Q Wang D Ma R Qi R Yang R. Effects of different-sized cages on the production performance, serum parameters, and caecal microbiota composition of laying hens. Animals (2023) 13:266. doi: 10.3390/ani13020266 36670806 PMC9854594

[36] Li JY Liu W Ma RY Li Y Liu Y Qi RR. Effects of cage size on growth performance, blood biochemistry, and antibody response in layer breeder males during rearing stage. Poult Sci (2019) 98:3571–7. doi: 10.3382/ps/pez102 30895313

[37] Marcq C Marlier D Beckers Y. Improving adjuvant systems for polyclonal egg yolk antibody (IgY) production in laying hens in terms of productivity and animal welfare. Vet Immunol Immunopathol (2015) 165:54–63. doi: 10.1016/j.vetimm.2015.02.012 25813905

[38] Hartcher KM Jones B. The welfare of layer hens in cage and cage-free housing systems. Worlds Poult Sci J (2017) 73:767–82. doi: 10.1017/S0043933917000812

[39] Schade R Calzado EG Sarmiento R Chacana PA Porankiewicz-Asplund J Terzolo HR. Chicken egg yolk antibodies (IgY-technology): a review of progress in production and use in research and human and veterinary medicine. ATLA (2005) 33:129–54. doi: 10.1177/026119290503300208 16180988

[40] Sudjarwo SA Eraiko K Sudjarwo GW. Koerniasari, the potency of chicken egg yolk immunoglobulin (IgY) specific as immunotherapy to mycobacterium tuberculosis infection. J Adv Pharm Technol Res (2017) 8:91–6. doi: 10.4103/japtr.JAPTR_167_16 PMC5527699 28795022

[41] Sudjarwo S Eraiko K Sudjarwo G. Koerniasari, the activity of immunoglobulin y anti-mycobacterium tuberculosis on proliferation and cytokine expression of rat peripheral blood mononuclear cells. Pharmacognosy Res (2017) 9:5. doi: 10.4103/pr.pr_66_17 PMC5757326 29333035

[42] Shi H Zhu J Zou B Shi L Du L Long Y. Effects of specific egg yolk immunoglobulin on pan-drug-resistant acinetobacter baumannii. Biomed Pharmacother (2017) 95:1734–42. doi: 10.1016/j.biopha.2017.09.112 PMC7126593 28962078

[43] Jahangiri A Owlia P Rasooli I Salimian J Derakhshanifar E Naghipour Erami A. Specific egg yolk antibodies (IgY) confer protection against acinetobacter baumannii in a murine pneumonia model. J Appl Microbiol (2019) 126:624–32. doi: 10.1111/jam.14135 30353977

[44] Li C He J Ren H Zhang X Du E Li X. Preparation of a chicken scFv to analyze gentamicin residue in animal derived food products. Anal Chem (2016) 88:4092–8. doi: 10.1021/acs.analchem.6b00426 26980703

[45] Mesbahi Moghaddam M Rasooli I Ghaini MH Jahangiri A Ramezanalizadeh F Ghasemkhah Tootkleh R. Immunoprotective characterization of egg yolk immunoglobulin raised to loop 3 of outer membrane protein 34 (Omp34) in a murine model against acinetobacter baumannii. Mol Immunol (2022) 149:87–93. doi: 10.1016/j.molimm.2022.06.010 35785672

[46] Ranjbar A Rasooli I Jahangiri A Ramezanalizadeh F. Specific egg yolk antibody raised to biofilm associated protein (Bap) is protective against murine pneumonia caused by acinetobacter baumannii. Sci Rep (2022) 12:1–9. doi: 10.1038/s41598-022-16894-w 35869264 PMC9307575

[47] Zhen Y Long Y Hou Y Sun L Liao H Feng X. Preparation method, application and medicine composition and preparation of specific egg yolk immunoglobulin (IgY) and acinetobacter baumannii, as well as preparation and kit, CN102977208A. (2012).

[48] Nilsson E Amini A Wretlind B Larsson A. Pseudomonas aeruginosa infections are prevented in cystic fibrosis patients by avian antibodies binding pseudomonas aeruginosa flagellin. J Chromatogr B Anal Technol Biomed Life Sci (2007) 856:75–80. doi: 10.1016/j.jchromb.2007.05.029 17581799

[49] Kollberg H Carlander D Olesen H Wejåker PE Johannesson M Larsson A. Oral administration of specific yolk antibodies (IgY) may prevent pseudomonas aeruginosa infections in patients with cystic fibrosis: a phase I feasibility study. Pediatr Pulmonol (2003) 35:433–40. doi: 10.1002/ppul.10290 12746939

[50] Thomsen K Christophersen L Jensen P.Ø. Bjarnsholt T Moser C Høiby N. Anti-pseudomonas aeruginosa IgY antibodies promote bacterial opsonization and augment the phagocytic activity of polymorphonuclear neutrophils. Hum Vaccines Immunother (2016) 12:1690–9. doi: 10.1080/21645515.2016.1145848 PMC4964812 26901841

[51] Sanches RF dos Santos Ferraro ACN Marroni FEC Venancio EJ. Synergistic activity between beta-lactams and IgY antibodies against pseudomonas aeruginosa *in vitro* . Mol Immunol (2022) 148:1–5. doi: 10.1016/j.molimm.2022.05.010 35640520

[52] Thomsen K Christophersen L Bjarnsholt T Jensen P Moser C Høiby N. Anti-pseudomonas aeruginosa IgY antibodies augment bacterial clearance in a murine pneumonia model. J Cyst Fibros (2016) 15:171–8. doi: 10.1016/j.jcf.2015.08.002 26303991

[53] Thomsen K Christophersen L Lerche CJ Holmgaard DB Calum H Høiby N. Azithromycin potentiates avian IgY effect against pseudomonas aeruginosa in a murine pulmonary infection model. Int J Antimicrob Agents (2021) 57. doi: 10.1016/j.ijantimicag.2020.106213 33256950

[54] Schwartz FA Christophersen L Thomsen K Baekdal S Pals Bendixen M Jørgensen M. Chicken IgY reduces the risk of pseudomonas aeruginosa urinary tract infections in a murine model. Front Microbiol (2022) 13:988386. doi: 10.3389/fmicb.2022.988386 36160201 PMC9505517

[55] Otterbeck A Hanslin K Lantz EL Larsson A Stålberg J Lipcsey M. Inhalation of specific anti-pseudomonas aeruginosa IgY antibodies transiently decreases p. aeruginosa colonization of the airway in mechanically ventilated piglets. Intensive Care Med Exp (2019) 7:21. doi: 10.1186/s40635-019-0246-1 30963317 PMC6453987

[56] Otterbeck A Skorup P Hanslin K Larsson A Stålberg J Hjelmqvist H. Bronchially instilled IgY-antibodies did not decrease pulmonary p. aeruginosa concentration in experimental porcine pneumonia. Acta Anaesthesiol Scand (2021) 65:656–63. doi: 10.1111/aas.13784 33481246

[57] Otterbeck A Skorup P Hanslin K Larsson A Stålberg J Hjelmqvist H. Intravenous anti- p. aeruginosa IgY-antibodies do not decrease pulmonary bacterial concentrations in a porcine model of ventilator-associated pneumonia. Innate Immun (2022) 28(2–8):224–34. doi: 10.1177/17534259221114217 PMC9900256 36373663

[58] Zamani K Irajian G Zahedi Bialvaei A Zahraei Salehi T Khormali M Vosough A. Passive immunization with anti- chimeric protein PilQ/PilA –DSL region IgY does not protect against mortality associated with pseudomonas aeruginosa sepsis in a rabbit model. Mol Immunol (2022) 141:258–64. doi: 10.1016/j.molimm.2021.11.021 34896925

[59] EU Clinical Trial Register. Final study report (2018). Available at: https://www.clinicaltrialsregister.eu/ctr-search/rest/download/result/attachment/2011-000801-39/1/24990.

[60] Qi Z Lu C. An IgY for a PA-MSHA bacterial strain, and preparation method and application thereof, CN102770452A. (2012).

[61] Zhang Y Zhuang Y Ma S. Pseudomonas aeruginosa resisting fab’ fragment, CN101186648A. (2007).

[62] Rongjian C Yang Y Cao H. Anti bacillus pyocyaneu vitelline immunoglobulin products and use thereof, CN1101404C. (1998).

[63] Ahmadi TS Mousavi Gargari SL Talei D. Anti-flagellin IgY antibodies protect against pseudomonas aeruginosa infection in both acute pneumonia and burn wound murine models in a non-type-specific mode. Mol Immunol (2021) 136:118–27. doi: 10.1016/j.molimm.2021.06.002 34130152

[64] Norouzi F Behrouz B Ranjbar M Mousavi Gargari SL. Immunotherapy with IgY antibodies toward outer membrane protein f protects burned mice against pseudomonas aeruginosa infection. J Immunol Res (2020) 2020:1–8. doi: 10.1155/2020/7840631 PMC7275967 32566689

[65] Michael A Meenatchisundaram S Parameswari G Subbraj T Selvakumaran S Ramalingam R. Chicken egg yolk antibodies (IgY) as an alternative to mammalian antibodies. Indian J Sci Technol (2010) 3:468–74. doi: 10.17485/ijst/2010/v3i4.24

[66] Chalghoumi R Beckers Y Portetelle D Thewis A. Hen egg yolk antibodies (IgY), production and use for passive immunization against bacterial enteric infections in chicken: a review. Presses Agronomiques de Gembloux (BE) (2009) 2:295–308.

[67] Freund J Casals J Hosmer EP. Sensitization and antibody formation after injection of tubercle bacilli and paraffin oil. Exp Biol Med (1937) 37:509–13. doi: 10.3181/00379727-37-9625

[68] Kubo N Nishii M Osada-Oka M Hatta H. A comparative study on egg yolk IgY production with different adjuvants and their inhibitory effects on staphylococcus aureus. J Poult Sci (2021) 58:192–9. doi: 10.2141/jpsa.0200062 PMC8371533 34447284

[69] Redwan EM Aljadawi AA Uversky VN. Simple and efficient protocol for immunoglobulin y purification from chicken egg yolk. Poult Sci (2021) 100:100956. doi: 10.1016/j.psj.2020.12.053 33652537 PMC7936219

[70] Polson A von Wechmar MB van Regenmortel MH. Isolation of viral IgY antibodies from yolks of immunized hens. Immunol Commun (1980) 9:475–93. doi: 10.3109/08820138009066010 7429529

[71] Tong C Geng F He Z Cai Z Ma M. A simple method for isolating chicken egg yolk immunoglobulin using effective delipidation solution and ammonium sulfate. Poult Sci (2015) 94:104–10. doi: 10.3382/PS/PEU005 25542196

[72] Bizanov G. IgY extraction and purification from chicken egg yolk. J Hell Vet Med Soc (2017) 68:265–72. doi: 10.12681/jhvms.15466

[73] Almeida MR Ferreira F Domingues P Coutinho JAP Freire MG. Towards the purification of IgY from egg yolk by centrifugal partition chromatography. Sep Purif Technol (2022) 299. doi: 10.1016/j.seppur.2022.121697

[74] Jiang X Diraviyam T Zhang X. Affinity purification of egg yolk immunoglobulins (IgY) using a human mycoplasma protein. J Chromatogr B Anal Technol Biomed Life Sci (2016) 1012–1013:37–41. doi: 10.1016/j.jchromb.2016.01.012 26807703

[75] Wiliams CM Barker JC Sims JT. Management and utilization of poultry wastes. Rev Environ Contam Toxicol (1999) 162:105–57. doi: 10.1007/978-1-4612-1528-8_3 10392043

[76] Ahmed TAE Wu L Younes M Hincke M. Biotechnological applications of eggshell: recent advances. Front Bioeng Biotechnol (2021) 9:675364. doi: 10.3389/fbioe.2021.675364 34295881 PMC8291997

[77] Shang B Wang S Lu L Ma H Liu A Zupanic A. Poultry eggshell-derived antimicrobial materials: current status and future perspectives. J Environ Manage (2022) 314:115096. doi: 10.1016/j.jenvman.2022.115096 35462255

[78] Cherian G. Chapter 16 - eggs and health: nutrient sources and supplement carriers. In: Watson AP, editor. Complementary and Alternative Therapies and the Aging Population San Diego: Elsevier Academic Press (2009). p. 333–46. R.R.B.T.-C. and A.T. doi: 10.1016/B978-0-12-374228-5.00016-0

[79] Urisu A Ando H Morita Y Wada E Yasaki T Yamada K. Allergenic activity of heated and ovomucoid-depleted egg white. J Allergy Clin Immunol (1997) 100:171–6. doi: 10.1016/s0091-6749(97)70220-3 9275136

[80] Martorell Aragonés A Boné Calvo J García Ara MC Nevot Falcó S Plaza Martín AM. Allergy to egg proteins. Allergol Immunopathol (2001) 29:72–83. doi: 10.1016/S0301-0546(01)79022-2 11420032

[81] Leow CH Xu L Harley CA Vieira-Pires RS Zhang X. Monoclonal IgY antibodies BT - IgY-technology: production and application of egg yolk antibodies: basic knowledge for a successful practice. Zhang X-Y Vieira-Pires RS Morgan PM Schade R, editors. Cham: Springer International Publishing (2021) p. 173–93. doi: 10.1007/978-3-030-72688-1_13

[82] Nishinaka S Matsuda H Murata M. Establishment of a chicken X chicken hybridoma secreting specific antibody. Int Arch Allergy Appl Immunol (1989) 89:416–9. doi: 10.1159/000234985 2793228

[83] Leow CH Xu L Harley CA Vieira-Pires RS Zhang X IgY-technology: production and application of egg yolk antibodies: basic knowledge for a successful practice. Cham, Switzerland: Springer (2021), 173–193.

[84] Seo H Hashimoto S Tsuchiya K Lin W Shibata T Ohta K. An ex vivo method for rapid generation of monoclonal antibodies (ADLib system). Nat Protoc (2006) 1:1502–6. doi: 10.1038/nprot.2006.248 17406441

[85] Seo H Masuoka M Murofushi H Takeda S Shibata T Ohta K. Rapid generation of specific antibodies by enhanced homologous recombination. Nat Biotechnol (2005) 23:731–5. doi: 10.1038/nbt1092 15924134

[86] Pitaksajjakul P Lekcharoensuk P Upragarin N Barbas CF 3rd Ibrahim MS Ikuta K. Fab MAbs specific to HA of influenza virus with H5N1 neutralizing activity selected from immunized chicken phage library. Biochem Biophys Res Commun (2010) 395:496–501. doi: 10.1016/j.bbrc.2010.04.040 20382115

[87] Lee W Syed A A Leow CY Tan SC Leow CH. Isolation and characterization of a novel anti-salbutamol chicken scFv for human doping urinalysis. Anal Biochem (2018) 555:81–93. doi: 10.1016/j.ab.2018.05.009 29775561

[88] Lee C-H Leu S-J Lee Y-C Liu C-I Lin L-T Mwale PF. Characterization of chicken-derived single chain antibody fragments against venom of naja naja atra. Toxins (Basel) (2018) 10. doi: 10.3390/toxins10100383 PMC6215181 30248928

[89] Bogen JP Grzeschik J Krah S Zielonka S Kolmar H. Rapid generation of chicken immune libraries for yeast surface display. Methods Mol Biol (2020) 2070:289–302. doi: 10.1007/978-1-4939-9853-1_16 31625102

[90] Yakhkeshi S Wu R Chelliappan B. Trends in industrialization and commercialization of IgY technology. Front Immunol (2022) 13:1–8. doi: 10.3389/fimmu.2022.991931 PMC9630564 36341353

[91] Romanowski K Clark EG Levin A Cook VJ Johnston JC. Tuberculosis and chronic kidney disease: an emerging global syndemic. Kidney Int (2016) 90:34–40. doi: 10.1016/j.kint.2016.01.034 27178832

[92] Garg RK Somvanshi DS. Spinal tuberculosis: a review. J Spinal Cord Med (2011) 34:440–54. doi: 10.1179/2045772311Y.0000000023 PMC3184481 22118251

[93] Donovan J Thwaites GE Huynh J. Tuberculous meningitis. Curr Opin Infect Dis (2020) 33:259–66. doi: 10.1097/QCO.0000000000000648 PMC7259381 32324614

[94] Xun J Qi T Zou L Tang Q Shen Y Yang J. Mycobacterium tuberculosis co-infection is associated with increased surrogate marker of the HIV reservoir. AIDS Res Ther (2020) 17:1–8. doi: 10.1186/s12981-020-00320-0 33076959 PMC7574250

[95] Shu CC Liao KM Chen YC Wang JJ Ho CH. The burdens of tuberculosis on patients with malignancy: incidence, mortality and relapse. Sci Rep (2019) 9:1–7. doi: 10.1038/s41598-019-48395-8 31417132 PMC6695428

[96] Yorke E Atiase Y Akpalu J Sarfo-Kantanka O Boima V Dey ID. The bidirectional relationship between tuberculosis and diabetes. Tuberc. Res Treat (2017) 2017:1–6. doi: 10.1155/2017/1702578 PMC5705893 29270319

[97] Gygli SM Borrell S Trauner A Gagneux S. Antimicrobial resistance in mycobacterium tuberculosis: mechanistic and evolutionary perspectives. FEMS Microbiol Rev (2017) 41:354–73. doi: 10.1093/femsre/fux011 28369307

[98] Woodworth JS Strand Clemmensen H Battey H Dijkman K Lindenstrøm T Laureano RS. A mycobacterium tuberculosis-specific subunit vaccine that provides synergistic immunity upon co-administration with bacillus calmette-guérin. Nature Commun (2021) 12. doi: 10.1038/s41467-021-26934-0 PMC8602668 34795205

[99] Zwerling A Behr MA Verma A Brewer TF Menzies D Pai M. The BCG world atlas: a database of global BCG vaccination policies and practices. PloS Med (2011) 8:e1001012. doi: 10.1371/journal.pmed.1001012 21445325 PMC3062527

[100] Shen X Xu Q Dai Z Liang H Tan N Chen Q. Tuberculosis and medicament-resistant tubercular personalized yelk polyclone antibody and method of preparing the same and applications, CN101249264A. (2008).

[101] Peleg AY Seifert H Paterson DL. Acinetobacter baumannii: emergence of a successful pathogen. Clin Microbiol Rev (2008) 21:538–82. doi: 10.1128/CMR.00058-07 PMC2493088 18625687

[102] Falagas ME Bliziotis IA Siempos II. Attributable mortality of acinetobacter baumannii infections in critically ill patients: a systematic review of matched cohort and case-control studies. Crit Care (2006) 10. doi: 10.1186/cc4869 PMC1550903 16563184

[103] Lin M-F. Antimicrobial resistance in acinetobacter baumannii: from bench to bedside. World J Clin cases (2014) 2:787. doi: 10.12998/wjcc.v2.i12.787 25516853 PMC4266826

[104] Sopirala MM Mangino JE Gebreyes WA Biller B Bannerman T Balada-Llasat JM. Synergy testing by etest, microdilution checkerboard, and time-kill methods for pan-drug-resistant acinetobacter baumannii. Antimicrob Agents Chemother (2010) 54:4678–83. doi: 10.1128/AAC.00497-10 PMC2976112 20713678

[105] Zheng Y Xu N Pang J Han H Yang H Qin W. Colonization with extensively drug-resistant acinetobacter baumannii and prognosis in critically ill patients: an observational cohort study. Front Med (2021) 8:667776. doi: 10.3389/fmed.2021.667776 PMC8119758 33996866

[106] Lee CR Lee JH Park M Park KS Bae IK Kim YB. Biology of acinetobacter baumannii: pathogenesis, antibiotic resistance mechanisms, and prospective treatment options. Front Cell Infect Microbiol (2017) 7:55. doi: 10.3389/fcimb.2017.00055 28348979 PMC5346588

[107] Nielsen TB Pantapalangkoor P Luna BM Bruhn KW Yan J Dekitani K. Monoclonal antibody protects against acinetobacter baumannii infection by enhancing bacterial clearance and evading sepsis. J Infect Dis (2017) 216:489–501. doi: 10.1093/infdis/jix315 28931235 PMC5853763

[108] Nielsen TB Yan J Slarve M Lu P Li R Ruiz J. Monoclonal antibody therapy against acinetobacter baumannii. Infect Immun (2021) 89. doi: 10.1128/IAI.00162-21 PMC8445169 34310884

[109] Yeganeh O Shabani M Pakzad P Mosaffa N Hashemi A. Evaluation the reactivity of a peptide-based monoclonal antibody derived from OmpA with drug resistant pulsotypes of acinetobacter baumannii as a potential therapeutic approach. Ann Clin Microbiol Antimicrob (2022) 21:1–13. doi: 10.1186/s12941-022-00523-5 35773688 PMC9245400

[110] Jahangiri A Owlia P Rasooli I Salimian J Derakhshanifar E Aghajani Z. Specific egg yolk immunoglobulin as a promising non-antibiotic biotherapeutic product against acinetobacter baumannii pneumonia infection. Sci Rep (2021) 11:1–11. doi: 10.1038/s41598-021-81356-8 33479293 PMC7820402

[111] Reynolds D Kollef M. The epidemiology and pathogenesis and treatment of pseudomonas aeruginosa infections: an update. Drugs (2021) 81:2117–31. doi: 10.1007/s40265-021-01635-6 PMC8572145 34743315

[112] Nilsson E Larsson A Olesen HV Wejåker PE Kollberg H. Good effect of IgY against pseudomonas aeruginosa infections in cystic fibrosis patients. Pediatr Pulmonol (2008) 43:892–9. doi: 10.1002/ppul.20875 18680179

[113] Alibek K Bekmurzayeva A Mussabekova A Sultankulov B. Using antimicrobial adjuvant therapy in cancer treatment: a review. Infect Agent. Cancer (2012) 7:33. doi: 10.1186/1750-9378-7-33 23164412 PMC3637577

[114] Akita EM Nakai S. Production and purification of fab′ fragments from chicken egg yolk immunoglobulin y (IgY). J Immunol Methods (1993) 162:155–64. doi: 10.1016/0022-1759(93)90380-P 8315286

[115] Hooi JKY NS Lai WY Ng WK Suen MMY Underwood FE Tanyingoh D. Global prevalence of helicobacter pylori infection: systematic review and meta-analysis. Gastroenterology (2017) 153:420–9. doi: 10.1053/j.gastro.2017.04.022 28456631

[116] Savoldl A Carrara E Graham D Conti M Tacconelli E. Prevalence of antibiotic resistance in helicobcater pylori: revisón sistemática y metaanálisis en las regiones de la OMS. Gastroenterology (2018) 155:1372–82. doi: 10.1053/j.gastro.2018.07.007.Prevalence PMC6905086 29990487

[117] Tacconelli MN Carrara E Savoldi A Harbarth S Mendelson M Monnet DL. Discovery, research, and development of new antibiotics: the WHO priority list of antibiotic-resistant bacteria and tuberculosis. Lancet Infect Dis (2018) 18:318–27. doi: 10.1016/S1473-3099(17)30753-3 29276051

[118] Mestre A Sathiya Narayanan R Rivas D John J Abdulqader MA Khanna T. Role of probiotics in the management of helicobacter pylori. Cureus (2022) 14. doi: 10.7759/cureus.26463 PMC9338786 35919364

[119] Dos Santos Viana I Cordeiro Santos ML Santos Marques H Lima de Souza Gonçalves V Bittencourt de Brito B França da Silva FA. Vaccine development against helicobacter pylori: from ideal antigens to the current landscape. Expert Rev Vaccines (2021) 20:989–99. doi: 10.1080/14760584.2021.1945450 34139141

[120] Sousa C Ferreira R Azevedo NF Oleastro M Azeredo J Figueiredo C. Helicobacter pylori infection: from standard to alternative treatment strategies. Crit Rev Microbiol (2022) 48:376–96. doi: 10.1080/1040841X.2021.1975643 34569892

[121] Shin JH Yang M Nam SW Kim JT Myung NH Bang WG. Use of egg yolk-derived immunoglobulin as an alternative to antibiotic treatment for control of helicobacter pylori infection. Clin Diagn Lab Immunol (2002) 9:1061–6. doi: 10.1128/CDLI.9.5.1061-1066.2002 PMC120060 12204960

[122] Horie K Horie N Abdou AM Yang JO Yun SS Chun HN. Suppressive effect of functional drinking yogurt containing specific egg yolk immunoglobulin on helicobacter pylori in humans. J Dairy Sci (2004) 87:4073–9. doi: 10.3168/jds.S0022-0302(04)73549-3 15545368

[123] Yang Y-H Park D Yang G Lee SH Bae DK Kyung J. Anti- helicobacter pylori effects of IgY from egg york of immunized hens. Lab Anim Res (2012) 28:55. doi: 10.5625/lar.2012.28.1.55 22474475 PMC3315199

[124] Wang B Yang J Cao S Wang H Pan X Zhu J. Preparation of specific anti-helicobacter pylori yolk antibodies and their antibacterial effects. Int J Clin Exp Pathol (2014) 7:6430–7. PMC4230139 25400721

[125] Solhi R Alebouyeh M Khafri A Rezaeifard M. Microbial pathogenesis *In vitro* evaluation of cross-strain inhibitory effects of IgY polyclonal antibody against h . pylori. Microb Pathog (2017) 110:682–7. doi: 10.1016/j.micpath.2017.03.025 28351713

[126] Xie R Ye Q. Additive containing anti-helicobacter pylori duck yolk antibody, CN109453372A. (2018).

[127] Li Y Duan S Yu H Wang H Chen Y Ding S. Gastric floating agent for treating helicobacter pylori infection, CN114081944A. (2021).

[128] Jixiang C Weimin G. Floating tablet preparation of yolk immunoglobulin IgY for preventing and treating human gastric diseases, CN102370626A. (2010).

[129] Cho GS Cho HJ Jun JH Oh HG Sa GJ. Functional pudding and method for preparing thereof, KR100785666B1. (2001).

[130] Cho GS Cho HJ Jun JH Oh HG Sa GJ. Functional soybean milk and method for preparing thereof, KR20030012560A. (2001).

[131] Zhang X. Breath freshening toothpaste capable of efficiently preventing and controlling helicobacter pylori and preparation method thereof, CN110507583A. (2019).

[132] Shin JH Nam SW Kim JT Yoon JB Bang WG Roe IH. Identification of immunodominant helicobacter pylori proteins with reactivity to H. pylori-specific egg-yolk immunoglobulin. J Med Microbiol (2003) 52:217–22. doi: 10.1099/jmm.0.04978-0 12621086

[133] Zhai K Gong Y Sun L He L Xue Z Yang Y. DNA Starvation / stationary phase protection protein of helicobacter pylori as a potential immunodominant antigen for infection detection. Helicobacter (2023) 28(2):1–10. doi: 10.1111/hel.12955 36775815

[134] Zeng J Xie C Zhang L Liu X Chan MTV Wu WKK. Host cell antimicrobial responses against helicobacter pylori infection: from biological aspects to therapeutic strategies. Int J Mol Sci (2022) 23. doi: 10.3390/ijms231810941 PMC9504325 36142852

[135] Baj J Forma A Sitarz M Portincasa P Garruti G Krasowska D. Helicobacter pylori virulence factors - mechanisms of bacterial pathogenicity in the gastric microenvironment. Cells (2021) 10(1):1–37. doi: 10.3390/cells10010027 PMC7824444 33375694

[136] Torres J Camorlinga-ponce M Perez-perez G. Specific serum immunoglobulin G response to urease and CagA antigens of helicobacter pylori in infected children and adults in a country with high prevalence of infection. Clin Diagn Lab Immunol (2002) 9:97–100. doi: 10.1128/CDLI.9.1.97 11777836 PMC119889

[137] Nomura S Suzuki H Masaoka T Kurabayashi K Ishii H Kitajima M. Effect of dietary anti-urease immunoglobulin y on helicobacter pylori infection in Mongolian gerbils. Helicobacter (2005) 10:43–52. doi: 10.1111/j.1523-5378.2005.00290.x 15691314

[138] Suzuki H HT Nomura S Masaoka T Goshima H Kamata N Kodama Y. Effect of dietary anti-helicobacter pylori-urease immunoglobulin y on helicobacter pylori infection. Aliment Pharmacol Ther (2004) 20:185–92. doi: 10.1111/j.1365-2036.2004.02027.x 15298626

[139] Malekshahi ZV Latif S Gargari M Rasooli I Ebrahimizadeh W. Microbial pathogenesis treatment of helicobacter pylori infection in mice with oral administration of egg yolk-driven anti-UreC immunoglobulin. Microb Pathog (2011) 51:366–72. doi: 10.1016/j.micpath.2011.06.002 21803146

[140] Shin J Roe I Kim H Kim H. Production of anti-helicobacter pylori urease- specific immunoglobulin in egg yolk using an antigenic epitope of H. pylori urease. J Med Microbiol (2004) 53(Pt 1):31–4. doi: 10.1099/jmm.0.05327-0 14663102

[141] Kazimierczuk K Cova L Ndeboko B Szczyrk U Brzozowski T Sirko A. Genetic immunization of ducks for production of antibodies specific to helicobacter pylori UreB in egg yolks. Acta Biochim Pol (2005) 52:261–6. doi: 10.18388/abp.2005_3517 15827623

[142] Cova L Kazimierczuk K Koprowski H Sirko A Trepo C Ostoja-Zagórski W. Poly-clone antibodies against helicobacter pylori proteins as well as method for their production, PL201982B1. (2003).

[143] Aiba Y Umeda K Rahman S Nguyen SV Komatsu Y. Synergistic effect of anti- helicobacter pylori urease immunoglobulin y from egg yolk of immunized hens and lactobacillus johnsonii no . 1088 to inhibit the growth of helicobacter pylori *in vitro* and *in vivo* . Vaccine (2019) 37:3106–12. doi: 10.1016/j.vaccine.2019.04.045 31031029

[144] Aiba Y KY Nakano Y Koga Y Takahashi K. A highly acid-resistant novel strain of lactobacillus johnsonii no. 1088 has antibacterial activity, including that against helicobacter pylori, and inhibits gastrin-mediated acid production in mice. Microbiologyopen (2015) 4:465–74. doi: 10.1002/mbo3.252 PMC4475388 25771812

[145] Aiba Y Ishikawa H Tokunaga M Komatsu Y. Anti- helicobacter pylori activity of non-living , heat-killed form of lactobacilli including lactobacillus johnsonii no. 1088. FEMS Microbiol Lett (2017) 364(11):1–5. doi: 10.1093/femsle/fnx102 28505287

[146] Mony TJ Kwon H Won M Kang Y Lee H Kim S. Anti-urease immunoglobulin ( IgY ) from egg yolk prevents helicobacter pylori infection in a mouse model. Food Agric Immunol (2019) 0105:662–76. doi: 10.1080/09540105.2019.1617251

[147] Gandhi S Alshehri SM. Molecular stability of the rabbit and chicken egg yolk immunoglobulins. Front Biosci (2021) 13:185–94. doi: 10.2741/877 33048781

[148] Yoshikatsu K Nobutake K. Pharmaceutical composition comprising anti-h.pylori urease IgY antibodies and an inhibitor of gastric acid secretion, EP1172116A1. (2000).

[149] Schetters STT Jong WSP Kruijssen LJW Van Saparoea H.B.V.D.B. Engels S. Bacterial inclusion bodies function as vehicles for dendritic cell-mediated T cell responses. Cell Mol Immunol (2020) 17(4):2019–21. doi: 10.1038/s41423-019-0298-x PMC7109023 31595053

[150] Kesik M Saczyńska V Szewczyk B Płucienniczak A. Inclusion bodies from recombinant bacteria as a novel system for delivery of vaccine antigen by the oral route. Immunol Lett (2004) 91:197–204. doi: 10.1016/j.imlet.2003.12.001 15019290

[151] Van Beek LF Langereis JD Van Saparoea H.B.V.D.B. Gillard J Jong WSP Van Opzeeland FJ. Intranasal vaccination with protein bodies elicit strong protection against streptococcus pneumoniae colonization. Vaccine (2021) 39:6920–9. doi: 10.1016/j.vaccine.2021.10.006 34696934

[152] Chen S Sandford S Kirman J Rehm BHA. Design of bacterial inclusion bodies as antigen carrier systems. Adv Biosyst (2018) 1800118:1–13. doi: 10.1002/adbi.201800118

[153] Zhang J Qian J Zhang X. Outer membrane inflammatory protein a , a new virulence factor involved in the pathogenesis of helicobacter pylori. Mol Biol Rep (2014) 41:7807–14. doi: 10.1007/s11033-014-3673-9 25096514

[154] Borhani K Mobarez AM Khabiri AR Behmanesh M Khoramabadi N. Production of specific IgY helicobacter pylori recombinant OipA protein and assessment of its inhibitory effects towards attachment of h . pylori to AGS cell line. Clin Exp Vaccine Res (2015) 4:177–83. doi: 10.7774/cevr.2015.4.2.177 PMC4524902 26273576

[155] Abdullah M Gre LK Bronte-tinkew D Capurro MI Rizzuti D Jones NL. VacA promotes CagA accumulation in gastric epithelial cells during H. pylori infection. Sci Rep (2019) 9(1):1–9. doi: 10.1038/s41598-018-37095-4 30631092 PMC6328614

[156] Sook K Ki M Ullah HMA Lee E Deuk Y Chung M. Preventive effect of anti-VacA egg yolk immunoglobulin ( IgY ) on helicobacter pylori -infected mice. Vaccine (2018) 36:371–80. doi: 10.1016/j.vaccine.2017.11.082 29223485

[157] Jeong KS Ki MR Hong KS Lee HR Goo MJ Han JY. A method for preparing neutral antibody of anti-helicobacter pylori and foods containing the antibody, KR101377697B1. (2008).

[158] Borhani K Mohabati A Reza A Behmanesh M Khoramabadi N. Microbial pathogenesis inhibitory effects of rHP-NAP IgY against helicobacter pylori attachment to AGS cell line. Microb Pathog (2016) 97:231–5. doi: 10.1016/j.micpath.2016.06.004 27265677

[159] Attallah AM Abbas AT. Efficacy of passive immunization with IgY antibodies to a 58-kDa h . pylori antigen on severe gastritis in BALB / c mouse model. J Immunoass Immunochem (2009) 30:359–77. doi: 10.1080/15321810903187922 19739011

[160] Peng J Lu C Qi Z. Anti-helicobacter pylori FlaA protein antibody IgY, preparation method and application thereof, CN103360491A. (2013).

[161] Schade R Terzolo HR. (2006). IgY-technology: application and trends. In: EPC 2006-12th European poultry conference, Verona, Italy, 10-14 Sept. 2006. World's Poultry Science Association (WPSA).

[162] Yoshikatsu K Faustino IC Nobutake K Ariga M. Specific antibodies for use in preparation of pharmaceutical compositions useful in the prevention or treatment of gastritis, gastric ulcers and duodenal ulcers, US2001021393A1. (1997).

[163] Saracino IM Pavoni M Saccomanno L Fiorini G Pesci V Foschi C. Antimicrobial efficacy of five probiotic strains against helicobacter pylori. Antibiotics (2020) 9:244. doi: 10.3390/antibiotics9050244 32403331 PMC7277513

[164] Seong HC Jun LJ Jin BY Soo KH. Food containing active strains for inhibiting infection and treating gastritis, gastric and duodenal ulcers, US2002037341A1. (1999).

[165] Wan Z Wan J Ma Y. Chicken IgY bifunctional antibody for treating helicobacter pylori, CN111228481A. (2018).

[166] Baah S Laws M. Antibody – drug conjugates a tutorial review. Molecules (2021) 26(10). doi: 10.3390/molecules26102943 PMC8156828 34063364

[167] Taillieu E Chiers K Amorim I Gärtner F Maes D Van Steenkiste C. Gastric helicobacter species associated with dogs, cats and pigs: significance for public and animal health. Vet Res (2022) 53:42. doi: 10.1186/s13567-022-01059-4 35692057 PMC9190127

[168] Nasiri K Zibaee S Nassiri M Tahmoorespur M Haghparast A. Production of specific IgY antibody to the recombinant FanC protein produced in escherichia coli. Iran J Basic Med Sci (2016) 19:883–9. doi: 10.22038/ijbms.2016.7471 PMC5048125 27746871

[169] Amaral JA Tino De Franco M Carneiro-Sampaio MMS Carbonare SB. Anti-enteropathogenic escherichia coli immunoglobulin y isolated from eggs laid by immunised leghorn chickens. Res Vet Sci (2002) 72:229–34. doi: 10.1053/rvsc.2002.0551 12076119

[170] De Almeida CMC Quintana-Flores VM Medina-Acosta E Schriefer A Barral-Netto M Dias Da Silva W. Egg yolk anti-BfpA antibodies as a tool for recognizing and identifying enteropathogenic escherichia coli. Scand J Immunol (2003) 57:573–82. doi: 10.1046/j.1365-3083.2003.01243.x 12791096

[171] Melo AR Lasunskaia EB De Almeida CMC Schriefer A Kipnis TL Dias Da Silva W. Expression of the virulence factor, BfpA, by enteropathogenic escherichia coli is essential for apoptosis signalling but not for NF-κB activation in host cells. Scand J Immunol (2005) 61:511–9. doi: 10.1111/j.1365-3083.2005.01626.x 15963045

[172] Mwale PF Lee CH Leu SJ Lee YC Wu HH Lin LT. Antigenic epitopes on the outer membrane protein a of escherichia coli identified with single-chain variable fragment (scFv) antibodies. Appl Microbiol Biotechnol (2019) 103:5285–99. doi: 10.1007/s00253-019-09761-8 31028439

[173] Girard F Batisson I Martinez G Breton C Harel J Fairbrother JM. Use of virulence factor-specific egg yolk-derived immunoglobulins as a promising alternative to antibiotics for prevention of attaching and effacing escherichia coli infections. FEMS Immunol Med Microbiol (2006) 46:340–50. doi: 10.1111/j.1574-695X.2005.00030.x 16553806

[174] Wang Q Hou XJ Cai K Li T Liu YN Tu W. Passive protection of purified yolk immunoglobulin administered against shiga toxin 1 in mouse models. Can J Microbiol (2010) 56:1003–10. doi: 10.1139/W10-087 21164570

[175] Neri P Tokoro S Kobayashi R Sugiyama T Umeda K Shimizu T. Specific egg yolk immunoglobulin as a new preventive approach for shiga-toxin-mediated diseases. PloS One (2011) 6. doi: 10.1371/journal.pone.0026526 PMC3197529 22028896

[176] Feng Y Liu W Shi D. Effectiveness of egg yolk antibody against shiga toxin ii variant toxicity *in vitro* and *in vivo* . Curr Microbiol (2013) 67:448–53. doi: 10.1007/s00284-013-0384-8 23689941

[177] Ma Z Kang M Meng S Tong Z Do Yoon S Jang Y. Selective killing of shiga toxin-producing escherichia coli with antibody-conjugated chitosan nanoparticles in the gastrointestinal tract. ACS Appl Mater Interfaces (2020) 12:18332–41. doi: 10.1021/acsami.0c02177 32239905

[178] Zhen YH Fang R Ding C Jin LJ Li XY Diao YP. Efficacy of specific IgY for treatment of lipopolysaccharide-induced endotoxemia using a mouse model. J Appl Microbiol (2011) 111:1524–32. doi: 10.1111/j.1365-2672.2011.05155.x 21933310

[179] You J Xu Y Li H Wang L Wu F Xu F. Chicken egg yolk immunoglobulin (IgY) developed against fusion protein LTB-STa-STb neutralizes the toxicity of escherichia coli heat-stable enterotoxins. J Appl Microbiol (2014) 117:320–8. doi: 10.1111/jam.12525 24750381

[180] Han S Yu H Yang F Qiao S He P. Effect of dietary supplementation with hyperimmunized hen egg yolk powder on diarrhoea incidence and intestinal health of weaned pigs. Food Agric Immunol (2019) 30:333–48. doi: 10.1080/09540105.2019.1581732

[181] Han S Wen Y Yang F He P. Chicken egg yolk antibody (IgY) protects mice against enterotoxigenic escherichia coli infection through improving intestinal health and immune response. Front Cell Infect Microbiol (2021) 11:662710. doi: 10.3389/fcimb.2021.662710 33928047 PMC8076637

[182] Tan X Li J Li Y Li J Wang Q Fang L. Effect of chicken egg yolk immunoglobulins on serum biochemical profiles and intestinal bacterial populations in early-weaned piglets. J Anim Physiol Anim Nutr (Berl) (2019) 103:1503–11. doi: 10.1111/jpn.13129 PMC7166376 31144409

[183] Vega CG Bok M Ebinger M Rocha LA Rivolta AA González Thomas V. A new passive immune strategy based on IgY antibodies as a key element to control neonatal calf diarrhea in dairy farms. BMC Vet Res (2020) 16:1–9. doi: 10.1186/s12917-020-02476-3 32727468 PMC7388481

[184] Karamzadeh-Dehaghani A Towhidi A Zhandi M Mojgani N Fouladi-Nashta A. Combined effect of probiotics and specific immunoglobulin y directed against escherichia coli on growth performance, diarrhea incidence, and immune system in calves. Animal (2021) 15:100124. doi: 10.1016/j.animal.2020.100124 33573946

[185] Kariyawasam S Wilkie BN Gyles CL. Resistance of broiler chickens to escherichia coli respiratory tract infection induced by passively transferred egg-yolk antibodies. Vet Microbiol (2004) 98:273–84. doi: 10.1016/j.vetmic.2003.10.022 15036536

[186] Mahdavi AH Rahmani HR Nili N Samie AH Soleimanian-Zad S Jahanian R. Effects of dietary egg yolk antibody powder on growth performance, intestinal escherichia coli colonization, and immunocompetence of challenged broiler chicks. Poult Sci (2010) 89:484–94. doi: 10.3382/ps.2009-00541 20181864

[187] Nash P Robinson DL Rosevear JW. Immunogen adherence and method of making and using same, WO03061693A1. (2017).

[188] Peng S Wang J Pan R Jiang Z. Egg yolk antibody anti-human enterotoxigenic escherichia coli adhesion protein and application thereof, CN103409455A. (2013).

[189] Parma YR Chacana PA Rogé A Kahl A Cangelosi A Geoghegan P. Antibodies anti-shiga toxin 2 b subunit from chicken egg yolk: isolation, purification and neutralization efficacy. Toxicon (2011) 58:380–8. doi: 10.1016/j.toxicon.2011.07.009 PMC7111861 21803069

[190] Fathi J Ebrahimi F Nazarian S Hajizade A Malekzadegan Y Abdi A. Production of egg yolk antibody (IgY) against shiga-like toxin (stx) and evaluation of its prophylaxis potency in mice. Microb Pathog (2020) 145. doi: 10.1016/j.micpath.2020.104199 32320733

[191] Wang H Hou X Wang Q Bao S Cai K Shi J. Anti-I type shiga toxin IgY antibody as well as preparation method and use thereof, CN101570574A. (2009).

[192] Chalghoumi R Théwis A Portetelle D Beckers Y. Production of hen egg yolk immunoglobulins simultaneously directed against salmonella enteritidis and salmonella typhimurium in the same egg yolk. Poult Sci (2008) 87:32–40. doi: 10.3382/ps.2007-00252 18079447 PMC7107053

[193] Esmailnejad A Abdi-Hachesoo B Hosseini Nasab E Shakoori M. Production, purification, and evaluation of quail immunoglobulin y against salmonella typhimurium and salmonella enteritidis. Mol Immunol (2019) 107:79–83. doi: 10.1016/j.molimm.2019.01.012 30665061 PMC7112669

[194] Chalghoumi R Théwis A Beckers Y Marcq C Portetelle D Schneider YJ. Adhesion and growth inhibitory effect of chicken egg yolk antibody (IgY) on salmonella enterica serovars enteritidis and typhimurium *in vitro* . Foodborne Pathog Dis (2009) 6:593–604. doi: 10.1089/fpd.2008.0258 19388827

[195] Chalghoumi R Marcq C Théwis A Portetelle D Beckers Y. Effects of feed supplementation with specific hen egg yolk antibody (immunoglobin y) on salmonella species cecal colonization and growth performances of challenged broiler chickens. Poult Sci (2009) 88:2081–92. doi: 10.3382/ps.2009-00173 19762860

[196] Li X Yao Y Wang X Zhen Y Thacker PA Wang L. Chicken egg yolk antibodies (IgY) modulate the intestinal mucosal immune response in a mouse model of salmonella typhimurium infection. Int Immunopharmacol (2016) 36:305–14. doi: 10.1016/j.intimp.2016.04.036 PMC7106048 27214338

[197] Al-Adwani SR Crespo R Shah DH. Production and evaluation of chicken egg-yolk-derived antibodies against campylobacter jejuni colonization-associated proteins. Foodborne Pathog Dis (2013) 10:624–31. doi: 10.1089/fpd.2012.1313 23742296

[198] Paul NC Al-Adwani S Crespo R Shah DH. Evaluation of passive immunotherapeutic efficacy of hyperimmunized egg yolk powder against intestinal colonization of campylobacter jejuni in chickens. Poult Sci (2014) 93:2779–87. doi: 10.3382/ps.2014-04234 25214556

[199] Hermans D Van Steendam K Verbrugghe E Verlinden M Martel A Seliwiorstow T. Passive immunization to reduce campylobacter jejuni colonization and transmission in broiler chickens. Vet Res (2014) 45:1–12. doi: 10.1186/1297-9716-45-27 24589217 PMC3996517

[200] Thibodeau A Fravalo P Perron A Lewandowski SL Letellier A. Production and characterization of anti-campylobacter jejuni IgY derived from egg yolks. Acta Vet Scand (2017) 59:1–9. doi: 10.1186/s13028-017-0346-4 29208016 PMC5717825

[201] Garba AS Thibodeau A Perron A Laurent-Lewandowski S Letellier A Fravalo P. *In vitro* efficacy of potentiated egg yolk powder against campylobacter jejuni does not correlate with *in vitro* efficacy. PloS One (2019) 14:1–17. doi: 10.1371/journal.pone.0212946 PMC6405129 30845147

[202] Vandeputte J Martel A Canessa S Van Rysselberghe N De Zutter L Heyndrickx M. Reducing campylobacter jejuni colonization in broiler chickens by in-feed supplementation with hyperimmune egg yolk antibodies. Sci Rep (2019) 9:1–10. doi: 10.1038/s41598-019-45380-z 31222043 PMC6586802

[203] Wang H Zeng X Cao L He Q Lin J. Passive immunization of chickens with anti-enterobactin egg yolk powder for campylobacter control. Vaccines (2021) 9:1–12. doi: 10.3390/vaccines9060569 PMC8230082 34205835

[204] Zeng X Wang H Huang C Logue CM Barbieri NL Nolan LK. Evaluation of the immunogenic response of a novel enterobactin conjugate vaccine in chickens for the production of enterobactin-specific egg yolk antibodies. Front Immunol (2021) 12:629480. doi: 10.3389/fimmu.2021.629480 33868248 PMC8050339

[205] Mulvey GL Dingle TC Fang L Strecker J Armstrong GD. Therapeutic potential of egg yolk antibodies for treating clostridium difficile infection. J Med Microbiol (2011) 60:1181–7. doi: 10.1099/jmm.0.029835-0 21474614

[206] Pizarro-Guajardo M Díaz-González F Álvarez-Lobos M Paredes-Sabja D. Characterization of chicken IgY specific to clostridium difficile R20291 spores and the effect of oral administration in mouse models of initiation and recurrent disease. Front Cell Infect Microbiol (2017) 7:365. doi: 10.3389/fcimb.2017.00365 28856119 PMC5557795

[207] Borody TJ. Therapy for enteric infections, WO2011036539A1. (2011).

[208] Bachtiar EW Bachtiar BM Soejoedono RD Wibawan IW Afdhal A. Biological and immunogenicity property of IgY anti s. mutans ComD. Open Dent J (2016) 10:308–14. doi: 10.2174/1874210601610010308 PMC4911422 27386013

[209] Chen X Yang B Qi C Sun TW Chen F Wu J. DNA-Templated microwave-hydrothermal synthesis of nanostructured hydroxyapatite for storing and sustained release of an antibacterial protein. Dalt Trans (2016) 45:1648–56. doi: 10.1039/c5dt03357h 26696032

[210] Yan Y Guan Y Luo L Lu B Chen F Jiang B. Effects of immunoglobulin y-loaded amorphous calcium phosphate on dentinal tubules occlusion and antibacterial activity. Front Bioeng Biotechnol (2022) 10:921336. doi: 10.3389/fbioe.2022.921336 36246386 PMC9554463

[211] Smith DJ King WF Godiska R. Passive transfer of immunoglobulin y antibody to streptococcus mutans glucan binding protein b can confer protection against experimental dental caries. Infect Immun (2001) 69:3135–42. doi: 10.1128/IAI.69.5.3135-3142.2001 PMC98269 11292733

[212] Krüger C Pearson SK Kodama Y Vacca Smith A Bowen WH Hammarström L. The effects of egg-derived antibodies to glucosyltransferases on dental caries in rats. Caries Res (2004) 38:9–14. doi: 10.1159/000073914 14684971

[213] Bachtiar EW Soejoedono RD Bachtiar BM Henrietta A Farhana N Yuniastuti M. Effects of soybean milk, chitosan, and anti-streptococcus mutans IgY in malnourished rats’ dental biofilm and the IgY persistency in saliva. Interv Med Appl Sci (2015) 7:118–23. doi: 10.1556/1646.7.2015.3.6 PMC4609024 26525071

[214] Bachtiar EW Afdhal A Meidyawati R Soejoedono RD Poerwaningsih E. Effect of topical anti-streptococcus mutans IgY gel on quantity of s. mutans on rats’ tooth surface. Acta Microbiol Immunol Hung (2016) 63:159–69. doi: 10.1556/030.63.2016.2.2 27352970

[215] Nguyen SV Icatlo FC Nakano T Isogai E Hirose K Mizugai H. Anti–cell-associated glucosyltransferase immunoglobulin y suppression of salivary mutans streptococci in healthy young adults. J Am Dent Assoc (2011) 142:943–9. doi: 10.14219/jada.archive.2011.0301 21804061

[216] Jain RL Tandon S Rai TS Mathur R Soni KK Rawat M. A comparative evaluation of xylitol chewing gum and a combination of IgY + xylitol chewable tablet on salivary streptococcus mutans count in children: a double-blind randomized controlled trial. Int J Clin Pediatr Dent (2022) 15:S212–20. doi: 10.5005/jp-journals-10005-2162 PMC9108843 35645521

[217] An M Wang Y Zhang Z Wang W. Anti-caries chewable tablet and preparation method thereof, CN111227090A. (2020).

[218] Zhao K. Immunoglobulin (IgY) antibody oral spray for preventing decayed teeth, CN102000333A. (2010).

[219] Zhihai LY. A microcapsule of egg yolk immunoglobulin, preparing process and use thereof, CN101007169A. (2006).

[220] Xu FX Xu YP Jin LJ Liu H Wang LH You JS. Effectiveness of egg yolk immunoglobulin (IgY) against periodontal disease-causing fusobacterium nucleatum. J Appl Microbiol (2012) 113:983–91. doi: 10.1111/j.1365-2672.2012.05396.x 22789022

[221] Wang F Qiao W Bao B Wang S Mac Regenstein J Shi Y. Effect of IgY on periodontitis and halitosis induced by fusobacterium nucleatum. J Microbiol Biotechnol (2019) 29:311–20. doi: 10.4014/jmb.1810.10044 30609885

[222] Li X He P Yu L He Q Jia C Yang H. Production and characteristics of a novel chicken egg yolk antibody (IgY) against periodontitis-associated pathogens. J Oral Microbiol (2020) 12. doi: 10.1080/20002297.2020.1831374 PMC7580850 33144924

[223] Tezuka A Hamajima S Hatta H Abiko Y. Inhibition of porphyromonas gingivalis hemagglutinating activity by IgY against a truncated HagA. J Oral Sci (2006) 48:227–32. doi: 10.2334/josnusd.48.227 17220621

[224] Hamajima S Maruyama M Hijiya T Hatta H Abiko Y. Egg yolk-derived immunoglobulin (IgY) against porphyromonas gingivalis 40-kDa outer membrane protein inhibits coaggregation activity. Arch Oral Biol (2007) 52:697–704. doi: 10.1016/j.archoralbio.2006.12.013 17275778

[225] Yokoyama K Sugano N Rahman AKMS Oshikawa M Ito K. Activity of anti-porphyromonas gingivalis egg yolk antibody against gingipains *in vitro* . Oral Microbiol Immunol (2007) 22:352–5. doi: 10.1111/j.1399-302X.2007.00358.x 17803634

[226] Yokoyama K Sugano N Shimada T Shofiqur RAKM Ibrahim ESM Isoda R. Effects of egg yolk antibody against porphyromonas gingivalis gingipains in periodontitis patients. J Oral Sci (2007) 49:201–6. doi: 10.2334/josnusd.49.201 17928726

[227] Nguyen SV Nguyen MTH Tran BC Ho MTQ Umeda K Rahman S. Evaluation of lozenges containing egg yolk antibody against porphyromonas gingivalis gingipains as an adjunct to conventional non-surgical therapy in periodontitis patients: a randomized controlled clinical trial. J Periodontol (2018) 89:1334–9. doi: 10.1002/JPER.18-0037 30043979

[228] Xu Y Selerio-Poely T Ye X. Clinical and microbiological effects of egg yolk antibody against porphyromonas gingivalis as an adjunct in the treatment of moderate to severe chronic periodontitis: a randomized placebo-controlled clinical trial. J Periodontal Implant Sci (2018) 48:47–59. doi: 10.5051/jpis.2018.48.1.47 29535890 PMC5841267

[229] Jiang D Luo Q Shen J Xu Y. Method for preparing yolk immunoglobulin vaccine for resisting porphyromonas gingivalis, CN101791405A. (2010).

[230] Xu Y Liu X Gui S Han X Xu H Wang T. Specific anti-porphyromonas gingivalis egg yolk antibody liposome solution and preparation method thereof, CN112274638A. (2019).

[231] Zhen YH Jin LJ Guo J Li XY Li Z Fang R. Characterization of specific egg yolk immunoglobulin (IgY) against mastitis-causing staphylococcus aureus. J Appl Microbiol (2008) 105:1529–35. doi: 10.1111/j.1365-2672.2008.03920.x 19146490

[232] Kota RK Reddy PN Sreerama K. Application of IgY antibodies against staphylococcal protein a (SpA) of staphylococcus aureus for detection and prophylactic functions. Appl Microbiol Biotechnol (2020) 104:9387–98. doi: 10.1007/s00253-020-10912-5 32960294

[233] Guimarães MCC Amaral LG Rangel LBA Silva IV Matta CGF De Matta MFR. Growth inhibition of staphylococcus aureus by chicken egg yolk antibodies. Arch Immunol Ther Exp (Warsz) (2009) 57:377–82. doi: 10.1007/s00005-009-0041-x 19693650

[234] Wang LH Li XY Jin LJ You JS Zhou Y Li SY. Characterization of chicken egg yolk immunoglobulins (IgYs) specific for the most prevalent capsular serotypes of mastitis-causing staphylococcus aureus. Vet Microbiol (2011) 149:415–21. doi: 10.1016/j.vetmic.2010.11.029 21168286

[235] Zhen YH Jin LJ Li XY Guo J Li Z Zhang BJ. Efficacy of specific egg yolk immunoglobulin (IgY) to bovine mastitis caused by staphylococcus aureus. Vet Microbiol (2009) 133:317–22. doi: 10.1016/j.vetmic.2008.07.016 18774241

[236] Wu D Ding Y Yao K Gao W Wang Y. Antimicrobial resistance analysis of clinical escherichia coli isolates in neonatal ward. Front Pediatr (2021) 9:670470. doi: 10.3389/fped.2021.670470 34113589 PMC8185016

[237] Poirel L Madec J-Y Lupo A Schink A-K Kieffer N Nordmann P. Antimicrobial resistance in escherichia coli. Microbiol Spectr (2018) 6:979–80. doi: 10.1128/microbiolspec.ARBA-0026-2017 PMC11633601 30003866

[238] Rangel JM Sparling PH Crowe C Griffin PM Swerdlow DL. Epidemiology of escherichia coli O157:H7 outbreaks, united states, 1982–2002. Emerg Infect Dis (2005) 11:603–9. doi: 10.3201/eid1104.040739 PMC3320345 15829201

[239] Fröhlicher E Krause G Zweifel C Beutin L Stephan R. Characterization of attaching and effacing escherichia coli (AEEC) isolated from pigs and sheep. BMC Microbiol (2008) 8:1–6. doi: 10.1186/1471-2180-8-144 18786265 PMC2571105

[240] Cook SR Maiti PK DeVinney R Allen-Vercoe E Bach SJ McAllister TA. Avian- and mammalian-derived antibodies against adherence-associated proteins inhibit host cell colonization by escherichia coli O157:H7. J Appl Microbiol (2007) 103:1206–19. doi: 10.1111/j.1365-2672.2007.03334.x 17897225

[241] Orth D Würzner R. What makes an enterohemorrhagic escherichia coli? Clin Infect Dis (2006) 43:1168–9. doi: 10.1086/508207 17029136

[242] Lee KS Jeong YJ Lee MS. Escherichia coli shiga toxins and gut microbiota interactions. Toxins (Basel) (2021) 13:1–19. doi: 10.3390/toxins13060416 PMC8230793 34208170

[243] Nagy B Fekete PZ. Enterotoxigenic escherichia coli in veterinary medicine. Int J Med Microbiol (2005) 295:443–54. doi: 10.1016/j.ijmm.2005.07.003 16238018

[244] Wang H Zhong Z Luo Y Cox E Devriendt B. Heat-stable enterotoxins of enterotoxigenic escherichia coli and their impact on host immunity. Toxins (Basel) (2019) 11:1–12. doi: 10.3390/toxins11010024 PMC6356903 30626031

[245] Hashish EA Zhang C Ruan X Knudsen DE Chase CC Isaacson RE. A multiepitope fusion antigen elicits neutralizing antibodies against enterotoxigenic escherichia coli and homologous bovine viral diarrhea virus in vitro. Clin Vaccine Immunol (2013) 20:1076–83. doi: 10.1128/CVI.00249-13 PMC3697457 23697572

[246] Fan M He J Liu Y Yang Y Zhou J. Piglet prescription milk powder and preparation method thereof, CN101731365A. (2009).

[247] Ma X Fu C Li Q Li D Wang G Shang A. ETEC (enterotoxigenic escherichla coli) yolk antibody powder and preparation method thereof, CN105713088A. (2016).

[248] Ma Q Li C Li D Wang G Shang A Chen X. Functional egg-milk powder for resisting piglet ETEC (Enterotoxigenic escherichla coli) diarrhea and preparation method thereof, CN106035672A. (2016).

[249] Kariyawasam S Wilkie BN Gyles CL. Construction, characterization, and evaluation of the vaccine potential of three genetically defined mutants of avian pathogenic escherichia coli. Avian Dis (2004) 48:287–99. doi: 10.1637/7093 15283416

[250] Karthikeyan M Indhuprakash ST Gopal G Ambi SV Krishnan UM Diraviyam T. Passive immunotherapy using chicken egg yolk antibody (IgY) against diarrheagenic E. coli: a systematic review and meta-analysis. Int Immunopharmacol (2022) 102:108381. doi: 10.1016/j.intimp.2021.108381 34810126

[251] Foster-Nyarko E Pallen MJ. The microbial ecology of escherichia coli in the vertebrate gut. FEMS Microbiol Rev (2022) 46:1–22. doi: 10.1093/femsre/fuac008 PMC9075585 35134909

[252] Moon HW Bunn TO. Vaccines for preventing enterotoxigenic escherichia coli infections in farm animals. Vaccine (1993) 11:213–20. doi: 10.1016/0264-410X(93)90020-X PMC7130883 8094931

[253] Salverda MLM de Visser JAGM Barlow M. Natural evolution of TEM-1 β-lactamase: experimental reconstruction and clinical relevance. FEMS Microbiol Rev (2010) 34:1015–36. doi: 10.1111/j.1574-6976.2010.00222.x 20412308

[254] Schubert C Oelkrug A. Antibody-mediated neutralization of beta-lactamases, WO2020254861A1. (2019).

[255] Yang S-L Wu Y-Y Leu H-H Shih S-J Huang N-Y Lin I-J. Detection and therapy of bacterial infection caused by enterobacteriaceae, US2010087373A1. (2008).

[256] Du J Mou W Guo S Lu H Wang Y Zhao Y. Yolk antibody for preventing and treating colibacillosis, preparation method for yolk antibody and feed additive, CN102532311A. (2010).

[257] Ochoa TJ Barletta F Contreras C Mercado E. New insights into the epidemiology of enteropathogenic escherichia coli infection. Trans R Soc Trop Med Hyg (2008) 102:852–6. doi: 10.1016/j.trstmh.2008.03.017 PMC2575077 18455741

[258] Ahn JW Kim TY Kim DoG. Antibody for prevention and treatment of enterohemorrhagic E. coli infection, eggs containing thereof and method for producing thereof, KR100471114B1. (2001).

[259] Goepp JG. Treating or preventing travelers diarrhea, WO2021211698A1. (2020).

[260] Roy K Hilliard GM Hamilton DJ Luo J Ostmann MM Fleckenstein JM. Enterotoxigenic escherichia coli EtpA mediates adhesion between flagella and host cells. Nature (2009) 457:594–8. doi: 10.1038/nature07568 PMC2646463 19060885

[261] Peng R Pan J Jiang Z Wang S. Swine enterotoxigenic excherichia coli flagellin 3FliCon fusion protein and application thereof, CN104789589A. (2014).

[262] Peng Z Wang J Jiang R Pan S. Human enterotoxigenic excherichia coli flagellin 2FliC fusion protein and application thereof, CN104789583A. (2014).

[263] Peng J Wang J Jiang S. Recombinant strain for expression of enterotoxin colibacillus adhesin gene and its application in vitelline antibody fodder, CN101113428A. (2007).

[264] He P Han S Yang F Yang Y Qiao S Li D. Egg yolk antibody against pig enterotoxigenic escherichia coli and preparation method thereof, CN109608541A. (2018).

[265] Mehar M Benameur L. Compostable anti-microbial film and method of applying film to packaging, WO2021168581A1. (2020).

[266] Scoggin K Lynch R Gupta J Nagarajan A Sheffield M Elsaadi A. Genetic background influences survival of infections with salmonella enterica serovar typhimurium in the collaborative cross. PloS Genet (2022) 18:1–28. doi: 10.1371/journal.pgen.1010075 PMC9067680 35417454

[267] World Health Organization. Multi-country outbreak of salmonella typhimurium linked to chocolate products – Europe and the united states of America (2022). Available at: https://www.who.int/emergencies/disease-outbreak-news/item/2022-DON369.

[268] Fàbrega A Vila J. Salmonella enterica serovar typhimurium skills to succeed in the host: virulence and regulation. Clin Microbiol Rev (2013) 26:308–41. doi: 10.1128/CMR.00066-12 PMC3623383 23554419

[269] Beal RK Wigley P Powers C Hulme SD Barrow PA Smith AL. Age at primary infection with salmonella enterica serovar typhimurium in the chicken influences persistence of infection and subsequent immunity to re-challenge. Vet Immunol Immunopathol (2004) 100:151–64. doi: 10.1016/j.vetimm.2004.04.005 15207453

[270] Naqid IA Owen JP Maddison BC Spiliotopoulos A Emes RD Warry A. Mapping b-cell responses to salmonella enterica serovars typhimurium and enteritidis in chickens for the discrimination of infected from vaccinated animals. Sci Rep (2016) 6:1–7. doi: 10.1038/srep31186 27510219 PMC4980624

[271] Matulova M Havlickova H Sisak F Babak V Rychlik I. SPI1 defective mutants of salmonella enterica induce cross-protective immunity in chickens against challenge with serovars typhimurium and enteritidis. Vaccine (2013) 31:3156–62. doi: 10.1016/j.vaccine.2013.05.002 23684831

[272] Senevirathne A Hewawaduge C Park S Park JY Kirthika P Lee JH. O-Antigen-deficient, live, attenuated salmonella typhimurium confers efficient uptake, reduced cytotoxicity, and rapid clearance in chicken macrophages and lymphoid organs and induces significantly high protective immune responses that protect chickens ag. Dev Comp Immunol (2020) 111:103745. doi: 10.1016/j.dci.2020.103745 32470560

[273] Galanis E. Campylobacter and bacterial gastroenteritis. C. Can Med Assoc J (2007) 177:570–1. doi: 10.1503/cmaj.070660 PMC1963361 17846438

[274] Butzler JP. Campylobacter, from obscurity to celebrity. Clin Microbiol Infect (2004) 10:868–76. doi: 10.1111/j.1469-0691.2004.00983.x 15373879

[275] Buckley AM Wang J Hudson DL Grant AJ Jones MA Maskell DJ. Evaluation of live-attenuated salmonella vaccines expressing campylobacter antigens for control of c. jejuni in poultry. Vaccine (2010) 28:1094–105. doi: 10.1016/j.vaccine.2009.10.018 19853682

[276] Neal-McKinney JM Samuelson DR Eucker TP Nissen MS Crespo R Konkel ME. Reducing campylobacter jejuni colonization of poultry *via* vaccination. PloS One (2014) 9:1–19. doi: 10.1371/journal.pone.0114254 PMC4256221 25474206

[277] Chintoan-Uta C Cassady-Cain RL Al-Haideri H Watson E Kelly DJ Smith DGE. Superoxide dismutase SodB is a protective antigen against campylobacter jejuni colonisation in chickens. Vaccine (2015) 33:6206–11. doi: 10.1016/j.vaccine.2015.09.100 PMC4654421 26458797

[278] Vohra P Chintoan-uta C Terra VS Bremner A Cuccui J Wren BW. Evaluation of glycosylated FLPA and SODB as subunit vaccines against campylobacter jejuni colonisation in chickens. Vaccines (2020) 8:1–14. doi: 10.3390/vaccines8030520 PMC7564835 32932979

[279] Nothaft H Davis B Lock YY Perez-Munoz ME Vinogradov E Walter J. Engineering the campylobacter jejuni n-glycan to create an effective chicken vaccine. Sci Rep (2016) 6:1–12. doi: 10.1038/srep26511 27221144 PMC4879521

[280] Radomska KA Vaezirad MM Verstappen KM Wösten MMSM Wagenaar JA Van Putten JPM. Chicken immune response after in ovo immunization with chimeric TLR5 activating flagellin of campylobacter jejuni. PloS One (2016) 11:1–15. doi: 10.1371/journal.pone.0164837 PMC5070796 27760175

[281] Singh A Nisaa K Bhattacharyya S Mallick AI. Immunogenicity and protective efficacy of mucosal delivery of recombinant hcp of campylobacter jejuni type VI secretion system (T6SS)in chickens. Mol Immunol (2019) 111:182–97. doi: 10.1016/j.molimm.2019.04.016 31078054

[282] Mortada M Cosby DE Akerele G Ramadan N Oxford J Shanmugasundaram R. Characterizing the immune response of chickens to campylobacter jejuni (strain a74c). PloS One (2021) 16:1–21. doi: 10.1371/journal.pone.0247080 PMC7959354 33720955

[283] Annamalai T Pina-Mimbela R Kumar A Binjawadagi B Liu Z Renukaradhya GJ. Evaluation of nanoparticle-encapsulated outer membrane proteins for the control of campylobacter jejuni colonization in chickens. Poult Sci (2013) 92:2201–11. doi: 10.3382/ps.2012-03004 23873570

[284] Nothaft H Perez-Muñoz ME Yang T Murugan AVM Miller M Kolarich D. Improving chicken responses to glycoconjugate vaccination against campylobacter jejuni. Front Microbiol (2021) 12:734526. doi: 10.3389/fmicb.2021.734526 34867850 PMC8637857

[285] Nothaft H Perez-Muñoz ME Gouveia GJ Duar RM Wanford JJ Lango-Scholey L. Coadministration of the campylobacter jejuni n-glycan-based vaccine with probiotics improves vaccine performance in broiler chickens. Appl Environ Microbiol (2017) 83. doi: 10.1128/AEM.01523-17 PMC5691412 28939610

[286] Łaniewski P Kuczkowski M Chrzastek K Woźniak A Wyszyńska A Wieliczko A. Evaluation of the immunogenicity of campylobacter jejuni CjaA protein delivered by salmonella enterica sv. typhimurium strain with regulated delayed attenuation in chickens. World J Microbiol Biotechnol (2014) 30:281–92. doi: 10.1007/s11274-013-1447-5 PMC3880472 23913025

[287] Lacharme-Lora L Chaloner G Gilroy R Humphrey S Gibbs K Jopson S. B lymphocytes play a limited role in clearance of campylobacter jejuni from the chicken intestinal tract. Sci Rep (2017) 7:2–11. doi: 10.1038/srep45090 28332622 PMC5362810

[288] Buddle JE Fagan RP. Pathogenicity and virulence of clostridioides difficile. Virulence (2023) 14. doi: 10.1080/21505594.2022.2150452 PMC9815241 36419222

[289] Zhang S Xing P Guo G Liu H Lin D Dong C. Development of microbeads of chicken yolk antibodies against clostridium difficile toxin a for colonic-specific delivery. Drug Delivery (2016) 23:1940–7. doi: 10.3109/10717544.2015.1022836 25799315

[290] Feng D Xing P Zhang S Liu H Liu F Wang X. Oral colon-specific preparation for neutralizing anti-clostridium difficile toxin IgY (Immunoglobulin y), CN103690948A. (2014).

[291] Xing P Shi Y Dong C Liu H Cheng Y Sun J. Colon-targeted delivery of IgY against clostridium difficile toxin a and b by encapsulation in chitosan-Ca pectinate microbeads. AAPS PharmSciTech (2017) 18:1095–103. doi: 10.1208/s12249-016-0656-2 27826799

[292] Feng D Xing P Cheng Y Dong C Xu C Sun J. Anti-clostridium difficile toxin IgY colon-specific preparation, CN106474469A. (2017).

[293] Fang L Marquardt RR Sellen RT. Therapeutic clostridium difficile antibody compositions, US2011020356A1. (2007).

[294] Krejčí M Kudláčková J Zouharová H Audová M Tesařík E Gebauer R. A preventive medicine for treating and preventing patients affected by clostridium difficile infection, CZ33428U1. (2019).

[295] Haffajee AD Socransky SS Gunsolley JC. Systemic anti-infective periodontal therapy. a systematic review. Ann Periodontol (2003) 8:115–81. doi: 10.1902/annals.2003.8.1.115 14971252

[296] Rams TE Degener JE van Winkelhoff AJ. Antibiotic resistance in human chronic periodontitis microbiota. J Periodontol (2014) 85:160–9. doi: 10.1902/jop.2013.130142 23688097

[297] Haque MM Yerex K Kelekis-Cholakis A Duan K. Advances in novel therapeutic approaches for periodontal diseases. BMC Oral Health (2022) 22:492. doi: 10.1186/s12903-022-02530-6 36380339 PMC9664646

[298] Lemos JA Palmer SR Zeng L Wen ZT Kajfasz JK Freires IA. The biology of streptococcus mutans. Gram-Positive Pathog (2019) 7(1):435–48. doi: 10.1128/9781683670131.ch27 PMC6615571 30657107

[299] Patel M. Dental caries vaccine: are we there yet? Lett Appl Microbiol (2020) 70:2–12. doi: 10.1111/lam.13218 31518435

[300] Otake S Nishihara Y Makimura M Hatta H Kim M Yamamoto T. Protection of rats against dental caries by passive immunization with hen-egg-yolk antibody (IgY). J Dent Res (1991) 70:162–6. doi: 10.1177/00220345910700030101 1825668

[301] Hatta H Tsuda K Ozeki M Kim M Yamamoto T Otake S. Passive immunization against dental plaque formation in humans: effect of mouth rinse containing egg yolk antibodies (IgY) specific to streptococcus mutans. Caries Res (1997) 31:268–74. doi: 10.1159/000262410 9197932

[302] Chang HM Ou-Yang RF Chen YT Chen CC. Productivity and some properties of immunoglobulin specific against streptococcus mutans serotype c in chicken egg yolk (IgY). J Agric Food Chem (1999) 47:61–6. doi: 10.1021/jf980153u 10563850

[303] Hamada S Horikoshi T Minami T Kawabata S Hiraoka J Fujiwara T. Oral passive immunization against dental caries in rats by use of hen egg yolk antibodies specific for cell-associated glucosyltransferase of streptococcus mutans. Infect Immun (1991) 59:4161–7. doi: 10.1128/iai.59.11.4161-4167.1991 PMC259011 1834573

[304] Hayashi Y Ohara N Ganno T Yamaguchi K Ishizaki T Nakamura T. Chewing chitosan-containing gum effectively inhibits the growth of cariogenic bacteria. Arch Oral Biol (2007) 52:290–4. doi: 10.1016/j.archoralbio.2006.10.004 17112460

[305] Horikoshi T Hiraoka J Fujita I Tokoro T Kodama Y Yokoyama H. Cell-associated glucosyltransferase, an antibody thereto, and a dental caries prophylactic composition containing said antibody as an effective component, US5439680A. (1989).

[306] Horikoshi T Hiraoka J Fujita I Tokoro T Kodama Y Yokoyama H. Antibody and anticarious agent containing said antibody as active ingredient, JPH01242534A. (1988).

[307] Nishida Y Morishima M Ohta M Gomi T Harada Y. Oral composition, US5711937A. (1993).

[308] Zhao S He P Lu M Yang H Wang T. Egg yolk antibody for preventing dental caries, preparation method thereof and egg yolk antibody preparation, CN109593129A. (2018).

[309] Yang RJ Paau S. Combination of IgY against dental caries, US2004126384A1. (2000).

[310] Brennan CA Garrett WS. Fusobacterium nucleatum [[/amp]]mdash; symbiont, opportunist and oncobacterium. Nat Rev Microbiol (2019) 17:156–66. doi: 10.1038/s41579-018-0129-6 PMC6589823 30546113

[311] Chen Y Huang Z Tang Z Huang Y Huang M Liu H. More than just a periodontal pathogen –the research progress on fusobacterium nucleatum. Front Cell Infect Microbiol (2022) 12:815318. doi: 10.3389/fcimb.2022.815318 35186795 PMC8851061

[312] Alkharaan H Lu L Gabarrini G Halimi A Ateeb Z Sobkowiak MJ. Circulating and salivary antibodies to fusobacterium nucleatum are associated with cystic pancreatic neoplasm malignancy. Front Immunol (2020) 11:2003. doi: 10.3389/fimmu.2020.02003 32983143 PMC7484485

[313] Liu PF Shi W Zhu W Smith JW Hsieh SL Gallo RL. Vaccination targeting surface FomA of fusobacterium nucleatum against bacterial co-aggregation: implication for treatment of periodontal infection and halitosis. Vaccine (2010) 28:3496–505. doi: 10.1016/j.vaccine.2010.02.047 PMC2855893 20189489

[314] Ben Lagha A Vaillancourt K Huacho PM Grenier D. Effects of labrador tea, peppermint, and winter savory essential oils on fusobacterium nucleatum. Antibiotics (2020) 9:1–12. doi: 10.3390/antibiotics9110794 PMC7697736 33182686

[315] Ding Q Sun X Cao S Zhao C Wang Y Wang X. Heat-killed lactobacillus acidophilus mediates fusobacterium nucleatum induced pro-inflammatory responses in epithelial cells. FEMS Microbiol Lett (2021) 368. doi: 10.1093/femsle/fnaa160 33693760

[316] Sigusch BW Engelbrecht M Völpel A Holletschke A Pfister W Schütze J. Full-mouth antimicrobial photodynamic therapy in fusobacterium nucleatum-infected periodontitis patients. J Periodontol (2010) 81:975–81. doi: 10.1902/jop.2010.090246 20350153

[317] Nakayama K. Porphyromonas gingivalis and related bacteria: from colonial pigmentation to the type IX secretion system and gliding motility. J Periodontal Res (2015) 50:1–8. doi: 10.1111/jre.12255 25546073 PMC4674972

[318] Andrukhov O Ulm C Reischl H Nguyen PQ Matejka M Rausch-Fan X. Serum cytokine levels in periodontitis patients in relation to the bacterial load. J Periodontol (2011) 82:885–92. doi: 10.1902/jop.2010.100425 21138356

[319] Handal T Caugant DA Olsen I. Antibiotic resistance in bacteria isolated from subgingival plaque in a norwegian population with refractory marginal periodontitis. Antimicrob Agents Chemother (2003) 47:1443–6. doi: 10.1128/AAC.47.4.1443-1446.2003 PMC152519 12654689

[320] Booth V Ashley FP Lehner T. Passive immunization with monoclonal antibodies against porphyromonas gingivalis in patients with periodontitis. Infect Immun (1996) 64:422–7. doi: 10.1128/iai.64.2.422-427.1996 PMC173780 8550186

[321] Ding C Zhang F Gao Y Li Y Cheng D Wang J. Antibacterial photodynamic treatment of porphyromonas gingivalis with toluidine blue O and a NonLaser red light source enhanced by dihydroartemisinin. Photochem Photobiol (2021) 97:377–84. doi: 10.1111/php.13333 32959424

[322] Wang C Li X Cheng T Sun H Jin L. Eradication of porphyromonas gingivalis persisters through colloidal bismuth subcitrate synergistically combined with metronidazole. Front Microbiol (2021) 12:748121. doi: 10.3389/fmicb.2021.748121 34745052 PMC8565575

[323] Park O-J Kwon Y Park C So YJ Park TH Jeong S. Streptococcus gordonii: pathogenesis and host response to its cell wall components. Microorganisms (2020) 8. doi: 10.3390/microorganisms8121852 PMC7761167 33255499

[324] Jia L Han N Du J Guo L Luo Z Liu Y. Pathogenesis of important virulence factors of porphyromonas gingivalis *via* toll-like receptors. Front Cell Infect Microbiol (2019) 9:262. doi: 10.3389/fcimb.2019.00262 31380305 PMC6657652

[325] Oba PM Devito FC Santos JPF Stipp RN de O.S. Gomes M Carciofi AC. Effects of passive immunization by anti-gingipain IgY on the oral health of cats fed kibble diets. J Vet Dent (2018) 35:275–80. doi: 10.1177/0898756418814010

[326] Shi Y Yu X Zhang P. Porphyromonas gingivalis, anti-porphyromonas gingivalis specific IgY preparation and compound toothpaste, CN108728388A. (2018).

[327] Takiguchi T Seki T Suzuki Y Sato Y. Chewing gum, JPH0920684A. (1995).

[328] Kazor CE Mitchell PM Lee AM Stokes LN Loesche WJ Dewhirst FE. Diversity of bacterial populations on the tongue dorsa of patients with halitosis and healthy patients. J Clin Microbiol (2003) 41:558–63. doi: 10.1128/JCM.41.2.558-563.2003 PMC149706 12574246

[329] Alauzet C Aujoulat F Lozniewski A Ben Brahim S Domenjod C Enault C. A new look at the genus solobacterium: a retrospective analysis of twenty-seven cases of infection involving s. moorei and a review of sequence databases and the literature. Microorganisms (2021) 9(6). doi: 10.3390/microorganisms9061229 PMC8229177 34198943

[330] Barrak I Stájer A Gajdács M Urbán E. Small, but smelly: the importance of solobacterium moorei in halitosis and other human infections. Heliyon (2020) 6:e05371. doi: 10.1016/j.heliyon.2020.e05371 33163658 PMC7610269

[331] Li X Liu H Xu Y Xu F Wang L You J. Chicken egg yolk antibody (IgY) controls solobacterium moorei under *in vitro* and *in vivo* conditions. Appl Biochem Biotechnol (2012) 168:1448–58. doi: 10.1007/s12010-012-9869-3 22968588

[332] Xu Y Li X Xu F Jin L Wang X Zhen Y. Specific yolk antibody preparation for controlling halitosis pathogens and application thereof, CN101564534A. (2008).

[333] Hindy J-R Quintero-Martinez JA Lee AT Scott CG Gerberi DJ Mahmood M. Incidence trends and epidemiology of staphylococcus aureus bacteremia: a systematic review of population-based studies. Cureus (2022) 14. doi: 10.7759/cureus.25460 PMC9239286 35774691

[334] Cong Y Yang S Rao X. Vancomycin resistant staphylococcus aureus infections: a review of case updating and clinical features. J Adv Res (2020) 21:169–76. doi: 10.1016/j.jare.2019.10.005 PMC7015472 32071785

[335] Foster TJ. Antibiotic resistance in staphylococcus aureus. current status and future prospects. FEMS Microbiol Rev (2017) 41:430–49. doi: 10.1093/femsre/fux007 28419231

[336] Guo Y Song G Sun M Wang J Wang Y. Prevalence and therapies of antibiotic-resistance in staphylococcus aureus. Front Cell Infect Microbiol (2020) 10:107. doi: 10.3389/fcimb.2020.00107 32257966 PMC7089872

[337] Parquet MDC Savage KA Allan DS Davidson RJ Holbein BE. Novel iron-chelator DIBI inhibits staphylococcus aureus growth, suppresses experimental MRSA infection in mice and enhances the activities of diverse antibiotics in vitro. Front Microbiol (2018) 9:1811. doi: 10.3389/fmicb.2018.01811 30154764 PMC6103240

[338] Plumet L Ahmad-Mansour N Dunyach-Remy C Kissa K Sotto A Lavigne J-P. Bacteriophage therapy for staphylococcus aureus infections: a review of animal models, treatments, and clinical trials. Front Cell Infect Microbiol (2022) 12:907314. doi: 10.3389/fcimb.2022.907314 35782148 PMC9247187

[339] Zhou K Li C Chen D Pan Y Tao Y Qu W. A review on nanosystems as an effective approach against infections of staphylococcus aureus. Int J Nanomed (2018) 13:7333–47. doi: 10.2147/IJN.S169935 PMC6233487 30519018

[340] Sedlmayer F Woischnig A-K Unterreiner V Fuchs F Baeschlin D Khanna N. 5-fluorouracil blocks quorum-sensing of biofilm-embedded methicillin-resistant staphylococcus aureus in mice. Nucleic Acids Res (2021) 49:e73–3. doi: 10.1093/nar/gkab251 PMC8287944 33856484

[341] Rainard P Gilbert FB Germon P Foucras G. Invited review: a critical appraisal of mastitis vaccines for dairy cows. J Dairy Sci (2021) 104:10427–48. doi: 10.3168/jds.2021-20434 34218921

[342] Kobayashi SD DeLeo FR. Staphylococcus aureus protein a promotes immune suppression. MBio (2013) 4:e00764–13. doi: 10.1128/mBio.00764-13 PMC3791897 24085782

[343] Grzywa R Walczak M Łupicka-Słowik A Bobrek K Boivin S Brown EL. Adjuvant-dependent immunogenicity of staphylococcus aureus efb and map proteins in chickens. Vet Immunol Immunopathol (2015) 166:50–6. doi: 10.1016/j.vetimm.2015.04.009 26004944

[344] Li J Xu Y Wang X Li Y Wang L Li X. Construction and characterization of a highly reactive chicken-derived single-chain variable fragment (scFv) antibody against staphylococcus aureus developed with the T7 phage display system. Int Immunopharmacol (2016) 35:149–54. doi: 10.1016/j.intimp.2016.02.024 27046516

[345] Jin W Yamada K Ikami M Kaji N Tokeshi M Atsumi Y. Application of IgY to sandwich enzyme-linked immunosorbent assays, lateral flow devices, and immunopillar chips for detecting staphylococcal enterotoxins in milk and dairy products. J Microbiol Methods (2013) 92:323–31. doi: 10.1016/j.mimet.2013.01.001 23318552

[346] Reddy PK Shekar A Kingston JJ Sripathy MH Batra H. Evaluation of IgY capture ELISA for sensitive detection of alpha hemolysin of staphylococcus aureus without staphylococcal protein a interference. J Immunol Methods (2013) 391:31–8. doi: 10.1016/j.jim.2013.02.004 23454246

[347] Yamada K Wanchun J Ohkura T Murai A Hayakawa R Kinoshita K. Detection of methicillin-resistant staphylococcus aureus using a specific anti-PBP2a chicken IgY antibody. Jpn J Infect Dis (2013) 66:103–8. doi: 10.7883/yoken.66.103 23514905

[348] Reddy P Ramlal S Sripathy MH Batra HV. Development and evaluation of IgY ImmunoCapture PCR ELISA for detection of staphylococcus aureus enterotoxin a devoid of protein a interference. J Immunol Methods (2014) 408:114–22. doi: 10.1016/j.jim.2014.05.012 24941881

[349] Nagaraj S Ramlal S Kingston J Batra HV. Development of IgY based sandwich ELISA for the detection of staphylococcal enterotoxin G (SEG), an egc toxin. Int J Food Microbiol (2016) 237:136–41. doi: 10.1016/j.ijfoodmicro.2016.08.009 27569376

[350] Pang B Zheng Y Wang J Liu Y Song X Li J. Colorimetric detection of staphylococcus aureus using gold nanorods labeled with yolk immunoglobulin and urease, magnetic beads, and a phenolphthalein impregnated test paper. Microchim Acta (2019) 186. doi: 10.1007/s00604-019-3722-0 31396712

[351] Zhang Y Tan W Zhang Y Mao H Shi S Duan L. Ultrasensitive and selective detection of staphylococcus aureus using a novel IgY-based colorimetric platform. Biosens Bioelectron (2019) 142:111570. doi: 10.1016/j.bios.2019.111570 31401227

[352] Roushani M Rahmati Z Golchin M Lotfi Z Nemati M. Electrochemical immunosensor for determination of staphylococcus aureus bacteria by IgY immobilized on glassy carbon electrode with electrodeposited gold nanoparticles. Microchim Acta (2020) 187. doi: 10.1007/s00604-020-04547-6 32929566

[353] Yao S Li J Pang B Wang X Shi Y Song X. Colorimetric immunoassay for rapid detection of staphylococcus aureus based on etching-enhanced peroxidase-like catalytic activity of gold nanoparticles. Microchim Acta (2020) 187. doi: 10.1007/s00604-020-04473-7 32813037

[354] Gan W Xu Z Li Y Bi W Chu L Qi Q. Rapid and sensitive detection of staphylococcus aureus by using a long-period fiber grating immunosensor coated with egg yolk antibody. Biosens Bioelectron (2022) 199:113860. doi: 10.1016/j.bios.2021.113860 34890885

[355] Urisu A Kondo Y Tsuge I. Hen ‘ s egg allergy. Chem Immunol Allergy (2015) 101:124–30. doi: 10.1159/000375416 26022872

[356] Hewlings SJ. Use of a hyperimmune egg product to prevent and treat dysbiosis, US10632158B2. (2020).

[357] Surcel M Munteanu A Isvoranu G Ibram A Caruntu C Constantin C. Unconventional therapy with IgY in a psoriatic mouse model targeting gut microbiome. J Pers Med (2021) 11:841. doi: 10.3390/jpm11090841 34575618 PMC8466815

[358] Sahoo DK Allenspach K Mochel JP Parker V Rudinsky AJ Winston JA. Synbiotic-IgY therapy modulates the mucosal microbiome and inflammatory indices in dogs with chronic inflammatory enteropathy: a randomized, double-blind, placebo-controlled study. Vet Sci (2022) 10:25. doi: 10.3390/vetsci10010025 36669027 PMC9867299

[359] Butz S Frank C Berger W. Methods of treating fibromyalgia, WO2020147950A1. (2019).

[360] Maddox E Massoni S Hoffart C Takata Y. Dietary effects on pain symptoms in patients with fibromyalgia Syndrome: systematic review and future directions. Nutrients (2023) 15:716. doi: 10.3390/nu15030716 36771421 PMC9921865

[361] Starzl W. Compositions and methods for treatment in broad-spectrum, undifferentiated or mixed clinical applications, WO2012071346A1. (2010). Timothy.

[362] Grabowsky M Playford RJ Starzl TW Marchbank T Kelly P Choudhry N. Compositions for management of disorders of the gastrointestinal tract, WO2020176637. (2019).

[363] Gaensbauer JT Melgar MA Calvimontes DM Lamb MM Asturias EJ Contreras-roldan IL. Efficacy of a bovine colostrum and egg- based intervention in acute childhood diarrhoea in Guatemala: a randomised. BMJ Glob Heal (2017) 2:e000452. doi: 10.1136/bmjgh-2017-000452 PMC5728299 29259822

[364] Playford RJ Choudhry N Kelly P Marchbank T. Effects of bovine colostrum with or without egg on *In vitro* bacterial-induced intestinal damage with relevance for SIBO and infectious diarrhea. Nutrients (2021) 13:1024. doi: 10.3390/nu13031024 33809940 PMC8004259

[365] Baek BS Lee NH Sunwoo SY. The method for production of egg containing anti-E. coli IgY and anti-H. pylori IgY simultaneously and egg yogurt and ice-cream containing specific IgY for anti-E. coli and anti-H. pylori, KR100415911B1. (2000).

[366] Lee N-H Ryu J-S Jung K-Y Baek B-S Sunwoo S-Y. The method for the production of the egg containing anti-pathogenic bacteria specific antibodies (IgY) and the yogurt and ice cream containing the IgY, WO02053179A1. (2001).

[367] Choi IS Choi SU Jung SH Kim MH Lee GS Lee NH. The production method of kimchi containing immunoglobulin y for e.coli, helicobacter pylori and thereof kimchi, KR100485269B1. (2002).

[368] Lee HS Baek NH Cho BS Choi SUn Jun UnN. The method for soy sauce containing anti-E. coli AND anti-H. pylori specific IgY, KR100501204B1. (2003).

[369] Baek BS Cho SU Choi UN Jun HS Lee NH Sunwoo SY. The method for soybean paste containing anti-E. coli AND anti-H. pylori specific IgY, KR100501205B1. (2001).

[370] Baek BS Lee NH Sunu SY. Fruit juice containing specific IgY for anti-E. coli and anti-H. pylori, KR100421309B1. (2001).

[371] Bao T Wang S Cai Z. Milk product with specific immunity of anti-enterobacter sakazakii and the preparing method thereof, CN101040632A. (2006).

[372] Song M-S Yi H-J Cho W-I Sohn K-H Yoon H-N. Growth inhibitory composition against pathogenic bacteria of meat based food stuff comprising IgY, WO2007105894A1. (2006).

[373] Paau S Yang R-J. Preparation method of IgY for preventing and cure mouth disease and the toothpaste base on the IgY, US2006198849A1. (2003).

[374] Chen J Luo X Sun X Zhang P. IgY antibody preparation having mixed specificity for resistance to streptococcus mutans and streptococcus sobrinus, and preparation method and IgY total toothpaste, CN102973939A. (2012).

[375] Chen J Luo X Sun X Zhang P. Anti-porphyromonas gingivalis and fusobacterium nucleatum compound specific IgY antibody, preparation method and toothpaste thereof, CN103007278A. (2012).

[376] Zhao S Jiao L Hu Q Lyu R Xia N Liang Y. Egg yolk antibody mouth wash capable of preventing and treating ozostomia and preparation method thereof, CN106214510A. (2016).

[377] Chen P Luo J Sun X Zhang X. Gingivitis and gingivitis ozostomia preventing mouthwash prepared by anti-porphyromonas gingivalis and IgY antibody with fusobacterium nucleatum specificity, CN102860932A. (2012).

[378] Pang Y Wu B Wei Y. Oral cavity spray as well as preparation method and application thereof, CN108714215A. (2018).

[379] Zou J Jia C. Composite specific yolk antibody oral cavity pressurized spray and preparation method thereof, CN110237251A. (2019).

[380] Meng Y Fu M Nong D Meng X Yang B Nong R. Medical chewing gum and preparation method thereof, CN109010822A. (2018).

[381] Oka H. Tooth coating composite and its preparation, EP0900560A1. (1997).

[382] Sheng B Yanbo X Wang C Tingyin C Bao H. Method for preparing immune milk or milk powder containing IgY antibody, CN1965665A. (2005).

[383] Chao L Zhenqiang Q. Pharmaceutical composition and composite burn cream for treating burns and scalds and preparation method thereof, CN103357011A. (2013).

[384] Lin X. Quick extraction method for egg yolk antibody, prepared anti-burn, anti-scald and anti-infection product and application of egg yolk antibody, CN108640988A. (2018).

[385] Yongxiang M Meiyan F Nong D. Antibacterial cleaning composition and wound plaster for wounds, CN110787293A. (2018).

[386] Meng Y Fu M Nong D Ye M. Specific yolk immunoglobulin composition and preparation thereof, CN109010824A. (2018).

[387] Fu Y. Preparation method of composite IgY antigen against propionibacterium acnes and staphylococcus and application of composite IgY antigen, CN110680917A. (2019).

[388] Nong D Fu M Meng X Meng Y Xie M Wei L. Skin care composition having antibacterial and acne removing effect, and applications thereof, CN108619012A. (2017).

[389] Meng X Fu M Meng Y Nong D Xie M Wei L. Composite antibody extract, preparation method and applications thereof, CN108623680A. (2017).

[390] Nong D Meng Y Fu M Ye M. Composite egg yolk antibody composition for preventing and treating respiratory tract infection, atomized inhalation solution, preparation process and application thereof, CN108992669A. (2018).

[391] Jiang X Li J Jiang S Jiang H. Application of preparing throat-moistening health-care product from chicken egg yolk immunoglobulin, CN112957464A. (2021).

[392] Fu M Meng Y Nong D Ye M. Vaginal foaming agent and application thereof, CN108653729A. (2018).

[393] Fu M Meng Y Nong D Ye M. Vaginal in-situ gel preparation, and preparation method and application thereof, CN109010825A. (2018).

[394] Zhang H. Externally used compound preparation for gynecological inflammation, CN111789945A. (2020).

[395] Duan Z Meng X Zhao Q Chen W Xie M Wei L. Specificity yelk immune globulin composition for preventing pathogenic bacteria and preparation thereof, CN104013958A. (2014).

[396] Uasa K Sato F Kodama Y Nguyen VS Odamaki T Shimizu K. Anticarious composition, JP2008247750A. (2007).

[397] Kodama Y Nguyen SV Ke Am SRA Yokoyama H Goshima H. Composition for treating or preventing periodontal infection, JP2011016843A. (2010).

[398] Cheong HG Baek DY Won M-K Bae H-D. Method for preparing anti-helicobacter pylori egg yolk antibody, WO2021100992A1. (2021).

[399] Cheong HG Won M-K Al E. IgY against helicobacter pylori, KR101947014B1. (2017).

[400] Hong Gul EJC Hwan L Sun Jae L Doo Yeon B. Cosmetic composition containing IgY from egg yolk for improvement of acene, KR101085540B1. (2009).

[401] Ahn TY Cho IG Kim DG Kim JW Yang SY. Antibody protein for prevention and treatment of helicobacter pylori infection, eggs containing the same and production thereof, KR20040081230A. (2003).

[402] Ark JB Kim JW Yang SY Jang MO Shin SO. Composition for prevention, alleviation and treatment of atopyic dermatitis, WO2006104336A1. (2005).

[403] Tsukamoto Y Maeda O Shigekawa G Greenberg S Hendler B. Ostrich antibody and its application to skin diseases. Rev Case Report Health (Irvine Calif) (2018) 10:1357–70. doi: 10.4236/health.2018.1010105

[404] Tsukamoto Y. Antibody and antibody-containing composition, WO2013027356A1. (2011).

[405] Wu Q. Medical dental cream for oral health care of children and preparation method thereof, CN101756878A. (2010).

[406] Gupta PK. New disease old vaccine: is recombinant BCG vaccine an answer for COVID-19? Cell Immunol (2020) 356:104187. doi: 10.1016/j.cellimm.2020.104187 32745670 PMC7386780

[407] Rodriguez-Campos S Smith NH Boniotti MB Aranaz A. Overview and phylogeny of mycobacterium tuberculosis complex organisms: implications for diagnostics and legislation of bovine tuberculosis. Res Vet Sci (2014) 97:S5–S19. doi: 10.1016/j.rvsc.2014.02.009 24630673

[408] de Bono JS Rha SY Stephenson J Schultes BC Monroe P Eckhardt GS. Phase I trial of a murine antibody to MUC1 in patients with metastatic cancer: evidence for the activation of humoral and cellular antitumor immunity. Ann Oncol (2004) 15:1825–33. doi: 10.1093/annonc/mdh472 15550589

[409] Ahn TY Kim DG Kim JW. Soluble protein for prevention and treatment of enteropathogenic e.coli infection, eggs containing thereof and method for producing thereof, KR100471115B1. (2001).

[410] Kim DG Kim JW. Soluble protein for prevention and treatment of enterotoxigenic E. coli infection, eggs containing the same and method for producing thereof, KR100471116B1. (2001).

[411] Kim JU. Egg yolk antibody against salmonella, KR20020032772A. (2000).

[412] Seiti Yamada Yoshikawa F Feitosa de Lima J Notomi Sato M Álefe Leuzzi Ramos Y Aoki V Leao Orfali R. Exploring the role of staphylococcus aureus toxins in atopic dermatitis. Toxins (Basel) (2019) 11:321. doi: 10.3390/toxins11060321 31195639 PMC6628437

[413] Kodama Y Yokoyama H. Antidiarrheal composition, WO2004052379A1. (2002).

[414] Han Y Zhan T Tang C Zhao Q Dansou DM Yu Y. Effect of replacing in-feed antibiotic growth promoters with a combination of egg immunoglobulins and phytomolecules on the performance, serum immunity, and intestinal health of weaned pigs challenged with escherichia coli k88. Animals (2021) 11. doi: 10.3390/ani11051292 PMC8146111 33946355

[415] Yokoyama H Peralta RC Diaz R Sendo S Ikemori Y Kodama Y. Passive protective effect of chicken egg yolk immunoglobulins against experimental enterotoxigenic escherichia coli infection in neonatal piglets. Infect Immun (1992) 60:998–1007. doi: 10.1128/iai.60.3.998-1007.1992 1347289 PMC257586

[416] Marquardt RR Jin L Kim J-W Fang L Frohlich AA Baidoo SK. Passive protective effect of egg-yolk antibodies against enterotoxigenic escherichia coli K88+ infection in neonatal and early-weaned piglets. FEMS Immunol Med Microbiol (1999) 23:283–8. doi: 10.1111/j.1574-695X.1999.tb01249.x 10225287

[417] Cheong HG Kwon HS Kim SY Yang SY. Antibody against shrimp early mortality syndrome and white spot virus, and use thereof, WO2020027381A1. (2018).

[418] Cheong HG Ahn HC Kang YM. Method of preparing egg yolk antibody for preventing death in shrimp, WO2021101132A2. (2019).

[419] Cheong HG Kang YM Bae HD. Manufacturing method of immunoglobulin y for preventing or treating salmon rickettisia septicaemia, KR102200721B1. (2019).

[420] Valenzuela-Aviles P Torrealba D Figueroa C Mercado L Dixon B Conejeros P. Why vaccines fail against piscirickettsiosis in farmed salmon and trout and how to avoid it: a review. Front Immunol (2022) 13:1019404. doi: 10.3389/fimmu.2022.1019404 36466828 PMC9714679

[421] Cheong HG. Manufacturing method of immunoglobulin y for preventing or treating calf digestive diseases and immunoglobulin y thereby and the use thereof, KR102047784B1. (2018).

[422] Jeong H.-G. Manufacturing method of immunoglobulin y for preventing or treating pig digestive diseases and immunoglobulin y thereby and the use thereof, KR102148503B1. (2018).

[423] Park JK Sung JH Won MK Kim BM Cheong HG Lim H. Composition of food additives of piglets containing IgY from egg yolk for preventing of porcine epidemic diarrhea or transmissible gastroenteritis, KR101046001B1. (2009).

[424] Cheong HG Lim H Baek DY Won MK Kim BM Han SM. Composition of feed additives for ducks comprising egg yolk containing IgY against pathogen to induce acute and chronic infection disease, KR101127171B1. (2009).

[425] Victor PI Viorica C Constantin C Georgiana T Victor PI Viorica C. Procedure to obtain and use hen egg immunoglobulins (IgY), RO129645A0. (2014).

[426] Victor PI Viorica C Constantin C Georgiana T. Method for immunobiological assay of chicken immunoglobulins specific activity, RO129677A0. (2014).

[427] Parlasca M Knößlsdorfer I Alemayehu G Doyle R. How and why animal welfare concerns evolve in developing countries. Anim Front (2023) 13:26–33. doi: 10.1093/af/vfac082 36845609 PMC9947326

